# Different Antioxidant Efficacy of Two Mn^II^-Containing Superoxide Anion Scavengers on Hypoxia/Reoxygenation-Exposed Cardiac Muscle Cells

**DOI:** 10.1038/s41598-019-46476-2

**Published:** 2019-07-16

**Authors:** Matteo Becatti, Andrea Bencini, Silvia Nistri, Luca Conti, Maria Giulia Fabbrini, Laura Lucarini, Veronica Ghini, Mirko Severi, Claudia Fiorillo, Claudia Giorgi, Lorenzo Sorace, Barbara Valtancoli, Daniele Bani

**Affiliations:** 1 Department of, Experimental & Clinical Biomedical Sciences “Mario Serio”, Section of Biochemical Sciences, University of Florence, viale G.B. Morgagni 50, 50134 Florence, Italy; 2 Department of Chemistry “Ugo Schiff”, University of Florence, via della Lastruccia 3, 50019 Sesto Fiorentino, Florence Italy; 3 Department of Experimental & Clinical Medicine, Research Unit of Histology & Embryology, University of Florence, viale G. Pieraccini 6, 50139 Florence, Italy; 4 Department NEUROFARBA, Section of Pharmacology, University of Florence, viale G. Pieraccini 6, 50139 Florence, Italy; 5 Center of Magnetic Resonance (CERM), University of Florence, Sesto Fiorentino, Florence Italy

**Keywords:** Structure-based drug design, Pharmacology, Organometallic chemistry

## Abstract

Oxidative stress due to excess superoxide anion (${{\bf{O}}}_{{\bf{2}}}^{{\boldsymbol{\cdot }}{\boldsymbol{-}}}$) produced by dysfunctional mitochondria is a key pathogenic event of aging and ischemia-reperfusion diseases. Here, a new ${{\bf{O}}}_{{\bf{2}}}^{{\boldsymbol{\cdot }}{\boldsymbol{-}}}$-scavenging Mn^II^ complex with a new polyamino-polycarboxylate macrocycle (4,10-dimethyl-1,4,7,10-tetraazacyclododecane-1,7-diacetate) containing 2 quinoline units (MnQ2), designed to improve complex stability and cell permeability, was compared to parental Mn^II^ complex with methyls replacing quinolines (MnM2). MnQ2 was more stable than MnM2 (log *K* = 19.56(8) vs. 14.73(2) for the equilibrium Mn^2+^ + L^2−^, where L = Q2 and M2) due to the involvement of quinoline in metal binding and to the hydrophobic features of the ligand which improve metal desolvation upon complexation. As oxidative stress model, H9c2 rat cardiomyoblasts were subjected to hypoxia-reoxygenation. MnQ2 and MnM2 (10 μmol L^−1^) were added at reoxygenation for 1 or 2 h. The more lipophilic MnQ2 showed more rapid cell and mitochondrial penetration than MnM2. Both MnQ2 and MnM2 abated endogenous ROS and mitochondrial ${{\bf{O}}}_{{\bf{2}}}^{{\boldsymbol{\cdot }}{\boldsymbol{-}}}$, decreased cell lipid peroxidation, reduced mitochondrial dysfunction, in terms of efficiency of the respiratory chain and preservation of membrane potential (Δψ) and permeability, decreased the activation of pro-apoptotic caspases 9 and 3, and increased cell viability. Of note, MnQ2 was more effective than MnM2 to exert cytoprotective anti-oxidant effects in the short term. Compounds with redox-inert Zn^II^ replacing the functional Mn^II^ were ineffective. This study provides clues which further our understanding of the structure-activity relationships of Mn^II^-chelates and suggests that Mn^II^-polyamino-polycarboxylate macrocycles could be developed as new anti-oxidant drugs.

## Introduction

In the course of time, the traditional free radical theory of aging, which emphasizes the role of reactive oxygen species (ROS) and, more broadly, pro-oxidant reactive species (RS) generated in the cellular environment in the process of aging and in the determination of lifespan^1^, has been challenged by the inconclusive results of experimental studies and clinical trials aimed at slowing ageing and extending the lifespan of the organism by supplementation of antioxidants or potentiation of the RS-scavenging systems by genetic manipulation^2–4^. Thus, the original hypothesis has been revised in form of the mitochondrial-free radical theory, in which mitochondrially-generated ROS have been postulated as the major effectors of aging^5–7^. Many subsequent studies have strengthened this hypothesis, showing that potentiation of mitochondrial anti-oxidant systems in laboratory animals significantly extends their lifespan^8^, while the opposite occurs in mutant *Drosoplila* lacking mitochondrial superoxide dismutase (SOD), a major cell antioxidant enzyme^9^. This background knowledge has prompted the identification and synthesis of new antioxidant molecules with improved capability to enter the cells and target mitochondria: these compounds have been proven effective to delay aging in cell culture models, invertebrates and mammals^10,11^.

Superoxide anion (${{\rm{O}}}_{2}^{\cdot -}$) is the paradigmatic ROS produced in mitochondria during oxidative metabolism. Under physiological conditions, its levels are kept under the threshold for oxidative damage by multiple redox pathways, among which a key role is played by SODs, which catalyze ${{\rm{O}}}_{2}^{\cdot -}$ dismutation to O_2_ and H_2_O_2_ exploiting a transition metal, usually a Cu or Mn metal ion, at the catalytic site^12^. The same Mn^II^ ion is the functional core of the non-peptidic, low molecular weight anti-oxidant compounds known as SOD-mimetics^13^. Several Mn^II^/Mn^III^-chelating organic scaffolds, such as porphyrins, salen and its derivatives, cyclic polyamines and polyamine-polycarboxylates, have been successfully tested as possible new anti-oxidant drugs in diverse cellular and animal models of oxidative stress^14–16^. In our previous research we focused on a dimethylated Mn^II^-polyamine-polycarboxylate compound [Mn^II^(4,10-dimethyl-1,4,7,10-tetraazacyclododecane-1,7-diacetate)·2H_2_O], here abbreviated as MnM2, endowed with potent ${{\rm{O}}}_{2}^{\cdot -}$ scavenging properties and capability to reduce endogenous and exogenous oxidative stress^17–19^. Polyamine-polycarboxylate scaffolds are optimal chelants since they can form highly stable complexes with metal ions, including Mn^II^, resistant to oxidation and reduction and virtually devoid of cellular toxicity^17,20,21^. Moreover, the organic scaffold can be easily modified by substitution of the two methyl groups in position 4,10 with hydrophilic or lipophilic moieties, thereby modulating the distribution of such compounds in the extracellular or intracellular compartments and hence their antioxidant efficacy^22^. All these properties render these compounds a promising new category of redox-active drugs for experimental and medicinal purposes.

**Figure 1:**
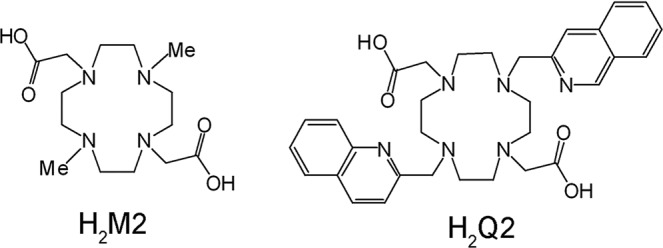
Drawings of H_2_M2 and H_2_Q2.

On this ground, we have introduced two quinoline units as pendant arms of a 1,4,7,10-tetraazacyclododecane-1,7-diacetate macrocycle, which replace the 2 methyl groups of MnM2 (Fig. 1). In principle, this new Mn^II^ complex would gain increased lipophilicity, thus increasing its capability to enter the cells and decompose ${{\rm{O}}}_{2}^{\cdot -}$ at the mitochondrial generation site. At the same time, quinolines possess a heteroaromatic nitrogen which could be involved in metal binding, thus increasing stability of the Mn^II^ complex and reducing the risk of possible decomplexation and release of Mn^II^. We have then analyzed the antioxidant efficacy of the new MnQ2 [Mn^II^(4,10-diquinolyl-1,4,7,10-tetraazacyclododecane-1,7-diacetate)·2H_2_O], comparing its effects with those of the parental compound MnM2. As cellular model of endogenous oxidative stress, we have used H9c2 rat cardiac myoblasts subjected to hypoxia followed by reoxygenation^18^. As control substances, we have used the inactive congeners ZnM2 and ZnQ2, in which the functional Mn^II^ was replaced with redox-inert Zn^II^.

## Results

### Synthesis of 4,10-diquinolyl-1,4,7,10-tetraazacyclododecane-1,7-diacetate (H_2_Q2) and its Mn^II^ and Zn^II^ complexes (MnQ2 and ZnQ2)

**Figure 2:**
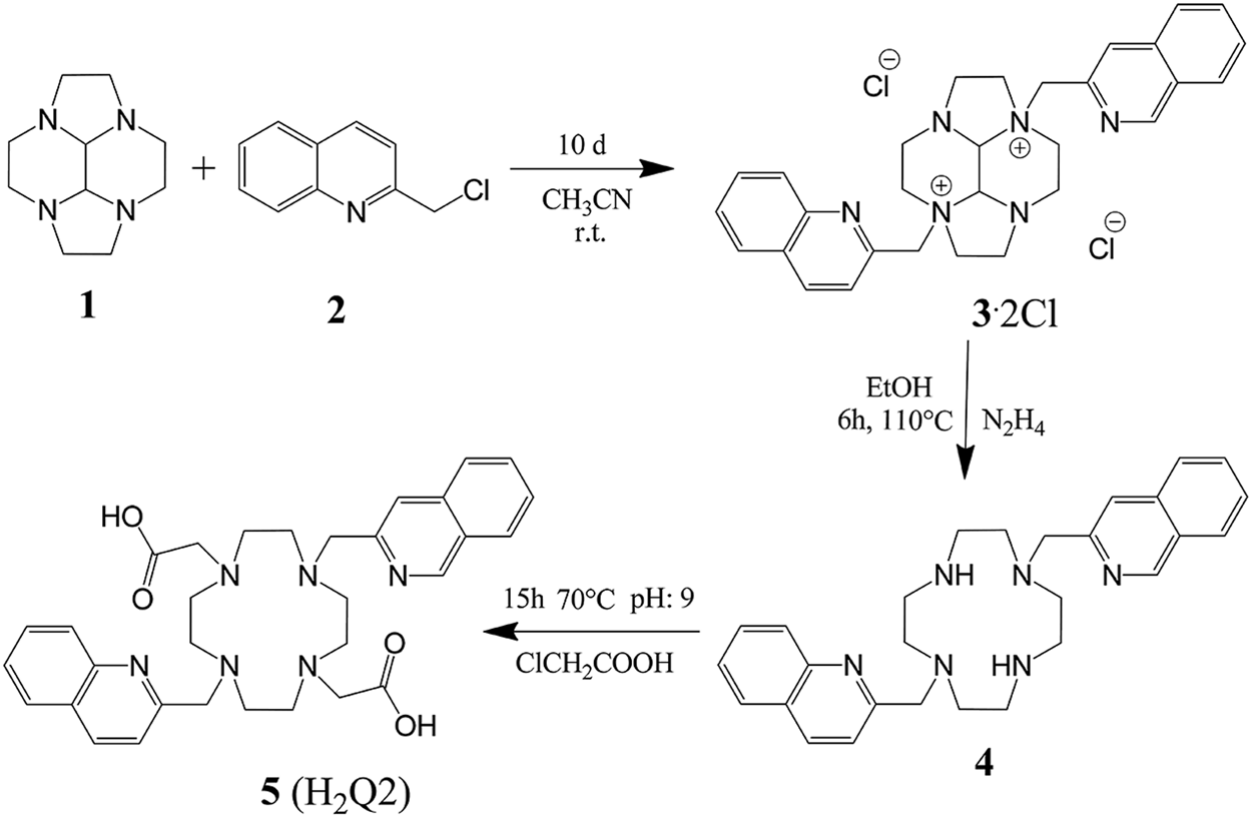
Synthetic procedure used for H_2_Q2 synthesis.

The synthesis of H_2_Q2 was performed using the bis-aminal procedure, which allows easy polyfunctionalization of cyclic tetraamines^23^. In fact, the bisaminal derivatives of these compounds, including cyclen (cyclen = 1,4,7,10-tetraazacyclododecane), show a bent conformation inducing different reactivity of their tertiary amine groups toward electrophilic agents. The 2 amine groups in opposite positions feature their lone pair directed toward the convex side of the molecular framework, making them poorly available for reaction with electrophiles. Conversely, the lone pairs of the 2 remaining couples are directed outwards the cyclic framework, positioned to easily react with electrophiles. Therefore, reaction of the bisaminal derivative decahydro-2a,4a,6a,8a-tetraaza-cyclopenta[fg]acenaphthylene **1** with 2-chloromethylen-quinoline **2** in CH_3_CN affords as unique product, the chloride salt of the ammonium cation **3**, in good yields (>70%) (Fig. 2). This procedure takes advantage from the extremely low solubility of the salt, which can be isolated by simple filtration from the reaction mixture. Reagent **3** resulted practically pure and was used in the following reaction without further purification. Deprotection of the bisaminal **3** with hydrazine affords macrocycle **4**, which contains two secondary amine groups. The latter can be easily functionalized with methylacetate groups upon reaction with chloroacetic acid at pH 9. The final product **5** (H_2_Q2) was purified by ionic exchange column chromatography, which allows removal of NaCl, formed in the previous reaction. The MnQ2 and ZnQ2 complexes were prepared by mixing solutions of ligands and metals at neutral pH. A de-aerated environment was used for the Mn^II^ complex to prevent its oxidation by atmospheric O_2_. Both complexes show a good solubility in water (about 10^−2^ M). All compounds were characterized by elemental analysis and high resolution mass spectrometry. ^1^H and ^13^C NMR spectra were also collected for compounds **3–5** and ZnQ2, while Mn^II^ complexation was analyzed only by ^1^H NMR, due to the paramagnetic nature of this ion. Both elemental analysis and mass spectrometry measurements accounted for the formation of Mn^II^ and Zn^II^ complex with metal to ligand 1:1 stoichiometry. The NMR and mass spectra are reported in the Supplementary Information 1 (Figs S1–S30).

### Metal binding by H_2_Q2 and H_2_M2 and stability of their complexes

Both these organic scaffolds contain a tetra-amine macrocyclic ring and two appended carboxylic groups, making them potential chelating agents for both transition, alkali and alkaline metal ions. In addition, H_2_Q2 contains two quinoline moieties. Quinoline is also able to coordinate transition metals, including Mn^II^, although its binding ability is far lower than aliphatic amine groups. At the same time, this heterocycle shows poor affinity for alkali and alkaline earth metals, which result essentially not bound at least in aqueous solution^24^. On this ground, we first investigated Mn^II^ coordination in aqueous solution by means of potentiometric titrations. This study requires preliminary determination of the protonation constants of the ligands. These values were in the range generally observed for polyamino-polycarboxylic acids^20^ and are reported in Table 1, together with the complexes formed by H_2_Q2 in solution and their formation constants and those formed by H_2_M2, determined in a previous study^17^.

**Table 1:** Stability constants of the H_2_M2 and H_2_Q2 complexes with Mn^II^ and other cations (Na^I^, Mg^II^, Ca^II^) and protonation constants of H_2_Q2 in aqueous solution (I = 0.1 M, 25 °C).

Reaction	log *K*	
Q2^2−^ + 2 H^+^ = H_2_Q2	20.97(3)	
H_2_Q2 + H^+^ = H_3_Q2^+^	6.67(8)	
H_3_Q2^+^ + H^+^ = H_4_Q2^2+^	6.33(5)	
H_4_Q2^2+^ + H^+^ = H_5_Q2^3+^	4.23(6)	
H_5_Q2^3+^ + H^+^ = H_6_Q2^4+^	3.41(6)	
Mn^2+^ + M2^2−^ = [MnM2]	14.73(2)	
[MnM2] + H^+^ = [Mn(HM2)]^+^	4.53(7)	
[Mn(HM2)]^+^ + H^+^ = [Mn(H_2_M2)]^2+^	3.96(2)	
Mn^2+^ + Q2^2−^ = [MnQ2]	19.56(8)	
[MnQ2] + H^+^ = [Mn(HQ2)]^+^	4.50(2)	
[Mn(HQ2)]^+^ + H^+^ = [Mn(H_2_Q2)]^2+^	4.31(1)	
	**L**^**2−**^ **=** **Me2**^**2−**^	**L**^**2−**^ **=** **Q2**^**2−**^
Na^+^ + L^2−^ = [NaL]^−^	2.08(7)	2.40(3)
Mg^2+^ + L^2−^ = [MgL]	5.74(7)	6.93(5)
Ca^2+^ + L^2−^ = [CaL]	7.67(8)	8.94(4)

**Figure 3:**
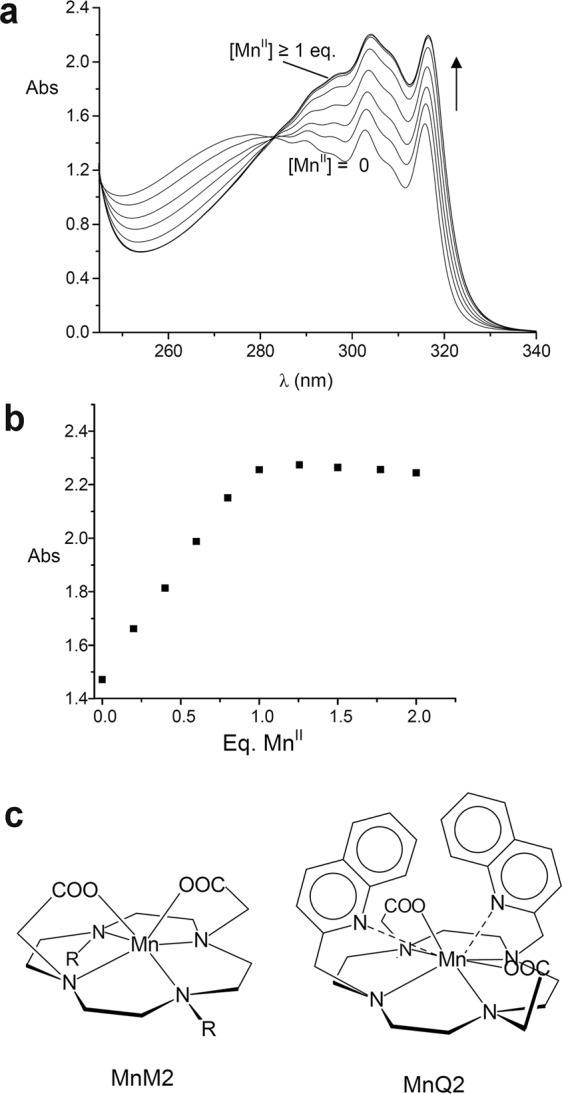
UV-vis spectra of H_2_Q2 (**a**) and absorbance measured at λ 316 nm in the presence of increasing amounts of Mn^II^ (aqueous solution, pH 7.4, [H_2_Q2 = 1.1·10^−4^ M]), and proposed structures of the MnM2 and MnQ2 complexes (in MnQ2 one or both quinoline moieties can be involved in metal coordination: only the case with both the quinoline units interacting with Mn^II^ is represented).

Analysis of the data in Table 1 reveals that Mn^II^ gives stable 1:1 complexes in aqueous solutions with the fully deprotonated species of both ligands. In this respect, the MnQ2 complex is markedly more stable than MnM2, the stability constant of the MnQ2 complex being 4.83 log. units higher than that of MnM2 (log *K* = 19.56(8) *vs* 14.73(2) for the MnM2 and MnQ2 complexes, respectively: Table 1). In the latter, both the carboxylate groups are bound to the metal, a structural feature often found in Mn^II^ complexes with polyamine macrocycles functionalized with acetate pendant arms^17,25–29^. In fact, the amine function and anionic carboxylate groups normally form stable complexes with transition metals, including Mn^II^, and, at the same time, binding to the metals of the N-CH_2_-COO^−^ coordination bite affords a pentadentate chelate ring, which increases the stability of the complexes with first-tow transition metal cations. However, the higher stability observed for MnQ2 would suggest that, besides the carboxylate groups, in MnQ2 the quinoline nitrogen donors could be involved in metal coordination. On the other hand, the heterocyclic nitrogen possesses a lower binding ability than aliphatic amine and carboxylate groups, due to the electron-poor nature of the quinoline^24^. Attempts to obtain crystals of MnQ2 suitable for X-ray single-crystal diffraction analysis failed, thus precluding determination of the coordination sphere of the metal cation in the solid complex. To verify the possible involvement of quinoline in metal coordination we performed UV-vis, ^1^H NMR, e.p.r. (in solution) and IR (in the solid state) measurements on the Mn^II^ complex. UV-vis spectra of H_2_Q2 at pH 7.4 in the presence of increasing amounts of Mn^II^ (Fig. 3a) clearly shows that Mn^2+^ addition to a solution of H_2_Q2 induces an increase of the typical structured absorption band of quinoline between 310 and 320 nm. The absorbance monitored at λ 316 nm (Fig. 3b) linearly increases until a 1:1 metal-to-ligand molar ratio is reached, while the addition of more than 1 eq. of Mn^II^ does not lead to any significant spectral changes. These data confirm the formation of a stable 1:1 complex in aqueous medium, and suggest that one or both quinoline nitrogens could be involved in metal binding (Fig. 3c). The ^1^H NMR spectrum of H_2_Q2 at pH 2 shows a single set of signal in the aromatic region for the quinoline hydrogens (Fig. S15, Supplementary Information 1). In the aliphatic region, the signals are somewhat broaden, making more difficult their attribution. This feature is often found in tetraamine macrocycles, including H_2_M2^30^, and can be related to presence in solution of conformers, which differ in the conformation of the ethylenic chains, with interconversion times of the same order to that of the NMR scale. At pH 7.4, the aromatic region spectrum is composed by two different sub-spectra, which can be attributed to the presence of two different conformers, one of which present in very minor percentage, slowly exchanging on the NMR time scale, while the signals in the aliphatic region are broader. ^1^H NMR signals of Mn^II^ (S = 5/2) compounds are expected to give rise to signals experiencing large contact shift contributions and severe paramagnetic broadening, probably beyond detection. Accordingly, addition of Mn^II^ to a solution of the ligand at pH 7.4 induces progressive line broadening upon increasing Mn^II^ concentration in the spectra recorded at 400 Mhz (Fig. S31, Supplementary Information 1). This phenomenon includes both the aliphatic and the aromatic part of the spectrum, the signal broadening effects being somewhat more evident in the aliphatic region, and leads to the complete disappearance of the less intense subspectrum even in the presence of 0.4 eq. of Mn^II^. These results might be suggestive of the presence of dynamic equilibrium between the Mn^II^ complex and the free ligand. To corroborate this suggestion, the spectra were also recorded at higher field (900 MHz), in order to slow down the exchange regime (Fig. S32, Supplementary Information 1). As expected, the observed line broadening during the titration with Mn^II^ is reduced. In conclusion, the observed spectral changes in the presence of increasing amounts of Mn^II^ support the presence of a complex where not only the tetraamine-dicarboxylate scaffold, but also the quinoline moieties interact with the metal ion.

NMR measurements were also used to analyze Zn^II^ coordination (Fig. S33, Supplementary Information 1). Differently from Mn^II^, the addition of Zn^II^ in solution only induced changes in the aliphatic section of the ^1^H NMR spectrum. Despite the fluxional aspect of the aliphatic signals at pH 7.4, a slight, but not negligible, downfield shift of the CH_2_ signals upon metal coordination could be observed. Conversely, the aromatic part of the spectrum is almost not affected by metal coordination. This titration could not be followed by ^13^C NMR, due to poor solubility of the free ligand at neutral pH (see the Methods section). However, the ^13^C NMR spectrum of ZnQ2 (Fig. S9, Supplementary Information 1) shows two different sets of nine signals for the quinoline carbons in the aromatic region, one of which with weaker signal intensities, in keeping with the presence of two conformers slowly exchanging on the NMR time scale, as already observed in the ^1^H NMR spectra at pH 7.4. Interestingly, the signal of the carboxylate group occurs at 179.79 ppm, as normally found in Zn^II^-bound carboxylate groups^31^. The same signal is shifted at 168.24 ppm in the free ligand at acidic pH values (Fig. S6) and at 164.12 in the case of sodium acetate in water^32^. These results suggest that in the Zn^II^ complex the metal is firmly bound by the carboxylate groups, while quinoline is not involved in the coordination. The different role of quinoline in Mn^II^ and Zn^II^ coordination reflects the ability of the former to expand its coordination environment achieving coordination number greater than six^26,27^. This hypothesis is confirmed by the EPR spectra recorded on the MnM2 and MnQ2 complexes in frozen solution (Fig. S34, Supplementary Information 1). Both spectra are characterized by a signal consisting of six sharp lines, centered at field corresponding at g = 2.00, superimposed on a broader envelope with partially resolved structure (Fig. S34, Supplementary Information 1). The six sharp lines are due to the ^55^Mn-hyperfine splitting associated with the central m_S_ = |−1/2〉 → |+1/2〉 transition, while the structures on the broader features are attributed to transitions between higher m_S_ levels due to the moderate zero field splitting (ZFS) interactions, as expected for Mn^II^. However, the different resonant fields of the broader features for MnM2 and MnQ2 are indicative of different ZFS interactions in the two systems. In particular, the larger extension of the spectrum of MnQ2 lead us to suppose a ZFS larger than for MnM2. These differences in the ZFS interactions can be traced back to a different coordination environment for Mn^II^ in the two complexes. The possible involvement of quinoline in Mn^II^ coordination was also investigated by recording IR spectra on **4**, H_2_Q2 and MnQ2 solid compounds. In particular we paid attention to a typical set of three signals, attributed to C-C and C-N stretching vibrations, easily recognizable in the IR spectra of quinoline compounds between 1530 and 1650 cm^−1^ ^32^. The same set of three signals is also observed in the case of the simple pyridine heterocycle and its shift toward higher wavelength number has been considered diagnostic for coordination of the nitrogen donor to transition metals, including Mn^II^ ^33–35^. In the parent compound **4**, not containing carboxylate units, these bands, indicated with a, b and c in Supplementary Information 1, Fig. S35a, are positioned at 1643, 1603 and 1541 cm^−1^, respectively. In H_2_Q2 two bands attributable to the quinoline ring can be easily recognized at 1599 (b band) and 1539 cm^−1^ (c band), while the a signal originally at 1643 cm^−1^ is superimposed with the C=O stretching at 1626 cm^−1^ (Supplementary Information 1, Fig. S35b). In the MnQ2 spectrum, two bands (a and c in Supplementary Information 1, Fig. S35c) of the quinoline moieties are positioned at higher wavelength numbers (1661 and 1554 cm^−1^, respectively) than in H_2_Q2, while the third band b (at 1589 in H_2_Q2) is superimposed with the C=O stretching signal at 1602 cm^−1^. The observed shift toward higher wavelength number can be indicative of the interaction of the heteroaromatic nitrogen(s) with Mn^II^.

All together, these results suggest that one or both quinoline moieties are involved in metal coordination (Fig. 3c). The observed spectroscopic changes, in particular in the NMR spectra, suggest a weaker interaction of the quinoline nitrogen(s) with respect to that of the amine groups of the macrocycle and the carboxylate groups, in keeping with the lower binding ability of quinoline for metal cations. However, this interaction would justify, at least in part, the higher stability observed for its Mn^II^ complex with respect to MnM2. At the same time, the increased hydrophobic characteristics of H_2_Q2 can also contribute to complex stability. In fact, binding of the metal implies its envelopment in a hydrophobic pocket and hence its complete desolvation, with a stabilizing energetic effect due to the increased translational entropy.

Both the MnM2 and MnQ2 complexes bind acidic protons to give protonated species of the type [Mn(H_2_L)]^2+^ and [Mn(HL)]^+^ in aqueous solution (L = M2 or Q2, see Table 1). In MnM2 complex, both carboxylate groups are coordinated to the metal^17^ and, similarly to most complexes of polyamine-polycarboxylate ligands with transition metals, protonation occurs at the carboxylate groups, leading to their detachment from the metal. In principle, in MnQ2, proton binding can occur at the anionic carboxylate functions or the quinoline nitrogens. However, the protonation constants of MnM2 and MnQ2 complexes are quite similar and, at the same time, methylen-carboxylate groups are more basic than quinolines. These observations suggest that protonation takes place on the carboxylate groups in MnQ2 as well.

**Figure 4:**
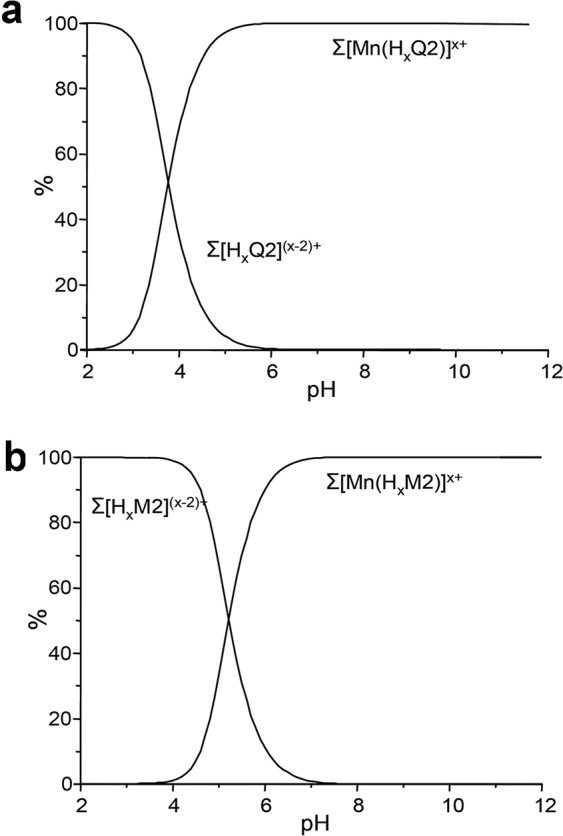
Overall percentages of the H_2_M2 (**a**) and H_2_Q2 (**b**) complexed species with Mn^II^, Ca^II^ Mg^II^ and Na^I^ as a function of pH in competitive systems containing Mn^II^ (1 μM), Ca^II^, (2.5 mM), Mg^II^ (1.5 mM) and Na^I^ (165 mM) and H_2_M2 (1 μM) or H_2_Q2 (1 μM). Σ[Mn(HxQ2)]x+ = [MnQ2] + [Mn(HQ2)]+ + [Mn(H_2_Q2)]2+; Σ[Mn(HxM2)]x+ = [MnM2] + [Mn(HM2)]+ + [Mn(H_2_M2)]2+; [Σ[HxQ2](x − 2)+ = [Q2]2− + [HQ2]− +[H_2_Q2]− + [H_3_Q2]+ + [H_4_Q2]2+ + [H_6_Q2]4+; [Σ[HxM2](x − 2)+ = [M2]2− + [HM2]− + [H_2_M2] +[H_3_M2]+ + [H_4_M2]2+.

Polyamine-polycaboxylate ligands can form complexes even with ‘hard’ metal cations, including K^I^, Na^I^, Ca^II^ and Mg^II^, which are abundant in the cellular and extracellular environments. Therefore, we analyzed the coordination of these metals with H_2_Q2 by potentiometric titrations: the results are reported in Table 1, together with those previously found with H_2_M2^17^. The stability of the K^I^ complexes with both ligands is too low to be confidently determined (log *K* < 2), while Na^I^, Ca^II^ and Mg^II^ formed markedly less stable complexes than Mn^II^. At a lesser extent than Mn^II^, the Na^I^, Ca^II^ and Mg^II^ complexes with H_2_Q2 are somewhat more stable than those with H_2_M2. Interestingly, the difference in stability between H_2_Q2 and H_2_M2 complexes with these metals is remarkably lower than that observed for Mn^II^, reflecting the poor affinity of quinoline toward alkali and alkaline earth metal cations. The observed higher stability of the H_2_Q2 complexes with Na^I^, Ca^II^ and Mg^II^ is likely due to a stronger desolvation of these metal cations upon binding to the hydrophobic scaffold rather than to the interaction with the heteroaromatic nitrogens of quinolines. As a result, H_2_Q2 is more selective for Mn^II^ over Na^I^, Ca^II^ and Mg^II^ than H_2_M2. To assess whether Na^I^, Ca^II^ and Mg^II^ could compete with Mn^II^ in the complexation process, we considered competitive systems containing each ligand and Mn^II^ at very low concentrations (1 μM) in the presence of excess of Na^I^ (165 mM), Ca^II^ (2.5 mM) and Mg^II^ (1.2 mM), roughly reproducing the cellular concentrations of these metals. We then calculated the overall percentages of the different metal cations complexed with H_2_Q2 over a wide pH range^36,37^, which are shown in the competition diagram in Fig. 4a. The diagram obtained for H_2_M2 is also reported (Fig. 4b). Mn^II^ remained completely bound to the two scaffolds while Ca^II^, Mg^II^ and Na^I^ appeared unbound. This finding indicates that MnM2 and MnQ2 are substantially unaffected by other metal ion competitors in the medium and no detectable Mn^II^ release occurs due to displacement by other metals. Mn^II^ release occurs in low percentage (less than 10%) only below pH 4.8 and 6 in the case of H_2_Q2 and H_2_M2, respectively, due to extensive protonation of the ligands at acidic pH values. H_2_Q2 is more resistant to dissociation in acidic media than H_2_M2, a relevant characteristic if taking into account that some functional cell compartments, such as the mitochondrial outer chamber, have acidic features.

The stability of MnM2 and MnQ2 was also higher than other Mn^II^ complexes with endogenous ligands present in the cellular environment, such as ATP (log *K* = 4.66 for the equilibrium Mn^II^ + ATP = [Mn^II^(ATP)]), glutathione (log *K* = 2.7 for Mn^II^ + GSH = [Mn^II^(GSH)]) and carboxylate anions (log *K* = 3.79, 1.68 and 0.92 for Mn^II^ complexation by citrate, maleate and L-lactate anions)^24^, thus excluding substantial de-metallation of these scaffolds by biological chelators.

We also determined the stability of the complexes formed by H_2_M2 and H_2_Q2 with Zn^II^. Like Mn^II^, Zn^II^ is a bivalent first-row transition metal cation sharing several chemical features with Mn^II^. However, Zn^II^ ion is extremely stable to reduction, while oxidation states higher than II are not accessible. These characteristics make ZnM2 and ZnQ2 inert to reducing and oxidizing agents in the cellular environment, including ${{\rm{O}}}_{2}^{\cdot -}$ or other ROS. Both scaffolds display binding ability for Zn^II^, similar (H_2_M2) or slightly lower (H_2_Q2) than that observed for Mn^II^ (log *K* = 14.23(5) and 18.5(1) for the equilibrium L^2−^ + Zn^2+^ = [ZnL], with L = M2 and Q2, respectively), the metal centre being likely coordinated by the tetramine scaffold and the two carboxylate groups.

### *In vitro* antioxidant properties of MnQ2 and MnM2

**Figure 5:**
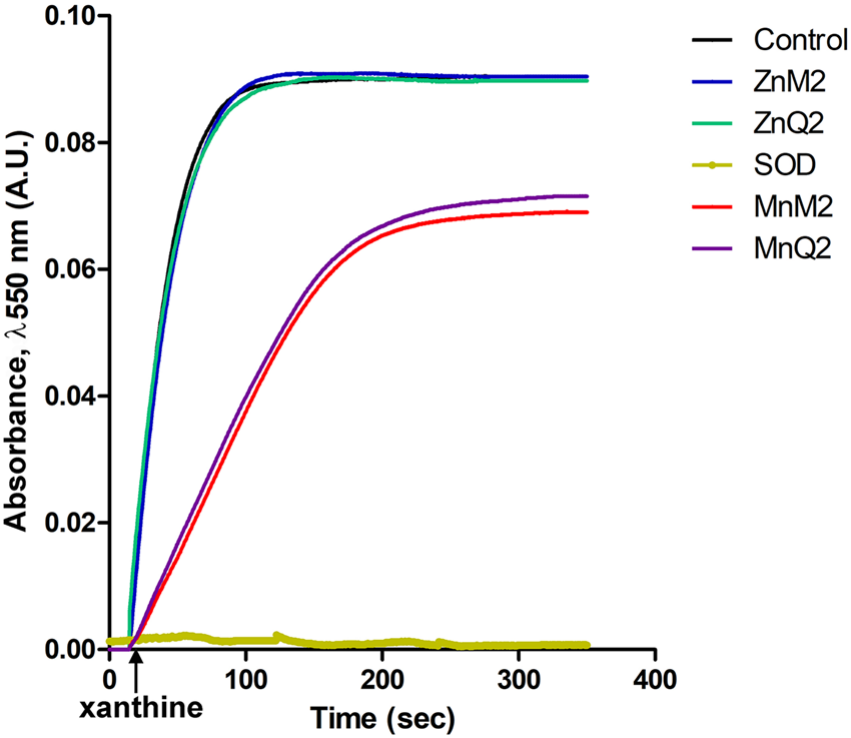
Kinetic of ${{\rm{O}}}_{2}^{\cdot -}$ decomposition by MnQ2 and MnM2 assayed spectrophotometrically (λ 550 nm) by inhibition of cytochrome *c* reduction. In the control experiment (black line), xanthine was converted to uric acid in about 150 s (max. absorbance: 0.09; *v*_max_: 0.0021 arbitrary units, AU, s^−1^). The cytochrome *c* reduction kinetic changed substantially in the presence of 50 μM MnM2 (red line) (max. absorbance: 0.069; *v*_max_: 0.000457 AU s^−1^) or 50 μM MnQ2 (purple line) (max absorbance: 0.072; *v*_max_: 0.000460 AU s^−1^), with a substantially similar catalytic efficiency of MnQ2 and MnM2. ZnM2 (blue line) and ZnQ2 (green line), in which the functional Mn^II^ was replaced with redox-inert Zn^II^, did not induce any significant differences in respect to the control.

The ${{\rm{O}}}_{2}^{\cdot -}$-scavenging ability of MnQ2 and MnM2 was monitored spectrophotometrically at 550 nm by the inhibition of cytochrome *c* reduction at pH 6.8 and 37 °C in the presence of catalase. As shown in Fig. 5, in the control experiment, approximately all xanthine was converted to uric acid in about 150 s, (max. absorbance: 0.09; *v*_max_: 0.0021 arbitrary units, AU, s^−1^). The cytochrome *c* reduction kinetic changed substantially in the presence of 50 μM MnM2, (max. absorbance: 0.069; *v*_max_: 0.000457 AU s^−1^) or 50 μM MnQ2 (max absorbance: 0.072; *v*_max_: 0.000460 AU s^−1^). These values demonstrate a similar catalytic efficiency of MnQ2 and MnM2. The presence of ZnM2 (blue line) or ZnQ2 (green line), in which the functional Mn^II^ was replaced with redox-inert Zn^II^, did not induce any significant differences in respect to the control.

The rate constants for the reaction of MnQ2 and MnM2 with ${{\rm{O}}}_{2}^{\cdot -}$ were then determined by competition kinetics using cytochrome *c* oxidation as revealing system: MnQ2 *k*_*cat*_ = 2.5 × 10^6^ M^−1^ s^−1^; MnM2 *k*_*cat*_ = 4.5 × 10^6^ M^−1^ s^−1^. The experimental details are reported in Supplementary Information 2.

### Cell permeation properties of MnQ2 and MnM2

**Figure 6:**
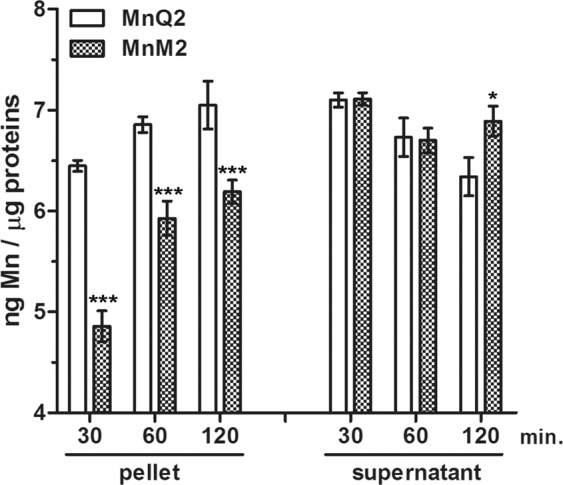
Time-related permeation of MnQ2 and MnM2 into H9c2 cell fractions evaluated as intracellular Mn amounts. The pellet fraction contains mitochondria whereas the supernatant fraction corresponds to cytosol. Upon incubation with MnQ2 (10 μmol L^−1^), Mn rises steadily in both cell fractions since 30 min. Upon incubation with MnM2 (10 μmol L^−1^), Mn also rises steadily in the cytosolic fraction but shows a time-related increase in the mitochondrial fraction. In this fraction, Mn was significantly higher with MnQ2 than with MnM2 at every time point. ***p < 0.001, *p < 0.05 (two-way ANOVA).

Time-course analysis of the levels of Mn in the mitochondrial (pellet) and cytosolic (supernatant) cellular fractions, assumed as indicator of cell permeation capability, showed that both MnQ2 and MnM2 were able to penetrate into H9c2 cells but with different kinetics. Upon incubation with MnQ2, intracellular Mn appeared to steadily rise within both cell fractions since 30 min. On the other hand, MnM2 yielded Mn concentrations similar to MnQ2 in the cytosolic fraction, while it induced a time-related increase in Mn in the mitochondrial fraction. In this fraction, intracellular Mn was significantly higher with MnQ2 than with MnM2 at every time point (Fig. 6). The baseline values of Mn in the untreated control cells were 2.44 and 2 ng μg^−1^ of proteins in the pellet and supernatant fractions, respectively.

### Protection from hypoxia-reoxygenation (H + R)-induced cellular oxidative stress by MnQ2 and MnM2

**Figure 7:**
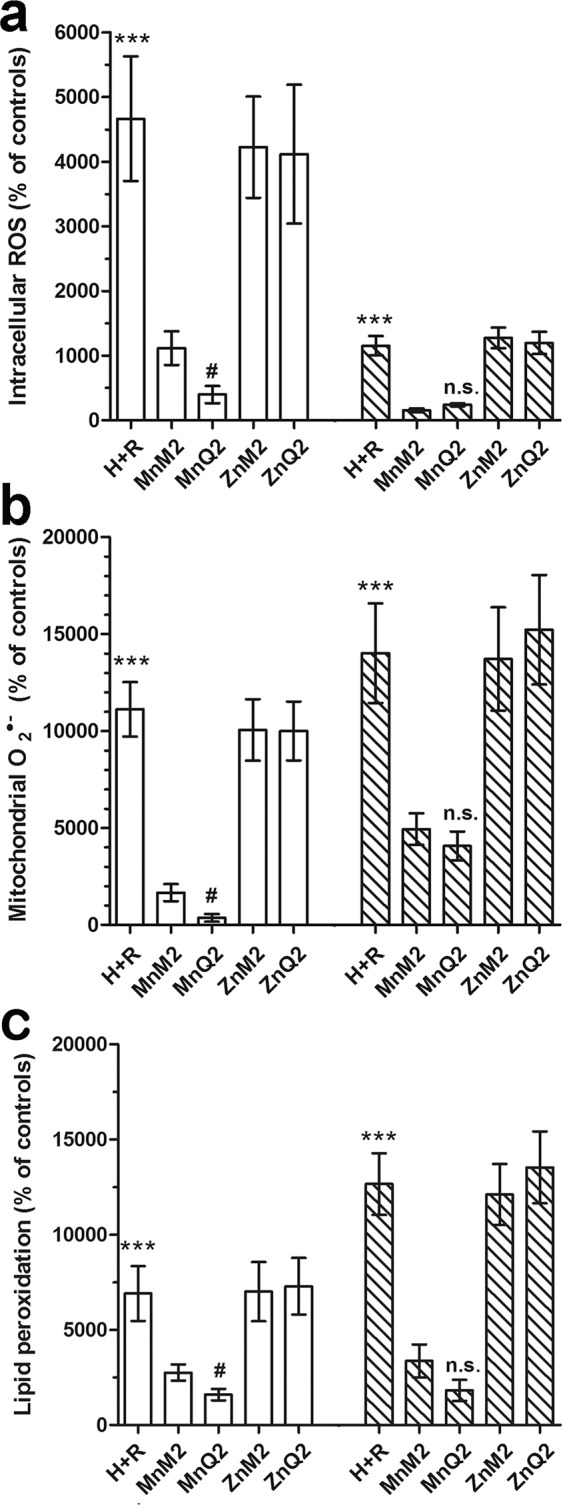
FACS analysis of intracellular ROS by H_2_DCFDA (**a**), of mitochondrial ${{\rm{O}}}_{2}^{\cdot -}$ production by MitoSOX (**b**) and of cellular lipid peroxidation by BODIPY-581/591-C_11_, evaluated at 1 h (open columns) and 2 h (striped columns) and expressed as percent changes of the untreated controls. All these parameters were enhanced upon H + R and significantly reduced by MnQ2 and MnM2. At the shorter time point (1 h), MnQ2 was significantly more effective than MnM2. ***p < 0.001 vs. controls, MnM2 and MnQ2; (one-way ANOVA); ^#^p < 0.05, n.s. not significant vs. MnM2 (Student’s *t* test).

Both MnQ2 and MnM2, added at reoxygenation (10 μmol/L), afforded protection to H9c2 cells by reducing the oxidative stress occurring upon H + R, but with different timing. The levels of endogenously generated ROS, determined by loading the cells with the intracellular fluorescent probe 2′,7′-dichlorodihydrofluorescein diacetate (H_2_DCFDA), were significantly lower in the cells treated with either MnQ2 or MnM2 than in the untreated control cells: of note, MnQ2 was significantly (*p* < 0.05) more potent than MnM2 in the short term (1 h), while the two scavengers showed similar effects at the longest time point (2 h) (Fig. 7a).

Similar results were obtained from the evaluation of mitochondrial ${{\rm{O}}}_{2}^{\cdot -}$ generation by the fluorescent probe MitoSOX. Both MnQ2 and MnM2 significantly reduced ${{\rm{O}}}_{2}^{\cdot -}$-dependent fluorescence in comparison with the untreated controls: again, MnQ2 showed a significantly higher effect than MnM2 in the short term (1 h) (Fig. 7b).

In keeping with these findings, the degree of cell lipid peroxidation, a by-product of excess ROS, assessed by the red-to-green fluorescence shift of BODIPY-581/591-C_11_ lysochrome, was markedly enhanced upon H + R and significantly reduced by MnQ2 and MnM2 added at reoxygenation, the former compound being slightly more active than the latter one in the short term (1 h) (Fig. 6c).

In all the above experiments, ZnQ2 and ZnM2 added in the place of their Mn-containing counterparts had no antioxidant effects (Fig. 7). Representative confocal images and fluorescence-activated cell sorting (FACS) analysis of these experiments at the 1-h time point are shown in Fig. 10 and Supplementary Information 3, respectively.

### Preservation of impaired cell mitochondrial activity by MnQ2 and MnM2

**Figure 8:**
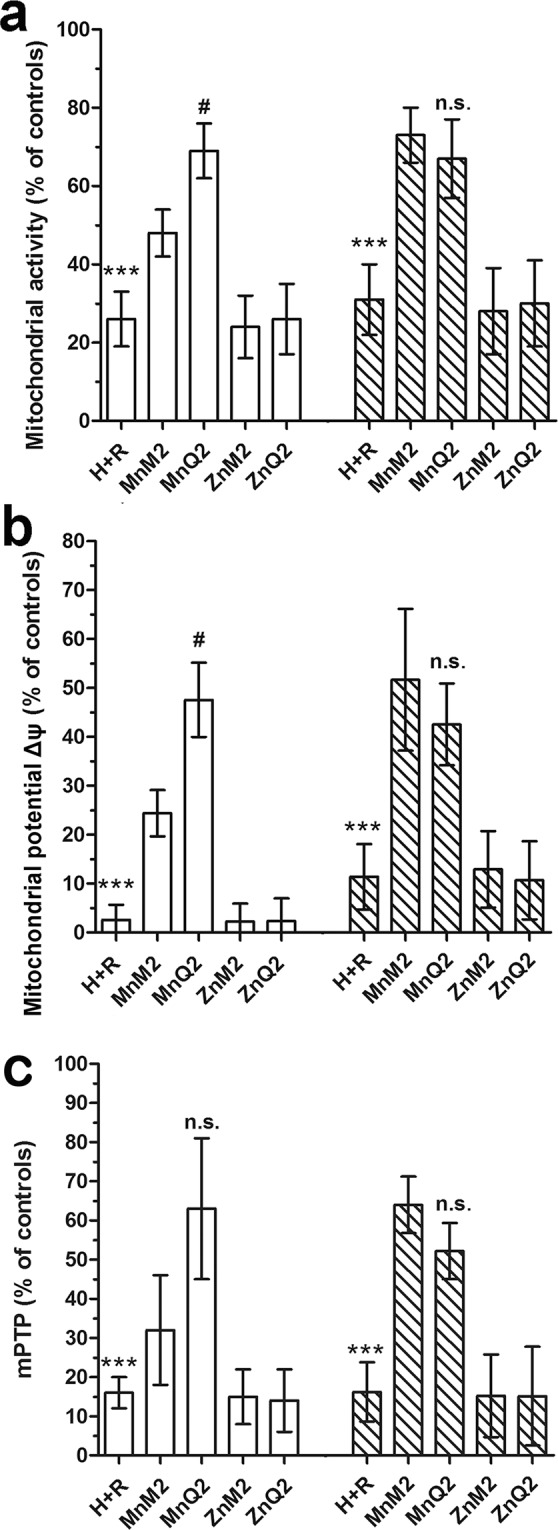
FACS analysis of mitochondrial activity by RSZ (**a**), of mitochondrial membrane potential (Δψ) by TMRM (**b**) and of mitochondrial transition pore opening (mPTP) by extinction of calcein fluorescence, evaluated at 1 h (open columns) and 2 h (striped columns) and expressed as percent changes of the untreated controls. All these parameters were decreased by H + R and significantly increased by MnM2 and MnQ2. At the shorter time point (1 h), MnQ2 was more effective than MnM2. ***p < 0.001 vs. controls, MnM2 and MnQ2; (one-way ANOVA); ^#^p < 0.05, n.s. not significant vs. MnM2 (Student’s *t* test).

Both MnQ2 and MnM2, added at reoxygenation (10 μmol/L), improved the assayed markers of mitochondrial activity which were compromised by H + R-induced oxidative stress, albeit with different timing. In detail, the efficiency of the respiratory chain, measured as fluorescence of reduced resazurin (RSZ) dye (Fig. 8a) and the mitochondrial membrane potential (Δψ), evaluated through the inlet of the fluorochrome tetramethylrhodamine methyl ester perchlorate (TMRM) (Fig. 8b), increased significantly in H9c2 cells treated with MnQ2 and MnM2 in comparison with the untreated controls. Conversely, opening of transition pores (mPTP), an index of mitochondrial dysfunction and early apoptosis measured through the extinction of calcein fluorescence, decreased significantly upon addition of MnQ2 and MnM2 (Fig. 8c). Similarly to what observed in the above experiments on oxidative stress, MnQ2 was more effective than MnM2 in preserving mitochondrial function in the short term (1 h), while at the longest time (2 h) the two compounds showed similar potency. Replacement of the redox-active compounds with ZnQ2 and ZnM2 resulted in loss of protection against H + R-induced mitochondrial dysfunction (Fig. 8). Representative confocal images and FACS analysis of these experiments at the 1-h time point are shown in Fig. 10 and Supplementary Information 3, respectively.

### Protection from H + R-induced cell death by MnQ2 and MnM2

**Figure 9:**
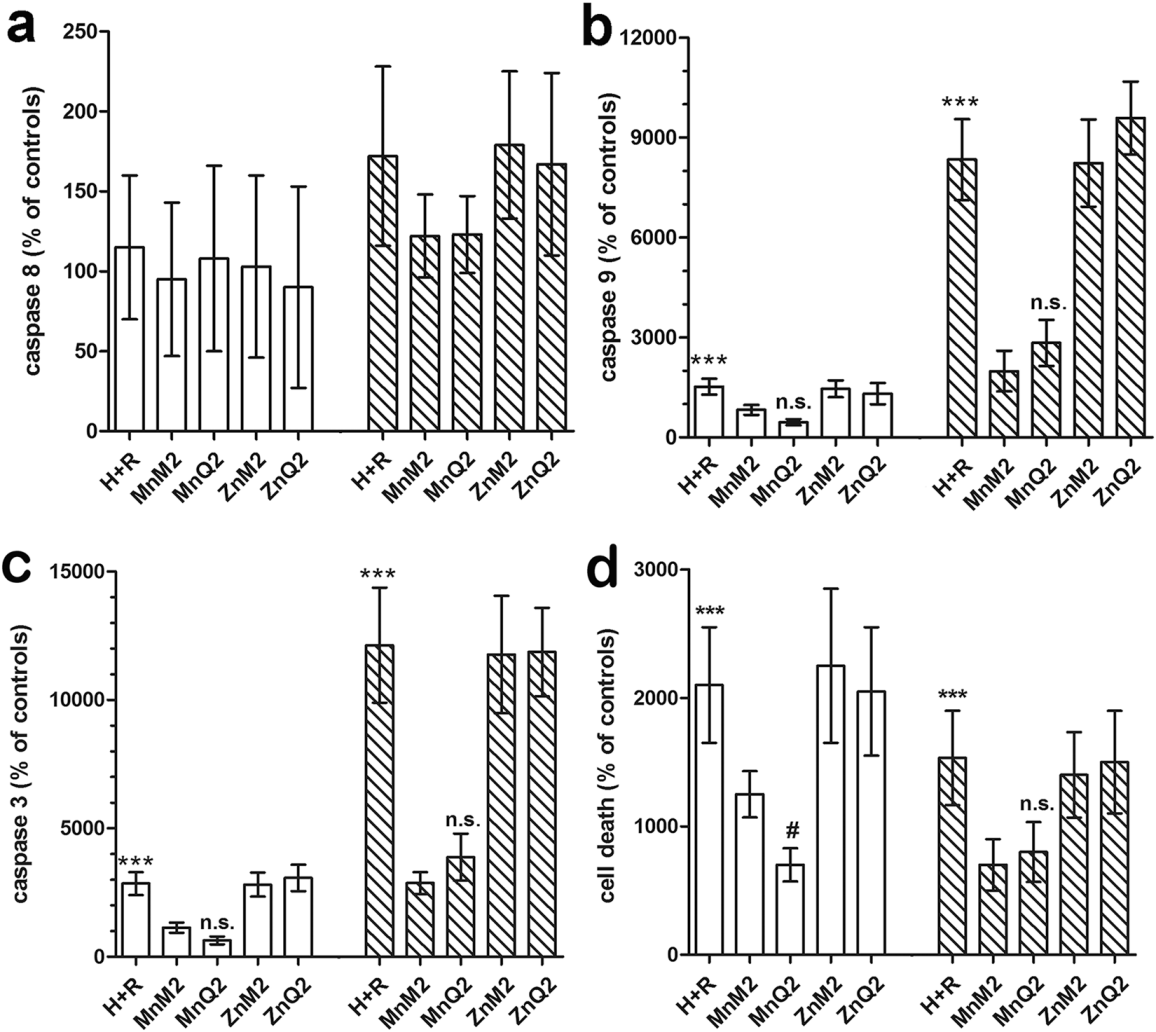
FACS analysis of fluorescent reporter activity of apoptosis initiator caspases 8 (**a**) and 9 (**b**), of apoptosis effector caspase 3 (**c**) and of cell death assayed by LDH release (**d**), evaluated at 1 h (open columns) and 2 h (striped columns) and expressed as percent changes of the untreated controls. Caspase 8, involved in the extrinsic apoptotic pathway, was unaffected by any treatment and exposure times. Conversely, caspase 9, involved in the intrinsic apoptotic pathway, was increased by H + R and significantly decreased by MnQ2 and MnM2. Caspase 3 and overall cell death were increased by H + R and significantly decreased by MnQ2 and MnM2. At the shorter time point (1 h), MnQ2 was significantly more effective than MnM2 to reduce cell death. ***p < 0.001 vs. controls, MnM2 and MnQ2; (one-way ANOVA); ^#^p < 0.05, n.s. not significant vs. MnM2 (Student’s *t* test).

**Figure 10:**
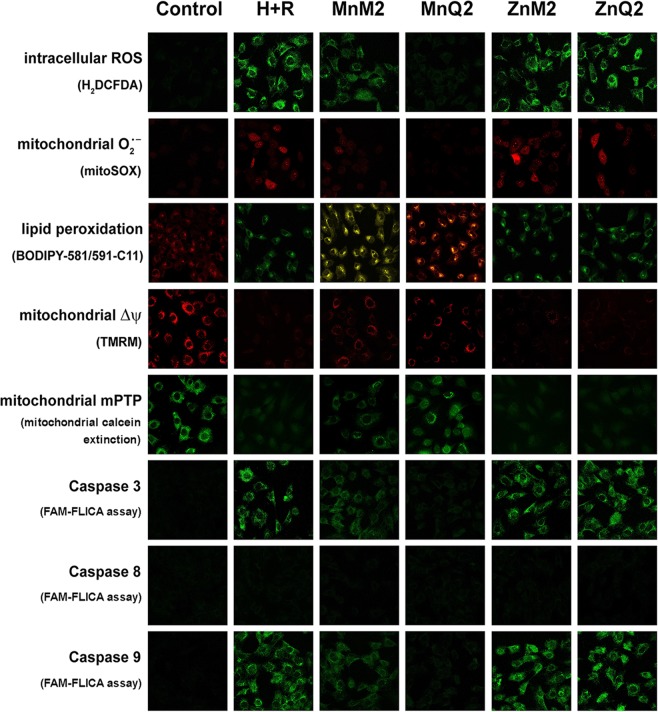
Representative confocal micrographs of H9c2 cells at the noted experimental conditions, examined after 1 h reoxygenation. Magnification, ×500.

Since mitochondrial impairment triggers the intrinsic pathway of apoptosis operated by the caspase 9 – caspase 3 cascade^38^, we next investigated whether the protection afforded by MnQ2 and MnM2 on mitochondrial oxidative dysfunction reflected in improved survival of H9c2 cells subjected to H + R. Compared with the untreated controls, the cells added with MnQ2 and MnM2 at reoxygenation showed a decrease in pro-apoptotic activation of caspases 9 and 3, whereas caspase 8, involved in the extrinsic apoptotic pathway, was substantially unchanged (Fig. 9a–c). Consistent with these findings, evaluation of the percentage of dead cells in the cultures showed a significant rise in the untreated controls, which was blunted by MnQ2 and MnM2 (Fig. 9d). Again, MnQ2 was more effective than MnM2 to preserve H9c2 cell viability in the short term (1 h). ZnQ2 and ZnM2 substituted for the redox-active compounds showed no cytoprotective effects (Fig. 9). Representative confocal images and FACS analysis of these experiments at the 1-h time point are shown in Fig. 10 and Supplementary Information 3, respectively.

### Modulation of MAPK activation by MnQ2 and MnM2

**Figure 11:**
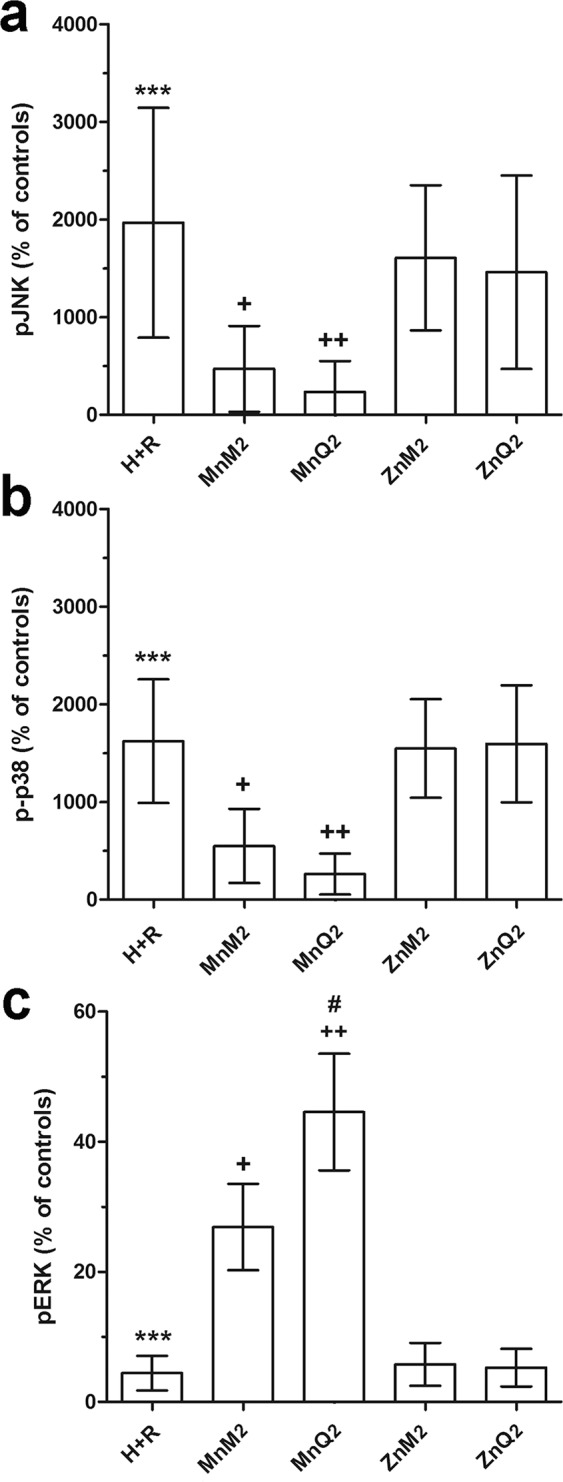
FACS analysis of immunofluorescent expression of phosphorylated (p−) JNK (**a**) p38 (**b**) and ERK1/2 (**c**), evaluated at 1 h and expressed as percent changes of the untreated controls. The oxidative stress-related MAPKs p-JNK and p-p38 were increased by H + R and significantly decreased by MnQ2 and MnM2. Conversely, the cytoprotective pERK was decreased by H + R and significantly increased by MnQ2 and MnM2, the former compound being significantly more effective than MnM2. ***p < 0.001 vs. controls; ^+^p < 0.05, ^++^p < 0.01 vs. H + R; (one-way ANOVA); ^#^p < 0.05 vs. MnM2 (Student’s *t* test).

Among MAPKs, extracellular signal-regulated kinases (ERK) p38 and JNK are activated upon harmful stimuli, including oxidative stress^39^. In the present experiment, H + R induced a significant increase in p38 and JNK phosphorylation, an index of enzyme activation, while this phenomenon was reduced by 1-h incubation with MnQ2 and, at a lesser extent, MnM2 (Fig. 11). On the other hand, phosphorylation/activation of ERK1/2, which can operate cytoprotective and anti-apoptotic mechanisms in response to oxidative stress^39,40^, was impaired by H + R and up-regulated by 1-h incubation with MnQ2 and MnM2. In both instances, ZnQ2 and ZnM2 were ineffective.

## Discussion

The present study indicates that the pharmacokinetic and pharmacological characteristics, in terms of intracellular permeation and consequent ${{\rm{O}}}_{2}^{\cdot -}$-scavenging and anti-oxidant efficacy of the studied redox-active Mn^II^ complexes can be improved by appropriate modulation of the lipophilic and structural characteristics of their polyamine-polycarboxylate scaffolds. For that purpose, we have synthesized the new compound MnQ2, containing two quinoline units replacing two methyl groups in position 4, 10 of the cyclic tetraamine scaffold of MnM2. This structural modification was achieved by the bis-aminal synthetic procedure, which allowed the attachment of different functional moieties on selected nitrogen atoms within a tetraamine scaffold. Of note, the strategy to increase lipophilicity by targeted chemical substitutions has also been adopted to improve the bioavailability of Mn-porphyrins, another class of redox-active drugs^16^. In both MnM2 and MnQ2 simultaneous binding of Mn^II^ to the tetraamine macrocycle and to the 2 carboxylate appendices makes them thermodynamically stable and highly resistant to Mn^II^ release. At physiological pH, Mn^II^ forms a highly stable complex with the polyamine-polycarboxylate scaffold, markedly more stable than that with other metal cations present in the cellular environment, such as Ca^II^, Mg^II^ and Na^I^. Moreover, the neutral charge of these compounds facilitates trans-membrane passage and intracellular localization. The presence of two quinoline or methyl groups appended to amine groups in 4, 10 position prevents the nitrogen atoms to interact with water molecules through hydrogen bonds. The insertion of two quinoline units not only increases the hydrophobic features of the resulting polyamine ligand, but also markedly enhances the stability of the MnQ2 complex. (log *K* = 19.56(8) and 14.73(2) for the equilibrium Mn^2+^ + L^2−^ = [MnL], with L = Q2 and M2, respectively). In fact, in MnQ2 the heteroaromatic nitrogens of quinoline can also participate in Mn^II^ coordination, reinforcing the overall metal-to-ligand interaction and increasing complex stability. Although the present results are not sufficient to discern whether one or both quinoline nitrogen are involved in Mn^II^ coordination, the metal ion is coordinated not only by the tetraamine macrocycle and the carboxylate groups, as in the case of the MnM2 complex, but also by at least one quinoline nitrogen, in agreement with the ability of Mn^II^ to achieve coordination number greater than 6. In the meantime, the two quinolines increase the lipophilic features of the complex and, overall, create a hydrophobic environment for the metal cation, leading to enhanced desolvation of the metal and increased translational entropy, further stabilizing the Mn^II^ complex. Conversely, the scarce binding affinity of the quinoline nitrogen for alkali and alkaline-earths leads to a binding ability for these metal cations that was only slightly higher than that of the H_2_M2 ligand. As a result, H_2_Q2 shows a higher selectivity for Mn^II^ than for other metal ions largely present in the cellular environment, such as K^I^, Ma^I^, Ca^II^ and Mg^II^. These characteristics prevent both trans-metallation reactions, due to complexation of the ligand to other metals in the cell, and de-metallation due to Mn^II^ complexation by cellular chelating agents. In fact, MnQ2 and MnM2 did not release Mn^II^ even in the presence of large excess of Ca^II^ and other metal ions, or broad variations of pH. Both Mn^II^ complexes are stable in the alkaline region and at neutral pH. Metal release is observed at acidic pH values, where extensive ligand protonation compete with metal complexation. Mn^II^ release occurs only below pH 6 for MnM2 and pH 5 for the more stable MnQ2 (in the latter case the release of Mn^II^ is about 10% at pH 4.8 and 50% at pH 3.8). Both complexes are completely dissociated at more acidic pH (below pH 4 for MnM2 and pH 3 for MnQ2). This characteristic can be important in light of the fact that in active mitochondria the outer chamber has acidic features. Of note, the absence of negative charge and increased lipophilicity have been shown to facilitate accumulation of Mn complexes in mitochondria^15,22^. These reports and the present findings suggest that MnQ2 and, at a lesser extent, MnM2 can readily enter the cells and directly scavenge ${{\rm{O}}}_{2}^{\cdot -}$ at its mitochondrial generation sites.

The molecular mechanism of ${{\rm{O}}}_{2}^{\cdot -}$ scavenging of MnQ2 and MnM2 likely consists in a catalytic cycle involving: (i) oxidation of Mn^II^ to Mn^III^ by ${{\rm{O}}}_{2}^{\cdot -}$, (ii) reduction of Mn^III^ complex by another ${{\rm{O}}}_{2}^{\cdot -}$ to form the initial Mn^II^ compound^17,20^. To confirm these assumptions, the ${{\rm{O}}}_{2}^{\cdot -}$-scavenging effects of MnQ2 and MnM2 were completely lost when redox-inert Zn^II^ was substituted for Mn^II^. As shown by time-course inhibition of cytochrome *c* reduction, the ${{\rm{O}}}_{2}^{\cdot -}$-scavenging ability of MnQ2 was lower than MnM2 (*v*_max_: 0.000460 *vs*. 0.000457 absorbance arbitrary units s^−1^) and *k*_*cat*_ evaluation (2.5 vs. 4.5 × 10^6^ M^−1^ s^−1^). This is likely due to the steric hindrance that quinolines impose towards the approach of ${{\rm{O}}}_{2}^{\cdot -}$ to the Mn center. On the other hand, in a cellular model, this limitation of MnQ2 appears to be well balanced by its increased lipophilicity which improves biodistribution.

There is a general trend of pharmaceutical research towards the tuning-up of existing medicinal ingredients, by targeted chemical modifications or proper formulations, in order to improve their pharmacokinetic properties, selectivity of action and overall efficacy. Concerning anti-oxidant drugs, their actual efficacy largely depends on the ability to reach the intracellular sites of ROS generation, mainly mitochondria, and keep them under the toxicity threshold, mimicking and reinforcing the natural anti-oxidant systems^41^. The present cell culture system is a good model to study and compare the anti-oxidant properties of MnQ2 and MnM2, since the mechanism of cellular damage induced by H + R involves primarily a dysregulated mitochondrial generation of ${{\rm{O}}}_{2}^{\cdot -}$ ^42,43^, similarly to aging^5–7^. Under the reported experimental conditions, the redox-active compounds MnQ2 and MnM2, added at reoxygenation at micromolar concentrations, effectively abated intracellular ROS and mitochondrial ${{\rm{O}}}_{2}^{\cdot -}$ generation, reduced mitochondrial dysfunction and activation of the caspases of the intrinsic apoptotic pathway, and increased cell viability. Of note, the more lipophilic the molecule, the faster its effects: MnQ2 was able to afford significant protection 1 h after administration to the cells. During prolonged incubation time (2 h), the anti-oxidant efficacy of both MnQ2 and MnM2 became similar, along with the progressive increase in MnM2 intracellular levels approaching those of MnQ2. In keeping with these findings, previous studies have shown that lipophilicity of different Mn-containing compounds can be a good predictor of their ability to enter the cells^44^.

The present findings on MAPK phosphorylation are consistent with activation of an intracellular response to oxidative stress. As expected on the basis of on their antioxidant properties, MnQ2 and MnM2 (1 h) were able to reduce p38 and JNK phosphorylation increased by H + R. Conversely, both compounds significantly increased phosphorylation of cytoprotective, anti-apoptotic ERK1/2. This suggests that MnQ2 and MnM2, besides ${{\rm{O}}}_{2}^{\cdot -}$ scavenging, could also activate this intrinsic cell defense mechanism. These findings fit well with a previous study showing similar effects of a Mn-porphyrin, belonging to a similar class of redox-active drugs, on mammary carcinoma cells subjected to radiation-induced oxidative stress^45^.

Modulation by phosphorylation of key cellular pathways involving transcription factors, such as Nrf2 and NF-*k*B, has recently emerged as a parallel mechanism of action of Mn-porphyrins, adjunctive to or even prevailing on canonical ROS scavenging. In particular, Mn-porphyrins have been shown to exhibit protective effects by upregulation of Nrf2 and inhibition of NF-*k*B and MAPK^15,16,45^. Nrf2 is known to upregulate endogenous antioxidant enzymes, including MnSOD, catalase, peroxiredoxins, glutathione peroxidase, etc., which in turn can reduce the levels of ${{\rm{O}}}_{2}^{\cdot -}$ and H_2_O_2_. On the other hand, inhibition of NF-*k*B results in suppression of NADPH oxidases and reduction of RS levels^15,16^. Whether MnQ2 and MnM2, similarly to Mn-porphyrins, may modulate Nrf2 and NF-*k*B to exert their antioxidant cytoprotective effects cannot be inferred by the present data. Of note, MnQ2 and MnM2 contain Mn^II^ while Mn-porphyrins contain Mn^III^, and it cannot be ruled out that the above properties may be related, at least in part, to the oxidation state of the Mn center. However, this is a tempting working hypothesis for further studies aimed at clarifying the spectrum of biological effects of these new redox-active compounds.

In conclusion, this study indicates that the insertion of hydrophobic groups with metal-coordinating ability within a polyamine-polycarboxylate scaffold can improve its pharmacological properties. Both MnQ2 and MnM2 behave as efficient ${{\rm{O}}}_{2}^{\cdot -}$ scavengers and may represent a promising new class of redox-active drugs. Of note, in respect to MnM2, MnQ2 has a greater tendency to readily enter the cells owing to its enhanced lipophilic features, is less susceptible to transmetallation reactions and is more resistant to Mn^II^ decomplexation at acidic pH values, as those occurring in the mitochondrial compartment. These characteristics make MnQ2 able to efficiently reduce oxidative cell injury mediated by mitochondrial generation of ${{\rm{O}}}_{2}^{\cdot -}$, a key pathogenic event of ischemia-reperfusion damage^42,43^ and aging^5–7^. These findings can improve the understanding of the structure-activity relationships of Mn^II^-polyamine-polycarboxylates as redox-active drugs. In perspective of possible drug development, the chemical features of MnQ2 and MnM2 suggest that these molecules can withstand inactivation by oxidative stress conditions, at variance with synthetic or extractive SOD^46^, whose pharmaceutical use is also limited by poor stability in water and intracellular penetration, short half-life and immunogenicity concerns^47,48^. Further investigations on organ and animal models of endogenous oxidative stress are required to validate the actual therapeutic potential of MnQ2 and MnM2. In this context, transplant medicine may represent a promising field: hypothetically, these compounds could be added to the incubation fluid of explanted organs to extend their viability and reduce ischemia-reperfusion injury upon re-implantation. Of note, suitability of Mn-based redox-active molecules as a promising new class of anti-oxidant drugs is indicated by the fact that the Mn-containing compounds BMX-001 and GC4419 (formerly M40403) are being tested in clinical trials to reduce the adverse effects of radiotherapy-induced oxidative stress in cancer patients^49^.

## Methods

### Reagents

MnM2 was synthesized in our laboratory from 4,10-dimethyl-1,4,7,10-tetraazacyclododecane-1,7-diacetate, as previously described^17^, on kind permission of the patent owner (General Project Ltd., Montespertoli, Italy). The aqueous solutions used in complex synthesis and in potentiometric measurements were deoxygenated by bubbling N_2_ to prevent possible oxidation of Mn^II^. All new compounds were characterized by elemental analysis and mass spectroscopy. Elemental analyses (C, H, N content) were performed with Perkin-Elmer 2400 CHN elemental analyzer. C, H, N and Mn percentages were within ±0.2% of theoretical value, in keeping with a >95% purity for all compounds). Determination of the Mn content in the complexes was performed using a Varian 720-ES inductively coupled plasma atomic emission spectrometer (ICP-AES). The biological samples were treated as previously reported^17^ to obtain suitable solutions for the ICP-AES determination of Mn^II^ content. IR spectra were collected by a IRAffinity-1S Shimadzu instrument. UV-vis spectra were recorded on a Perkin-Elmer lambda 25 spectrometer.

### Synthesis of H_2_Q2 and its Mn^II^ and Zn^II^ complexes 4a-8a-bis(methylen-2-quilolyn)dodecahydro-2a,4a,6a,8a-tetraazacyclopenta[fg]acenaphthylene-di chloride salt (**3**·2Cl)

A solution of 2.58 g (14.5 mmol) of 2-(chloromethyl) quinoline (**2**) in CH_3_CN (15 mL) was added dropwise to a stirred solution of decahydro-2a,4a,6a,8a-tetraaza-cyclopenta[fg]acenaphthylene **1** (1.13 g, 5.80 mmol) in 15 mL of CH_3_CN at room temperature. The solution was kept under stirring at room temperature for 10 days under a nitrogen atmosphere. Product **3**·2Cl precipitated as a light-yellow powder, which was filtered off and washed with CH_3_CN. Yield 1.47 g (46%) Anal. calcd. for C_30_H_34_N_6_Cl_2_: C 65.57, N 15.29, H 6.24; found C 65.12, N 15.09, H 6.36; ^1^H-NMR (400 MHz, MeOD): δ(ppm) 8.52 (d, 2H), 8.15 (d, 2H), 8.04 (d, 2H), 7.86 (t, 2H), 7.81 (d, 2H), 7.72 (t, 2H), 5.41 (d, 2H), 5.24 (d, 2H); 5.15 (s, 2H); 4.61 (m, 2H), 4.13 (m, 6H), 3.65 (m, 2H), 3.47 (m, 4H), 3.19 (m, 2H); ^13^C-NMR (400 MHz, MeOD): δ(ppm) 150.37, 149.05, 139.61, 131.87, 130.39, 129.35, 129.24, 129.14, 123.97, 80.04, 64.09, 62.06, 57.64, 44.23. ESI MS (m/Z) 239.1423 (**3**^2+^), 336.2186 (**3**^**+**^).

### 1,7-bis(methylen-2-quinolyl)-1,4,7,10-tetraazacyclododecane three-hydrobromide salt (**4**)

1.28 g (2.33 mmol) of **3**·2Cl were dissolved in 25 mL of N_2_H_4_ and 5 mL of ethanol and the mixture was stirred at 110 °C for 6 hours. After cooling at room temperature, the solvent was evaporated under reduced pressure affording a yellow solid deposit, which was dissolved in NaOH 15 M aqueous solution (10 mL). The resulting solution was extracted with chloroform (4 × 25 mL). The organic layers were collected, dried with Na_2_SO_4_ and the solvent was finally removed under vacuum. The crude product was dissolved in ethanol (20 mL). Then, 48% HBr (1 mL) was added dropwise to the resulting solution, affording the three-hydrobromide salt of **4** (**4**·3HBr·2H_2_O as a yellow solid. Yield 1.33 g (78%) Anal. calcd. for C_28_H_41_N_6_O_2_Br_3_: C 45.86, N 11.46, H 5.63; found C 46.0, N 11.40, H 5.55; ^1^H-NMR (400 MHz, D_2_O, pD = 2): δ 8.86 (d, 2H), δ 8.17 (d, 2H), δ 8.14(d, 2H), δ 8.02 (t, 2H), δ 7.94 (d, 2H), δ 7.83(t, 2H), δ 4,51(s, 4H), δ 3.45 (m, 8H), δ 3.15 (m, 8H); ESI MS (m/Z) 456 (**4** + H^+^). ^13^C-NMR (400 MHz, D_2_O, pD = 2): δ(ppm) 154.62, 146.54, 140.20, 135.22, 130.15, 129.60, 128.69, 122.84, 121.67, 55.46, 48.34, 43.22. ESI MS (m/Z) 455.2906 (**4** + H^+^).

### 4,10-bis(methylen-2-quinolyl)-1,4,7,10-tetraazacyclododecane-1,7-diacetic acid (**5**)

Synthesis of this compound was performed with a similar procedure to that reported previously^17^. A solution of chloroacetic acid (3.03 g, 32.1 mmol) in water (10 mL) was added to a solution of **4** (0.97 g, 2.14 mmol) in water (10 mL), adjusting pH to 9.5 by using a 5 M NaOH solution. Temperature was kept at 70 °C for 18 h. The pH of the solution was maintained at 9.5 with drops of 5 M NaOH. The solution was then dried by evaporation, the crude residue dissolved in water (20 mL), and the pH of the solution adjusted to 7 by addition of 5 M HCl. The solution was then passed through a cation-exchange column (Amberlite IR 120; acidic form, bed volume 60 cm^3^), which was eluted with water (500 mL), then with 0.5 M NH_3_ (600 mL) and finally with water (700 mL). Each fraction was vacuum-evaporated and analysed by ^1^H NMR. The fractions containing the desired product were collected together and the resulting solution was vacuum evaporated, yielding the ligands as ammonium salt. This compound was dissolved in 10 mL water and passed through a column filled with anion-exchange resin (Amberlite IRA 900, alkaline form, bed volume 50 mL), eluted with water (350 mL), then with 5 M HCl (350 mL) and finally with 0.1 M HCl (300 mL). Each fraction was vacuum-evaporated and analyzed by ^1^H NMR. The fractions containing the desired product were combined and dried under vacuum. The resulting yellow solid was recrystallized from ethanol/H_2_O mixture to afford compound **5** as anhydrous tetrahydrochloride salt (**5**·4HCl·2H_2_O). Yield 0.53 g (33%). Anal. calcd. for C_32_H_46_N_6_O_6_Cl_4_: C 51.07, N 11.17, H 6.16; found C 51.15, N 11.29, H 6.24; ^1^H-NMR (400 MHz, D_2_O, pD = 2): δ(ppm) 9.20 (d, 2H), 8.44 (d, 2H), 8.35 (m, 4H), 8.29 (d, 2H), 8.04 (t, 2H), 4.24 (s, 4H), 3.65 ppm (m, 4H); 3.56 (m, 4H), δ 3.26 (m, 4H), 3.02 (m, 4H); ^13^C-NMR (400 MHz, D_2_O, pD = 2): δ(ppm) 168.24, 153.17, 148.49, 139.16, 136.29, 131.10, 129.77, 129.20, 123.97, 121.27, 56.13, 55.55, 51.69, 48.33. ESI MS (m/Z) 571.3049 (**5** + H^+^).

### [MnQ2]·2H_2_O

A deoxygenated aqueous solution of NaOH 0.1 M (9.2 mL, 0.92 mmol) was added to deoxygenated solution (10 mL) of **5**·4HCl·2H_2_O (177 mg, 0.23 mmol) in water. To this solution, heated to reflux, a solution of MnCl_2_·4H_2_O (35.6 mg, 0.18 mmol) in deoxygenated water was slowly added in about 4 h under stirring. The volume of the solvent was then reduced at 5 mL by heating at 80 °C under nitrogen flow. To the resulting solution, ethanol (20 mL) and diethyl ether (10 mL) were added to yield a white precipitate, which was filtered off and recrystallized from a water/ethanol 1:15 mixture. Yield 57 mg (51.0%). Anal. calcd. for C_32_H_40_N_6_O_6_Mn: C, 58.25; H, 6.11; N, 12.74; Mn 8.33; Found: C, 58.33; H, 6.04; N, 12.91; Mn 8.3. ESI MS (m/Z): 624.2238 ([MnQ2 + H]^+^).

### [ZnQ2]·3H_2_O

This compound was prepared by addition of an aqueous solution of ZnCl_2_·6H_2_O (5.3 mg, 0.022 mmol) to an aqueous solution (5 mL) of **5**·4HCl·2H_2_O (16.5 mg, 0.022 mmol) by using the same procedure reported for [MnQ2]·2H_2_O. Yield 12.5 mg (82.6%). Anal. calcd. for C_32_H_42_N_6_O_7_ Zn: C, 55.86; H, 6.15; N, 12.21; Found: C, 55.56; H, 6.20; N, 12.30. ESI MS (m/Z): 633.2157 ([ZnQ2 + H]^+^). ^1^H-NMR (400 MHz, D_2_O, pH = 7.4): δ 8.56 (d, 2H), δ 8.44 (d, 2H, H4,4′), δ 8.04 (d, 2H), δ 7.61 (m, 6H), δ 4.35 (s, 1H), δ 4.17 (m, 1H), δ 4.01 (m, 1H), δ 3.73 (m, 1H), δ 3.17 (m, 12H), δ 2.97 (m, 1H), δ 2.82 (m, 6H), δ 2.61 (m, 1H); ^13^C-NMR (400 MHz, D_2_O, pD = 7.3): δ (ppm) 179.79, 164.91. 160.93, 158.13, 147.40, 146.79, 140.49, 139.92, 131.42, 131.01, 129.11, 128.89, 128.51, 128.29, 128.03, 127.98, 127.51, 127.35, 123.20, 122.41, 60.52, 59.88, 59.40, 56.31, 52.82, 51.32, 50.76, 48.30.

### NMR experiments

All ^1^H and ^13^C spectra were recorded on a Bruker Avance III 400 MHz instrument. For H_2_Q2, the ^1^H spectrum was collected at pH 2 and pH 7.4, while the ^13^C spectrum was recorded only at pH 2. In fact, at pH 7.4 this ligand shows limited solubility in water, even at 100 °C, which precludes recording of the ^13^C spectrum. The ^1^H NMR spectra of H_2_Q_2_ in the presence of increasing amounts of Mn^II^ (as MnSO_4_ salt) were collected at 400 MHz and at 900 MHz on a Bruker NEO 900‐MHz equipped with 5 mm CP TCI ^1^H/^13^C/^15^N z grd probe. In this experiment, a de-areated MnSO_4_ solution (0.3 M) in D_2_O was added to a de-areated boiling solution of H_2_Q_2_ in D_2_O (1 · 10^−2^ M), phosphate-buffered at pH 7.2. After the addition, the solution was kept at 100 °C for 4 h before collecting the spectrum. The ^1^H NMR spectra of H_2_Q_2_ in the presence of increasing amounts of Zn^II^ (as ZnCl_2_ salt) were collected at 400 MHz. This experiment was performed in the same conditions described for Mn^II^ complexation.

### EPR Measurements

X-band EPR Spectra were recorded in 0.5 mM frozen solutions of 1:1 (v/v) H_2_O/glycerol at 100 K using a Bruker Elexsys E500 spectrometer, equipped with a Helium flow ESR900 cryostat (Oxford Instruments). Acquisition parameters: microwave frequency: 9.3972 GHz; microwave power 2.1 mW; modulation amplitude 2 G; modulation frequency 100 KHz.

### ESI-MS spectroscopy

ESI-MS spectra were recorded on a LTQ Orbitrap high-resolution mass spectrometer. In a typical experiment, stock solutions of the samples (10^−3^ M) were prepared in methanol (compound **3**) or water (compounds **4**, **5**, ZnQ2 and MnQ2). After a 50-fold dilution in MeOH, high resolution ESI mass spectra were recorded by direct introduction at 5 μL min^−1^ flow rate in an LTQ-orbitrap high-resolution mass spectrometer (Thermo, San Jose, CA, USA), equipped with a conventional ESI source. The working conditions were: spray voltage 5 kV, capillary voltage 39 V, capillary temperature 280 °C, tube lens voltage 130 V. The sheath and the auxiliary gases were set, respectively, at 10 and 5 (arbitrary units).

### Potentiometric measurements

All pH measurements (pH = −log [H^+^]) employed for the determination of ligand protonation and metal complex stability constants were carried out in 0.10 M NMe_4_Cl aqueous solution at 298.1 ± 0.1 K by conventional titration experiments under inert atmosphere. The equipment and procedure used were as previously described^50^. The standard potential *E*° and the ionic product of water (p*K*_w_ = 13.83(1) at 298.1 ± 0.1 K in 0.10 M NMe_4_Cl) were determined by Gran’s method^51^. At least three measurements (with about 100 data points for each) were performed for each system in the pH ranges 2–10.5. In all experiments, ligand concentration [L] was about 0.5 × 10^−3^ M. In the complexation experiments the metal ion concentration was changed from 0.5:1 to 1.5:1. The computer program HYPERQUAD^37,52^ was used to calculate both protonation and stability constants from potentiometric data. Distribution diagrams and competition plots were calculate by using the Hyss program^37^.

### *In vitro*${{\bf{O}}}_{{\bf{2}}}^{{\boldsymbol{\cdot }}{\boldsymbol{-}}}$ dismutation by MnQ2 and MnM2

Xanthine oxidase catalyzes the oxidation of xanthine to uric acid with concomitant reduction of molecular oxygen to ${{\rm{O}}}_{2}^{\cdot -}$ and hydrogen peroxide. The MnQ2- and MnM2-dependent dismutation of ${{\rm{O}}}_{2}^{\cdot -}$ was monitored by following ${{\rm{O}}}_{2}^{\cdot -}$-dependent cytochrome *c* reduction spectrophotometrically at 550 nm and 37 °C in the presence of catalase. Reactions was carried out in 750 μL potassium phosphate buffer 50 mM, pH 6.8, containing 0.1 mM EDTA, 400 U mL^−1^ catalase, 6 mM cytochrome *c* and 0.02 U mL^−1^ xanthine oxidase. Reactions were initiated by adding 50 μL xanthine 800 μM. Optical absorption curves were acquired for 350 seconds using a Jasco V-630 ultraviolet spectrophotometer. Curves were also acquired in the absence of MnQ2 (50 μM), MnM2 (50 μM), and in the presence of 500 U mL^−1^ SOD. Three independent measurements were performed. Xanthine oxidase from bovine milk, cytochrome *c* from horse heart, SOD, catalase, xanthine, potassium phosphate and EDTA were purchased from Sigma (St Louis, MO).

### Cell culture

H9c2 embryonic rat cardiac muscle cells, obtained from European Collection of Cell Cultures (ECACC, Salisbury, UK), were cultured in Dulbecco’s modified Eagle’s medium (DMEM) supplemented with 10% heat-inactivated fetal bovine serum (FBS, Invitrogen, Carlsbad, CA, USA), 2 mM glutamine, 250 U mL^−1^ penicillin G and 250 μg mL^−1^ streptomycin, in a humidified atmosphere with 5% CO_2_ at 37 °C.

Preliminary experiments were performed to assess the toxicity of MnQ2 and ZnQ2, which were added to H9c2 cell cultures (5 × 10^4^/well in 24-well plates) at a 10-fold higher concentration (100 μmol L^−1^) than that used for the experiments (10 μmol L^−1^) for 24 h. Under these conditions, cell viability assayed by the 3-(4,5-dimethylthiazol-2-yl)-2,5-diphenyl tetrazolium bromide (MTT) test was not impaired in comparison with the untreated cultures (data not shown). Lack of toxicity of MnM2 and ZnM2 were demonstrated previously with the same method^18^.

### Cell permeation

To investigate the kinetics of cell permeation of MnQ2 and MnM2, H9c2 cells were seeded in 24-well plates (5 × 10^4^ cells/well) and allowed to adhere. The medium was replaced with phosphate-buffered saline, either alone (controls) or added with MnQ2 or MnM2 (10 μmol L^−1^), and the cells were incubated for 30 min, 1 h or 2 h. Then, the medium was removed and the cells were thoroughly washed with phosphate-buffered saline. Finally, the cells were detached in distilled water with a cell scraper and subjected to a first centrifugation at 1200 *g* for 5 min to separate the nuclei, the pellet was discarded and the supernatant was centrifuged again at 10,000 *g* for 10 min to separate a pelleted fraction containing mitochondria and a supernatant fraction corresponding to cytosol^53^. In both fractions, Mn, assumed as indicator for MnQ2 and MnM2 permeation, was measured by ICP-AES. Raw Mn values were normalized to cellular proteins, evaluated by the micro-bicinchoninic acid (BCA) method, and expressed as ng μg^−1^ of proteins. These experiments were performed in triplicate.

### Cellular oxidative stress

A cellular model of oxidative stress was used to investigate the SOD-mimetic properties of MnQ2 and MnM2. Briefly, H9c2 cells were subjected to hypoxia and reoxygenation (H + R) as previously described^18^. The cells were incubated in Dulbecco modified Eagle’s medium (DMEM) with no serum or glucose and placed in a hypoxic chamber saturated with a 0.1% O_2_, 5% CO_2_, 95% N_2_ gaseous mix, humidified and warmed at 37 °C, for 7 h. At end hypoxia, the cells were incubated in normoxic conditions in glucose-containing, serum-free DMEM. Cells were treated with either MnQ2 or MnM2 at concentration of 10 μmol L^−1^, based on our previous *in vitro* studies^18,19^, added to the medium at the time of reoxygenation concurrently with the climax of ROS generation^42,43^ and maintained for 1 or 2 h As controls for the specific capability of MnQ2 and MnM2 to suppress oxidative stress by ${{\rm{O}}}_{2}^{\cdot -}$ dismutation, some cell viability experiments were performed using the inactive congeners ZnQ2 and ZnM2 at the same concentration (10 μmol L^−1^) added at reoxygenation.

### Mitochondrial number

Preliminary experiments were performed to evaluate the amount of mitochondria in control and treated cells to exclude that possible changes in mitochondrial function could be due to uneven mitochondrial numbers. Briefly, H9c2 cells seeded on glass coverslips were loaded with MitoTracker Deep Red 633 mitochondrial fluorescent dye (0.5 μM, Life Technologies, Carlsbad, CA, USA) dissolved in 0.1% DMSO and Pluronic acid F-127 (0.01% w/v), added to the culture medium for 20 min at 37 °C. Cells were fixed in 2% buffered paraformaldehyde for 10 min at room temperature and red fluorescence was analyzed using a Leica TCS SP5 confocal scanning microscope (Leica, Mannheim, Germany) equipped with an argon laser source (excitation λ 633 nm) and a x63 oil immersion objective. Mitochondrial amount was also measured by flow cytometry. Single-cell suspensions were incubated with MitoTracker Deep Red 633 (200 nM) for 20 min at 37 °C and immediately analysed with a FACSCanto flow cytometer (Becton–Dickinson, San Jose, CA). Data were analyzed using FACSDiva software (Becton–Dickinson). Since both methods detected no substantial differences among the different experimental groups, the results of the subsequent experiments to assess mitochondrial function were deemed reliable.

### Intracellular ROS and mitochondrial ${{\bf{O}}}_{{\bf{2}}}^{{\boldsymbol{\cdot }}{\boldsymbol{-}}}$

H9c2 cells seeded on glass coverslips were loaded with the ROS-sensitive fluorescent probe 2′,7′-dichlorodihydrofluorescein diacetate (H_2_DCFDA; Invitrogen, CA, USA; 2.5 μmol L^−1^) or the mitochondrial ${{\rm{O}}}_{2}^{\cdot -}$-specific fluorescent probe MitoSOX (Invitrogen; 3 μmol L^−1^) - dissolved in 0.1% DMSO and Pluronic acid F-127 (0.01% w/v) – which were added to cell culture media for 15 min at 37 °C, as described^18^. The cells were fixed in 2% buffered paraformaldehyde for 10 min at room temperature and the H_2_DCFDA and MitoSOX fluorescence analysed using a Leica TCS SP5 confocal scanning microscope equipped with an argon laser source (excitation λ 488 nm or 543 nm, respectively) and a x63 oil immersion objective. ROS and mitochondrial ${{\rm{O}}}_{2}^{\cdot -}$ generation were also monitored by flow cytometry^54^: briefly, single-cell suspensions were incubated with H_2_DCFDA (1 μmol L^−1^) or MitoSOX (0.5 μmol L^−1^) for 15 min at 37 °C and immediately analysed using a FACSCanto flow cytometer (Becton–Dickinson). Data were analyzed using FACSDiva software (Becton–Dickinson).

### Mitochondrial activity

This was measured using the fluorometric resazurin reduction method (CellTiter-Blue, Promega Corp.). Resazurin is a redox dye commonly used as an indicator of chemical cytotoxicity in cultured cells. The assay is based on the ability of viable, metabolically active cells to reduce resazurin to resorufin and dihydroresorufin, proportionally to their number. Conversion occurs intracellularly and is facilitated by mitochondrial, microsomal and cytosolic oxido-reductases. Resorufin produced by resazurin bioreduction was measured fluorometrically (excitation λ 571 nm). Resazurin is non-toxic to cells and stable in culture medium, allowing continuous measurement of cell proliferation *in vitro* as either a kinetic or endpoint assay.

### Mitochondrial membrane potential (Δψ)

Mitochondrial membrane potential was assessed using tetramethylrhodamine methyl ester perchlorate (TMRM), a lipophilic potentiometric fluorescent dye whose accumulation in mitochondria is directly related to mitochondrial potential Δψ, as described^18^. For confocal microscope analysis, cells were cultured on glass coverslips and loaded for 20 min at 37 °C with TMRM, dissolved in 0.1% DMSO to a 100 nM final concentration in the culture medium. The cells were fixed in 2% buffered paraformaldehyde for 10 min at room temperature and the TMRM fluorescence analyzed under a confocal Leica TCS SP5 scanning microscope equipped with a helium-neon laser source (excitation λ 543 nm) and a x63 oil immersion objective. Mitochondrial membrane potential was also quantified by flow cytometry, as described^18,55^. Single-cell suspensions were washed twice with phosphate-buffered saline (PBS) and incubated for 20 min at 37 °C in the dark with TMRM dissolved in DMEM (100 nM). The cells were then washed, resuspended in PBS and analyzed using a FACSCanto flow cytometer (Becton-Dickinson).

### Mitochondrial permeability transition pore opening (mPTP)

Mitochondrial permeability, an index of mitochondrial dysfunction and early apoptosis, was measured by calcein fluorescence, as described^18,56^. The fluorescent probe calcein-AM readily enters into cells and emits fluorescence upon de-esterification. Co-loading of cells with cobalt chloride, which cannot cross the mitochondrial membranes in living cells, quenches calcein fluorescence in the whole cell except mitochondria. During induction of mPTP, cobalt can enter mitochondria and quenches calcein fluorescence, whose decrease can be taken as a measure of the extent of mPTP induction. H9c2 cells grown on glass coverslips were loaded with calcein-AM (3 µM) and cobalt chloride (1 mM) added to the culture medium for 20 min at 37 °C. The cells were then washed in PBS, fixed in 2% buffered paraformaldehyde for 10 min at room temperature and analyzed by a Leica TCS SP5 confocal laser scanning microscope equipped with an argon laser source (excitation λ 488 nm) and a x63 oil immersion objective. Mitochondrial permeability was also monitored by flow cytometry: single-cell suspensions were incubated with calcein-AM (3 µM) and cobalt chloride (1 mM) for 20 min at 37 °C, washed twice with PBS and analyzed using a FACSCanto flow cytometer (Becton-Dickinson).

### Evaluation of lipid peroxidation

Lipid peroxidation was investigated by confocal scanning microscopy using BODIPY 581/591 C11 (Life Technologies, Carlsbad, CA, USA), a fluorescent probe that is intrinsically lipophilic and thus mimics the properties of natural lipids, as described^54,57^. BODIPY 581/591 C11 acts as a fluorescent lipid peroxidation reporter that shifts its fluorescence from red to green in the presence of oxidizing agents. Briefly, cells were cultured on glass coverslips and loaded with BODIPY, dissolved in 0.1% DMSO (2.5 mM final concentration), added to the cell culture media for 15 min at 37 °C. The cells were fixed in 2.0% buffered paraformaldehyde for 10 min at room temperature and the BODIPY fluorescence analyzed (excitation λ 581 nm) using a confocal Leica TCS SP5 scanning microscope equipped with an argon laser source for fluorescence measurements. A series of optical sections (1024 × 1024 pixels) 1.0 μm in thickness was taken through the cell depth at intervals of 0.5 μm using a Leica Plan Apo 63X oil immersion objective and then projected as a single composite image by superimposition. Moreover, lipid peroxidation was quantified by flow cytometry. Single-cell suspensions were washed twice with PBS and incubated, in the dark, for 30 min at 37 °C with BODIPY 581/591 (2.5 mM) in DMEM. After labeling, cells were washed and resuspended in PBS and analyzed using a FACSCanto flow cytometer (Becton-Dickinson, San Jose, CA).

### Assessment of caspase activity

Since mitochondrial dysfunction is a well-known trigger of apoptosis, we next investigated the activation of pro-apoptotic initiator caspases 8 (extrinsic pathway) and 9 (intrinsic pathway), and effector caspase 3 and the possible influence of H + R, as described^18^. Briefly, MnQ2 and MnM2. H9c2 cells seeded on glass coverslips were incubated with FAM-FLICA™ Caspase assay kit (Immunochemistry Technologies, Bloomington, MN, USA) for 30 min, following the manufacturer’s instructions. After incubation, the cells were thoroughly washed and fixed in 2% buffered paraformaldehyde for 10 min at room temperature. Fluorescence was detected by a confocal Leica TCS SP5 scanning microscope equipped with an argon laser source (excitation λ 488 nm) and a x63 oil immersion objective. Caspase activity was also quantified by flow cytometry, as previously reported^18,58^ single-cell suspensions were incubated with FAM-FLICA™ for 30 min at 37 °C, washed twice with PBS and analyzed using a FACSCanto flow cytometer (Becton-Dickinson).

### Cell death assay

Lactate dehydrogenase (LDH) activity, accounting for cell death, was assessed spectrophometrically in the culture medium and in adherent cells (in order to obtain total LDH content) using the LDH assay kit (Roche Diagnostics, Mannheim, Germany). LDH release was calculated as a percentage of total LDH content.

### Assessment of MAPK activity

Phosphorylation of MAPKs was assessed as activation index. Briefly, H9c2 cells exposed to H + R and treated for 1 h with MnQ2 or MnM2 were fixed and permeabilized with Cytofix/Cytoperm buffer (Becton-Dickinson) following the manufacturer’s instructions. Then, the cells were incubated for 1 h at room temperature with antibodies against phospho-JNK (Thr183/Tyr185; G9 mouse monoclonal, phycoerythrin-conjugated), phospho-p38 (Thr180/Tyr182; 28B10 mouse monoclonal, Alexa Fluor 488-conjugated) and phospho-p44/42 MAPK (Thr202/Tyr204; D13.14.4E rabbit polyclonal, Alexa Fluor 488-conjugated) (Cell Signaling Technology, Leiden, Netherlands). After washed twice with PBS, cells were analyzed using a FACSanto flow cytometer (Becton-Dickinson).

### Statistical analysis

The reported data are expressed as the mean ± SEM of at least 3 independent experiments. Statistical comparison of differences between groups was carried out using one-way analysis of variance (ANOVA) followed by Student-Newman-Keuls multiple comparison test. Comparisons between MnQ2 and MnM2 were performed with Student’s *t* test for unpaired values. A p value ≤ 0.05 was considered significant. Calculations were done using GraphPad Prism 5.0 statistical program (GraphPad Software, San Diego, CA, USA).

## Supplementary information

Supplementary information 1

Supplementary information 2

Supplementary information 3

## References

[1] Harman D (1956). Aging: a theory based on free radical and radiation chemistry. J. Gerontol..

[2] Jang YC (2009). Overexpression of Mn superoxide dismutase does not increase life span in mice. J. Gerontol. A Biol. Sci. Med. Sci..

[3] Pérez VI (2009). The overexpression of major antioxidant enzymes does not extend the lifespan of mice. Aging Cell..

[4] Bjelakovic G, Nikolova D, Gluud C (2014). Antioxidant supplements and mortality. Curr. Opin. Clin. Nutr. Metab. Care.

[5] Harman D (1972). The biologic clock: the mitochondria?. J. Am. Geriatr. Soc..

[6] Harman D (1981). The aging process. Proc. Natl. Acad. Sci. USA.

[7] Pascual-Ahuir Amparo, Manzanares-Estreder Sara, Proft Markus (2017). Pro- and Antioxidant Functions of the Peroxisome-Mitochondria Connection and Its Impact on Aging and Disease. Oxidative Medicine and Cellular Longevity.

[8] Kujoth GC (2005). Mitochondrial DNA mutations, oxidative stress, and apoptosis in mammalian aging. Science.

[9] Paul A (2007). Reduced mitochondrial SOD displays mortality characteristics reminiscent of natural aging. Mech. Ageing Dev..

[10] Skulachev VP (2009). An attempt to prevent senescence: a mitochondrial approach. Biochim. Biophys. Acta.

[11] Oyewole AO, Birch-Machin MA (2015). Mitochondria-targeted antioxidants. FASEB J..

[12] Johnson F, Giulivi C (2005). Superoxide dismutases and their impact upon human health. Mol. Aspects Med..

[13] Riley DP, Weiss RH (1994). Manganese macrocyclic ligand complexes as mimics of superoxide dismutase. J. Am. Chem. Soc..

[14] Bani D, Bencini A (2012). Developing ROS scavenging agents for pharmacological purposes: recent advances in design of manganese-based complexes with anti-inflammatory and anti- nociceptive activity. Curr. Med. Chem..

[15] Batinic-Haberle I (2014). SOD Therapeutics: latest insights into their structure-activity relationships and impact on the cellular redox-based signaling pathways. Antioxid. Redox Signal..

[16] Batinic-Haberle I, Tovmasyan A, Spasojevic I (2018). Mn Porphyrin-based redox-active drugs: differential effects as cancer therapeutics and protectors of normal tissue against oxidative injury. Antiox. Redox. Signal..

[17] Failli P (2009). A novel manganese complex effective as superoxide anion scavenger and therapeutic agent against cell and tissue oxidative injury. J. Med. Chem..

[18] Nistri S (2015). A new low molecular weight, MnII-containing scavenger of superoxide anion protects cardiac muscle cells from hypoxia/reoxygenation injury. Free Radic. Res..

[19] Becatti M (2015). Protection of coronary endothelial cells from cigarette smoke-induced oxidative stress by a new Mn(II)-containing polyamine-polycarboxilate scavenger of superoxide anion. Vascul. Pharmacol..

[20] Bianchi A (2000). Thermodynamics and structural properties of Gd(III) complexes with polyamino-polycarboxylic ligands: basic compound for the development of MRI contrast agents. Coord. Chem. Rev..

[21] Merbach, A. E. & Tóth, E. *The chemistry of contrast agents in medical magnetic resonance imaging*. (John Wiley & Sons, 2001).

[22] Spasojevic I (2010). Accumulation of porphyrin-based SOD mimics in mitochondria is proportional to their lipophilicity. *S*. *cerevisiae* study of ortho Mn(III) N-alkylpyridylporphyrins. Free Radic. Biol. Med..

[23] Le Baccon M (2001). Bis-aminals: efficient tools for bis-macrocycle synthesis. New J. Chem..

[24] Martell, A. E. & Smith, R. M. *NIST Critically Selected Stability Constants of Metal Complexes* NIST Standard Reference Database 46 Version 8.0: Gaithersburg, MD: Standard Reference Data Program, National Institute of Standards and Technology, U.S. Dept. of Commerce (2004).

[25] Bianchi, A. *et al*. Thermodynamic and structural aspects of manganese(II) complexes with polyaminopolycarboxylic ligands based upon 1,4,7,10-tetraazacyclododecane (cyclen). Crystal structure of dimeric [MnL]_2_·2CH_3_OH containing the new ligand 1,4,7,10-tetraazacyclododecane-1,4-diacetate. *J*. *Chem*. *Soc*., *Dalton Trans*. 917–922 (2001).

[26] Wang S, Westmoreland TD (2009). Correlation of relaxivity with coordination number in six, seven-, and eight-coordinate Mn(II) complexes of pendant-arm cyclen derivatives. Inorg. Chem..

[27] Garda Z (2016). Physico-chemical properties of Mn^II^ complexes formed with cis- and trans-DO2A: thermodynamic, electrochemical and kinetic studies. J Inorg Biochem..

[28] Drahos, B., Lukes, I. & Tóth, E. Manganese(II) complexes as potential contrast agents for MRI, *Eur*. *J*. *Inorg*. *Chem*., 1975–1986 (2012).

[29] de Sá A (2013). Thermodynamic stability and relaxation studies of small, triaza-macrocyclic Mn(II) chelates. Dalton Trans..

[30] Barbaro, P. *et al*. Synthesis and characterization of the tetraazamacrocycle 4,10-dimethyl-1,4,7,10-tetraazacyclododecane-1,7-diacetic acid (H_2_Me_2_DO_2_A) and of its neutral copper(II) complex [Cu(Me_2_DO_2_A)]. A new 64Cu-labeled macrocyclic complex for positron emission tomography imaging. *J*. *Chem*. *Soc*., *Dalton Trans*. 2393–2401 (2000).

[31] Ye B-H, Li X-Y, Williams I-D, Chen X-M (2002). Synthesis and structural characterization of di- and tetranuclear zinc complexes with phenolate and carboxylate bridges. correlations between ^13^C NMR chemical shifts and carboxylate binding modes. Inorg. Chem..

[32] Wait CS, McNerney JC (1970). Vibrational spectra and assignments for quinoline and isoquinoline. J. Mol. Spectr..

[33] Ataç A, Yurdakul S, Berber S (2011). Synthesis, spectroscopy, and characterization of some bis nicotinamide metal(II) dihalide complexes. Spectrochim. Acta Part A.

[34] Idec S (2006). Synthesis and vibrational spectroscopic studies of isonicotinamide metal(II) halide complexes. J. Mol. Struct..

[35] Bayarı S, Ataç A, Yurdakul S (2003). Coordination behaviour of nicotinamide: an infrared spectroscopic study. J. Mol. Struct..

[36] Bianchi A, Garcia-España E (1999). The use of calculated species distribution diagrams to analyze thermodynamic selectivity. J. Chem. Ed..

[37] Alderighi L (1999). Hyperquad simulation and speciation (HySS): a utility program for the investigation of equilibria involving soluble and partially soluble species. Coord. Chem. Rev..

[38] Ashkenazi A (2008). Directing cancer cells to self-destruct with pro-apoptotic receptor agonists. Nat. Rev. Drug Discov..

[39] Kim EK, Choi EJ (2010). Pathological roles of MAPK signaling pathways in human diseases. Biochim. Biophys. Acta..

[40] Gu L (2009). Involvement of ERK1/2 signaling pathway in DJ-1-induced neuroprotection against oxidative stress. Biochem. Biophys. Res. Commun..

[41] Pizzino Gabriele, Irrera Natasha, Cucinotta Mariapaola, Pallio Giovanni, Mannino Federica, Arcoraci Vincenzo, Squadrito Francesco, Altavilla Domenica, Bitto Alessandra (2017). Oxidative Stress: Harms and Benefits for Human Health. Oxidative Medicine and Cellular Longevity.

[42] Murphy MP (2009). How mitochondria produce reactive oxygen species. Biochem. J..

[43] Poyton RO, Ball KA, Castello PR (2009). Mitochondrial generation of free radicals and hypoxic signaling. Trends Endocrinol. Metab..

[44] Weekley CM (2017). Cellular fates of Manganese(II) pentaazamacrocyclic superoxide dismutase (SOD) mimetics: fluorescently labeled MnSOD mimetics, X-ray absorption spectroscopy, and X-ray fluorescence microscopy studies. Inorg. Chem..

[45] Shin SW (2017). Mechanism of the antitumor and radiosensitizing effects of a manganese porphyrin, MnHex-2-PyP. Antioxid. Redox Signal..

[46] Liu J (2013). Recombinant PTD-Cu/Zn SOD attenuates hypoxia-reoxygenation injury in cardiomyocytes. Free Radic. Res..

[47] McCord JM, Edeas MA (2005). SOD, oxidative stress and human pathologies: a brief history and a future vision. Biomed. Pharmacother..

[48] Slemmer JE, Shacka JJ, Sweeney MI, Weber JT (2008). Antioxidants and free radical scavengers for the treatment of stroke, traumatic brain injury and aging. Curr. Med. Chem..

[49] Clinicaltrials.gov identifier: NCT03386500, NCT03608020, NCT02990468, NCT03726294, NCT02655601, NCT03689712.

[50] Bencini A (1996). Effect of nitrogen methylation on cation and anion coordination by hexa- and heptaazamacrocycles. catalytic properties of these ligands in atp dephosphorylation. Inorg. Chem..

[51] Gran G (1952). Determination of the equivalence point in potentiometric titrations, part II. Analyst (London).

[52] Gans P, Sabatini A, Vacca A (1996). Investigation of equilibria in solution. Determination of equilibrium constants with HYPERQUAD suite of programs. Talanta.

[53] Afanasyeva MA (2018). Isolation of large amounts of highly pure mitochondria for “omics” studies. Biochemistry (Mosc).

[54] Becatti M (2014). SIRT1 regulates MAPK pathways in vitiligo skin: insight into the molecular pathways of cell survival. J. Cell. Mol. Med..

[55] Pini A (2016). Protection from cigarette smoke-induced vascular injury by recombinant human relaxin-2 (serelaxin). J. Cell. Mol. Med..

[56] Petronilli V (1999). Transient and longlasting openings of the mitochondrial permeability transition pore can be monitored directly in intact cells by changes in mitochondrial calcein fluorescence. Biophys. J..

[57] Pensalfini A (2008). Protective effect of new S-acylglutathione derivatives against amyloid-induced oxidative stress. Free Radic. Biol. Med..

[58] Becatti M (2010). The involvement of Smac/DIABLO, p53, NF-kB, and MAPK pathways in apoptosis of keratinocytes from perilesional vitiligo skin: Protective effects of curcumin and capsaicin. Antioxid. Redox Signal..

