# Supplementary material for: Different Antioxidant Efficacy of Two MnII-Containing Superoxide Anion Scavengers on Hypoxia/Reoxygenation-Exposed Cardiac Muscle Cells
Source: Sci Rep. 2019 Jul 16;9:10320. doi: 10.1038/s41598-019-46476-2 (PMC6635543; doi:10.1038/s41598-019-46476-2)
Supplement: Supplementary file 1 — Supplementary information 1 [file 41598_2019_46476_MOESM1_ESM.pdf]

DIFFERENT ANTIOXIDANT EFFICACY OF TWO Mn^{II}-CONTAINING SUPEROXIDE ANION SCAVENGERS ON HYPOXIA/REOXYGENATION-EXPOSED CARDIAC MUSCLE CELLS.

Matteo Becatti, Andrea Bencini, Silvia Nistri, Luca Conti, Maria Giulia Fabbrini, Laura Lucarini, Veronica Ghini, Mirko Severi, Claudia Fiorillo, Claudia Giorgi, Lorenzo Sorace, Barbara Valtancoli, Daniele Bani

Supplementary information 1 - Spectra (NMR, high resolution mass, X-band EPR, IR) of the compounds under study.

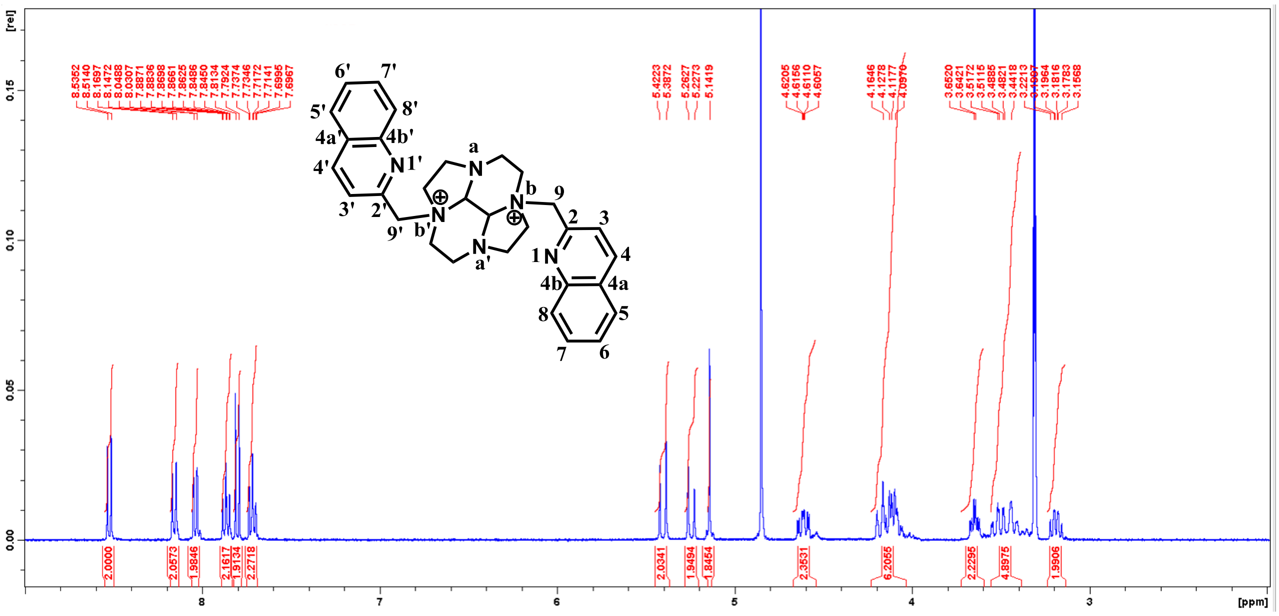

Figure S1. ^{1}H-NMR spectrum (400 MHz, MeOD) of compound **3**: δ(ppm) 8.52 (d, 2H, H8,8’), 8.15 (d, 2H, H4,4’), 8.04 (d, 2H, H5,5’), 7.86 (t, 2H, H7,7’), 7.81 (d, 2H, H3,3’), 7.72 (t, 2H, H6,6’), 5.41-5.24 (dd, 4H, 9,9’), 5.15 (s, 2H, CH-CH bridge), 4.61 (m, 2H), 4.13 (m, 6H), 3.65 (m, 2H), 3.47 (m, 4H), 3.19 (m, 2H). The latter four signals are attributable to the ethylene linkers.

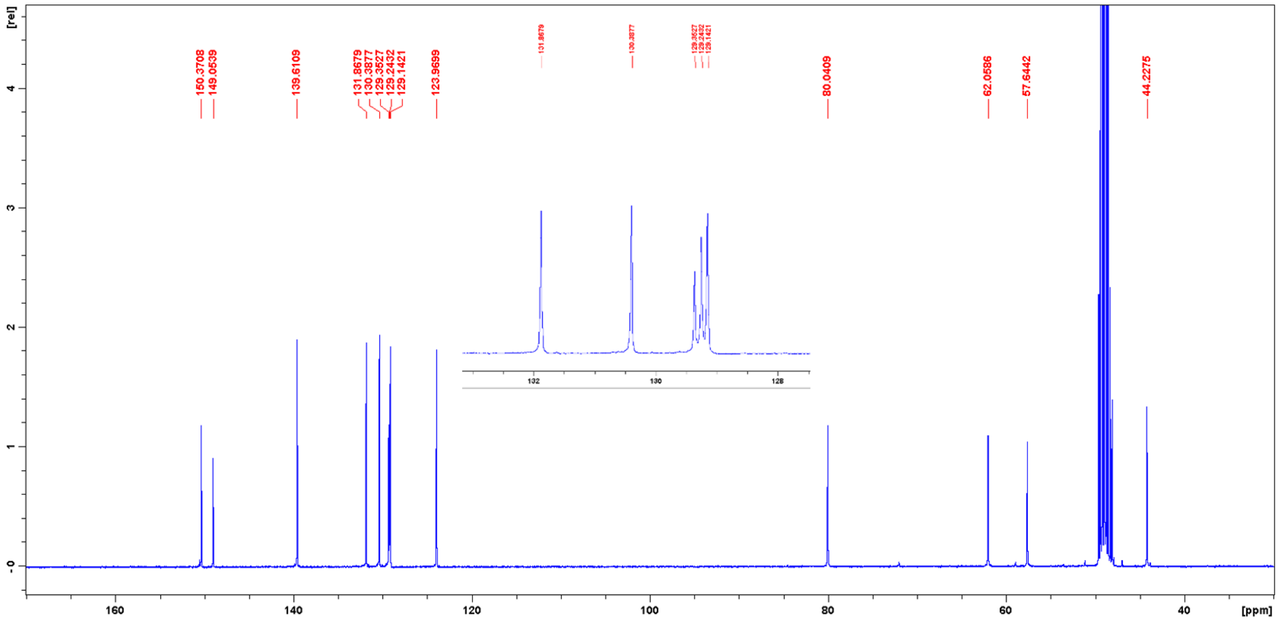

Figure S2. ^{13}C-NMR spectrum (400 MHz, MeOD) of compound **3**: δ(ppm) 150.37 (C2), 149.05 (C4b), 139.61 (C4), 131.87 (C7), 130.39, 129.35, 129.24, 129.14, 123.97, 80.04, 62.06, 57.64, 44.23.

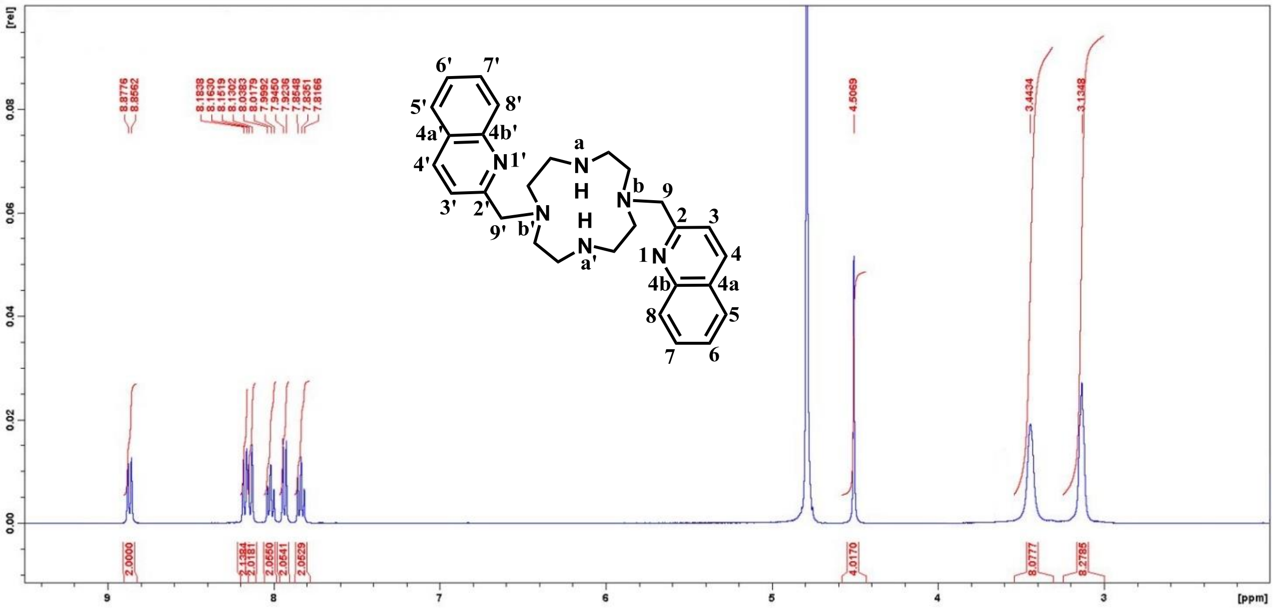

Figure S3. ^{1}H-NMR spectrum (400 MHz, D_2O, pH = 2) of compound **4:** ^{1}H-NMR (400 MHz, D_2O, pD = 2): δ(ppm) 8.86 (d, 2H, H8,8'), 8.17 (d, 2H, H4,4'), 8.14 (d, 2H, H5,5'), 8.02 (t, 2H, H7,7'), 7.94 (d, 2 H, H3,3'), 7.83 (t, 2H, H6,6'), 4.51 (s, 4H, H9), δ 3.45 (m, 8 H), δ 3.15 (m, 8H). The latter two signals are attributable to the ethylene linkers.

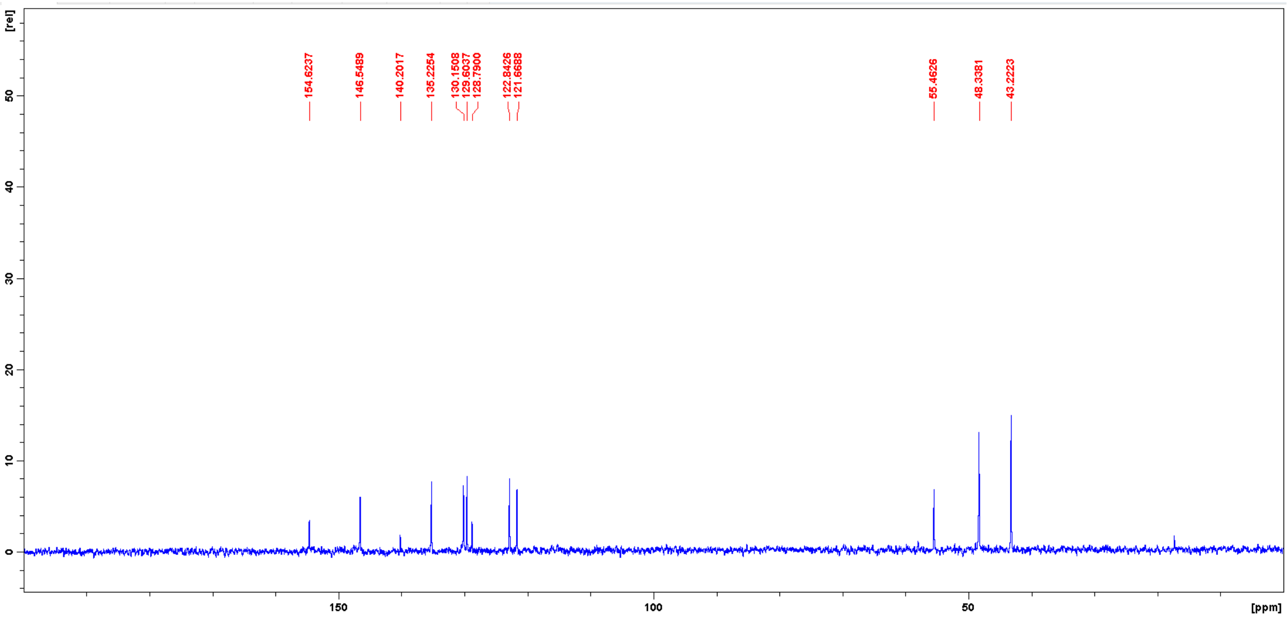

Figure S4. ^{13}C-NMR spectrum (400 MHz, D_2O, pH = 2) of compound **4:** δ(ppm) 154.62, 146.54, 140.20, 135.22, 130.15, 129.60, 128.79, 122.84, 121.67, 55.46, 48.34, 43.22.

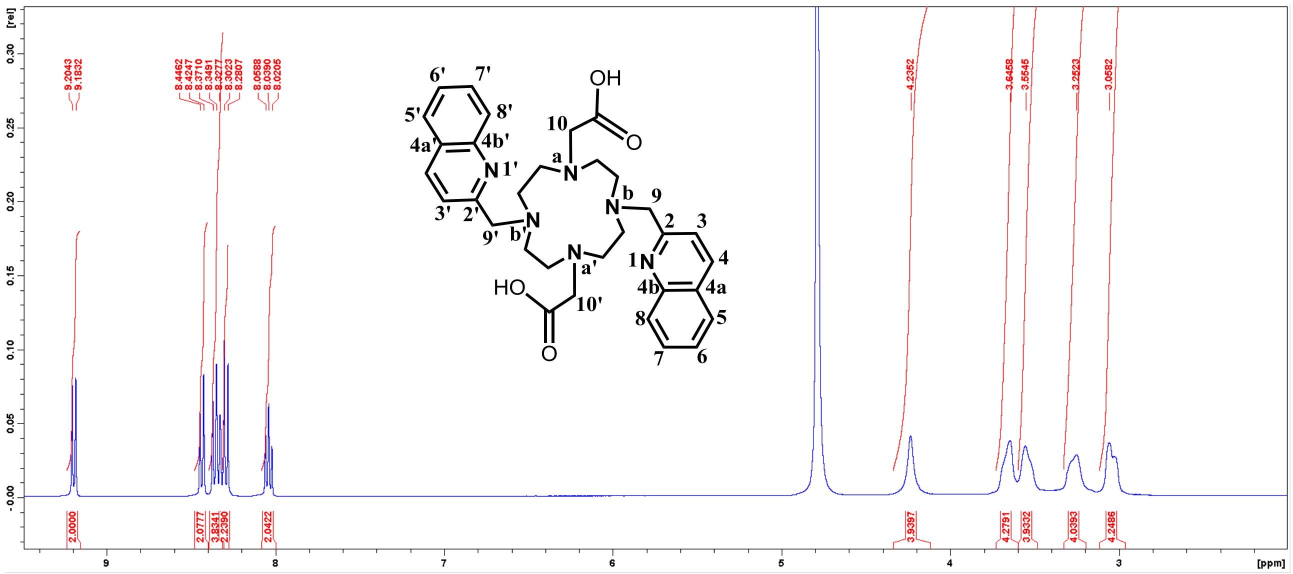

Figure S5. ^{1}H-NMR of **5** (400 MHz, D_2O, pH = 2): δ(ppm) 9.20 (d, 2H, H8,8'), 8.44 (d, 2H, H4,4'), 8.35 (m, 4H, H5,5' and H7,7'), 8.29 (d, 2H, H3,3'), δ 8.04 (t, 2H, H6,6'), 4.24 (s, 4H, H9), 3.65 ppm (m, 4H); 3.56 (m, 4H), 3.26 (m, 4H), 3.02 (m, 4H). The latter two signals are attributable to the ethylene linkers.

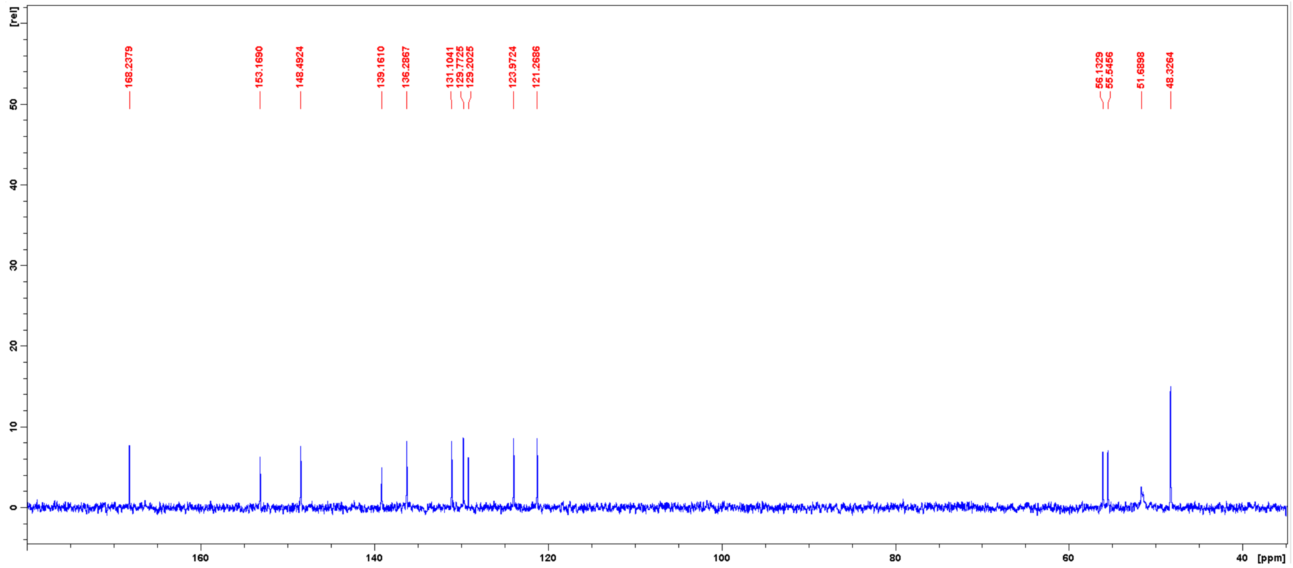

Figure S6. ^{13}C-NMR spectrum of **5** (400 MHz, D_2O, pH = 2): δ(ppm) 168.24, 153.17, 148.49, 139.16, 136.29, 131.10, 129.77, 129.20, 123.97, 121.27, 56.13, 55.55, 51.69, 48.33.

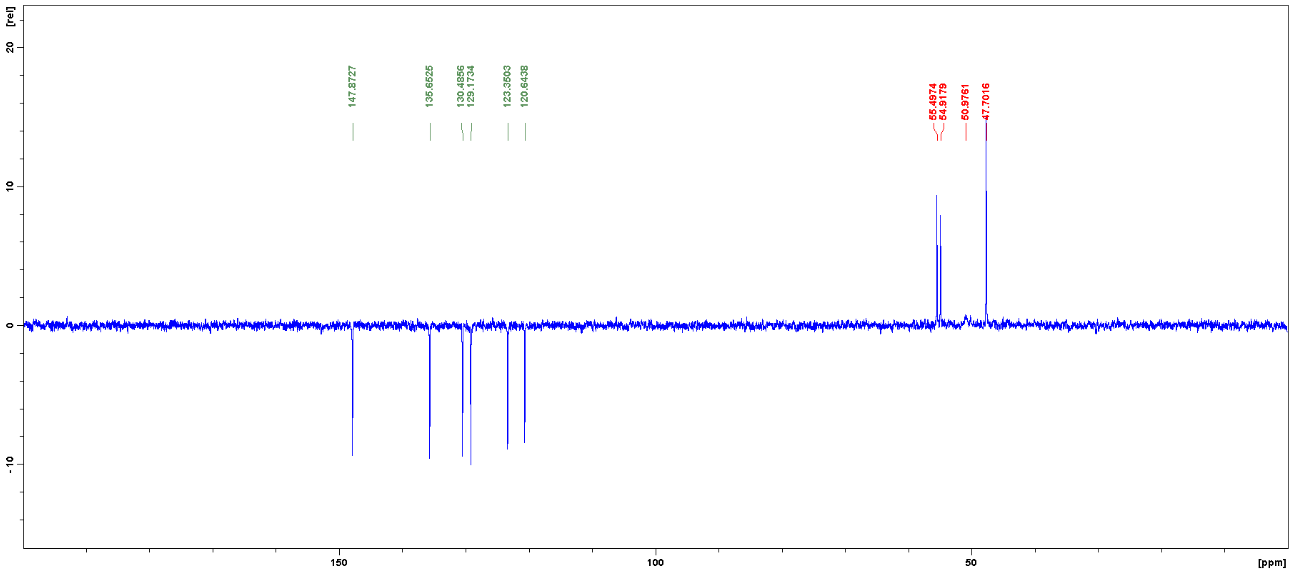

Figure S7. ^{13}C-NMR DEPT spectrum of **5** (400 MHz, D_2O, pH = 2).

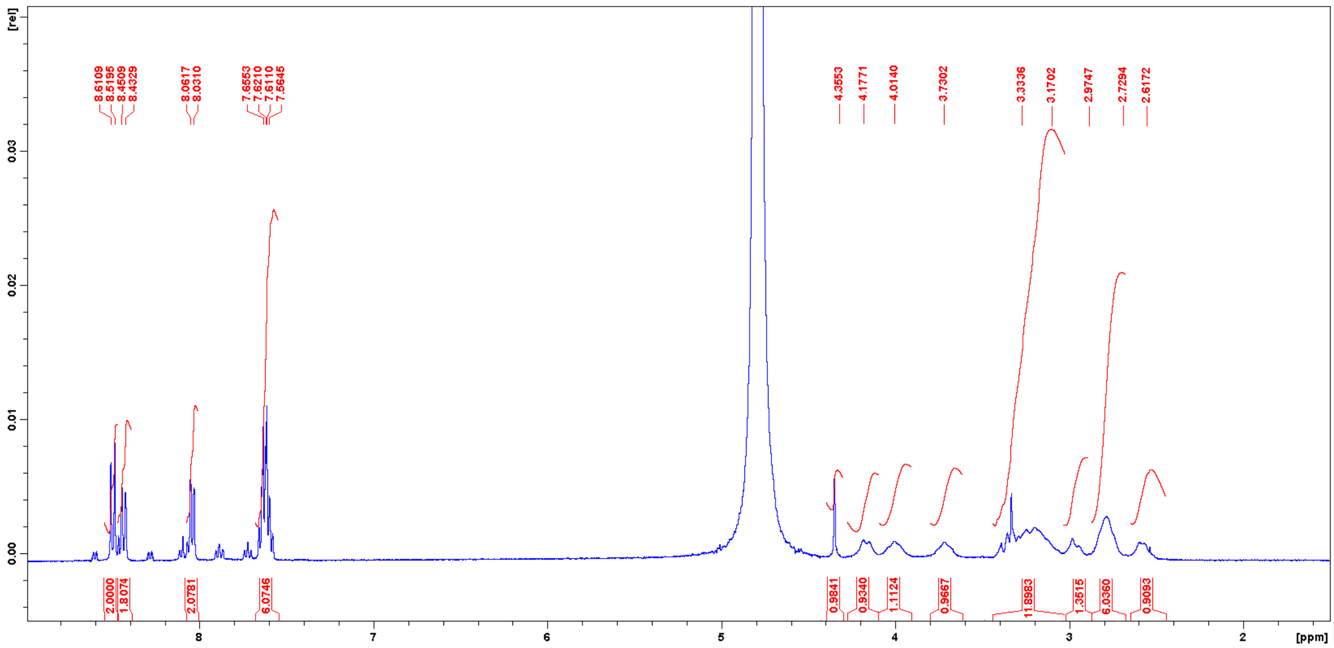

Figure S8. ^{1}H-NMR of ZnQ2 (400 MHz, D_2O, pH = 7.4): δ 8.56 (d, 2H, H8,8'), δ 8.44 (d, 2H, H4,4'), δ 8.04 (d, 2H, H5,5'), δ 7.61 (m, 6H, H3,3'+ H7,7' + H6,6'), δ 4.35 (s, 1H), δ 4.17 (m, 1H), δ 4.01 (m, 1H), δ 3.73 (m, 1H), δ 3.17 (m, 12H), δ 2.97 (m, 1H), δ 2.82 (m, 6H), δ 2.61 (m, 1H).

The signals of the aliphatic protons cannot be reliably assigned due to the broadness of the spectrum. However signal integration is as expected for the molecule.

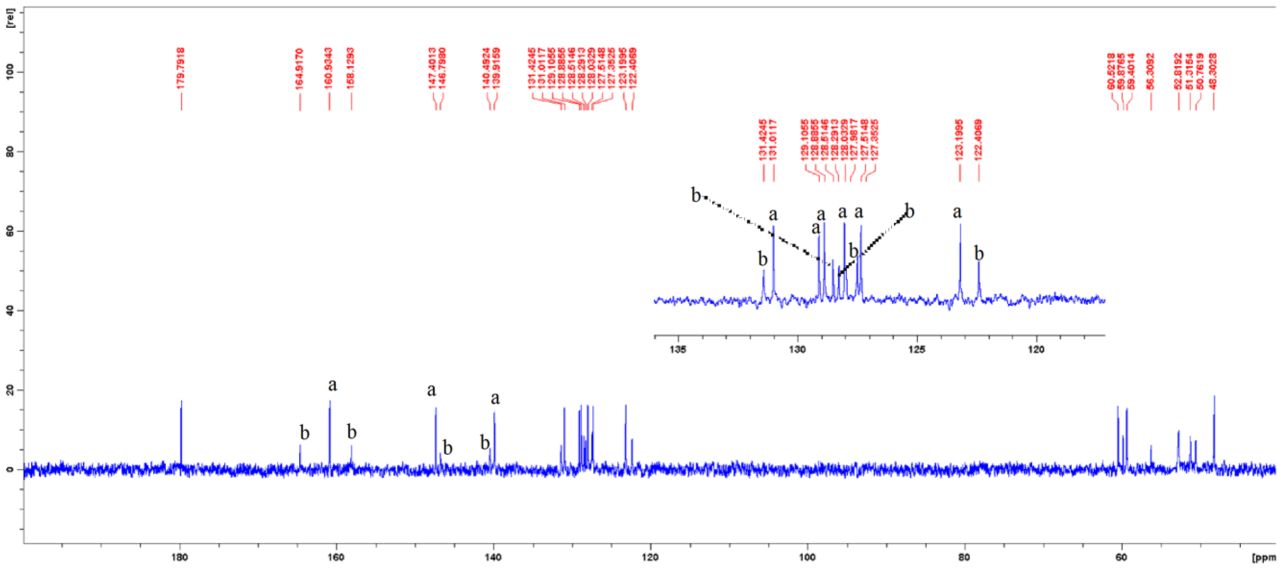

Figure S9. ^{13}C-NMR of ZnQ2 (400 MHz, D_2O, pH = 7.4): δ (ppm) 179.79, 164.91. 160.93, 158.13, 147.40, 146.79, 140.49, 139.92, 131.42, 131.01, 129.11, 128.89, 128.51, 128.29, 128.03, 127.98, 127.51, 127.35, 123.20, 122.41, 60.52, 59.88, 59.40, 56.31, 52.82, 51.32, 50.76, 48.30 ppm. Due to the scarce solubility of ZnQ2, the spectrum was recorded with a 2 x10^{-3} M complex concentration, which reduce the signal to noise ratio and the overall quality of the spectrum. The letters a and b indicate the two different subspectra observed in the aromatic region for the quinoline units. The signal at 179.79 pmm is due to carboxylate groups.

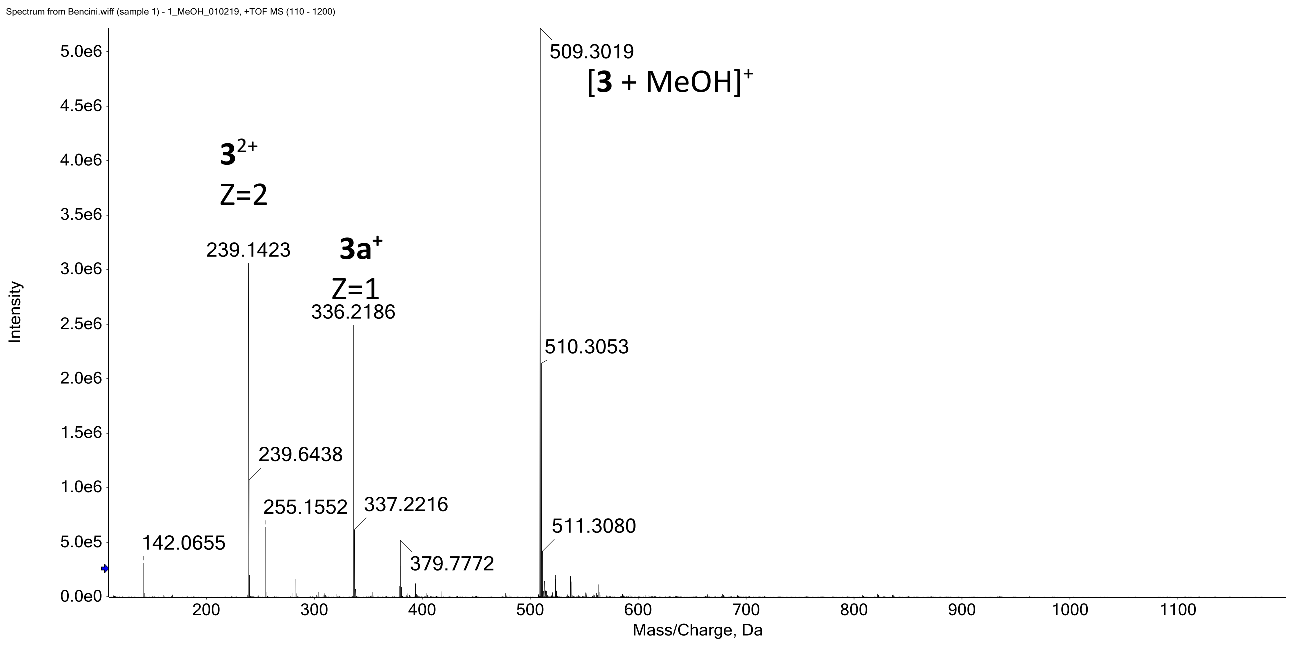

Figure S10. High resolution mass spectrum of compound **3** in MeOH. The fragment **3a**$^+$ derives from the lost of a methylenquinoline unit of **3** (see Figure S13).

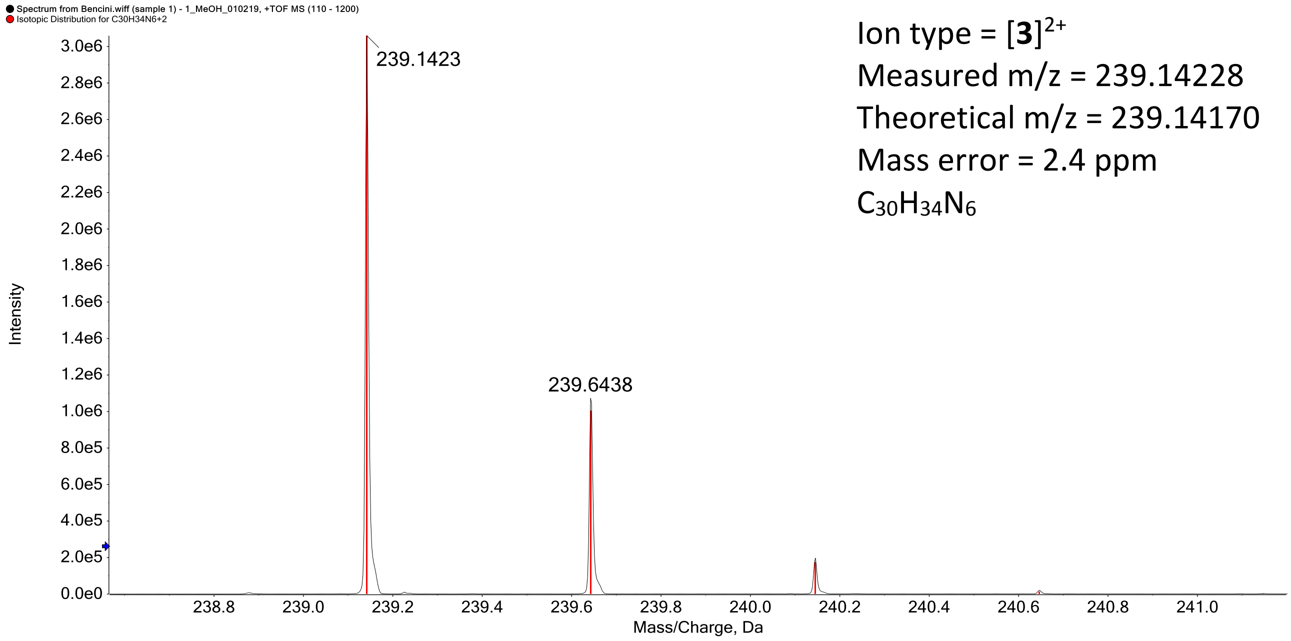

Figure S11. Isotopic pattern of the **3**$^{2+}$ (Z =2) ion, with measured and theoretical m/z value of the most abundant isotopic peak.

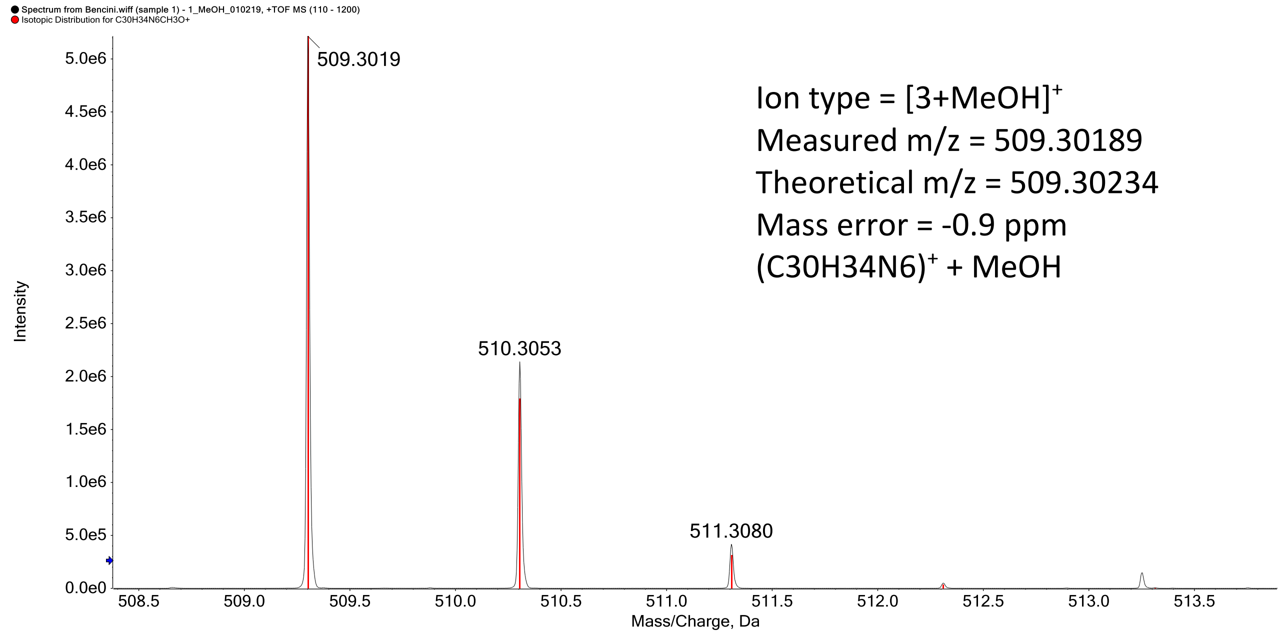

Figure S12. Isotopic pattern of the [**3**+MeOH]$^+$ (Z =1) adduct, with measured and theoretical m/z value of the most abundant isotopic peak.

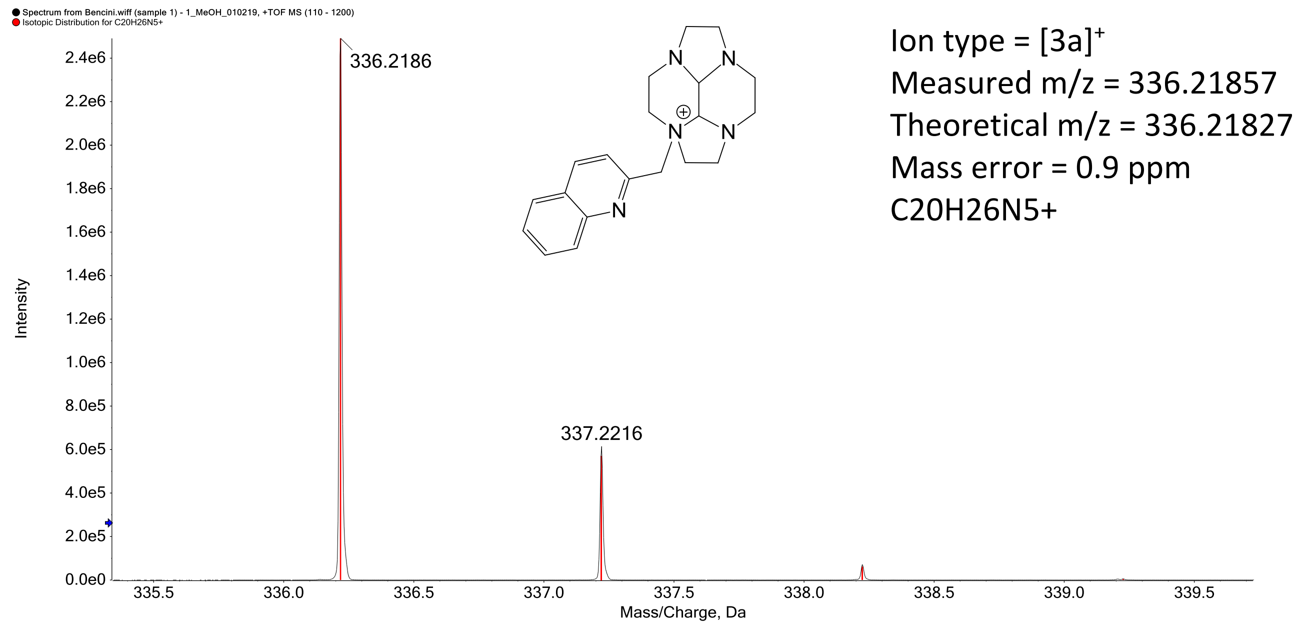

Figure S13. Isotopic pattern of the [**3a**]$^+$ (Z =1) fragment, with measured and theoretical m/z values of the most abundant isotopic peak. The fragment **3a**$^+$ derives from the lost of a methylenquinoline unit of **3**.

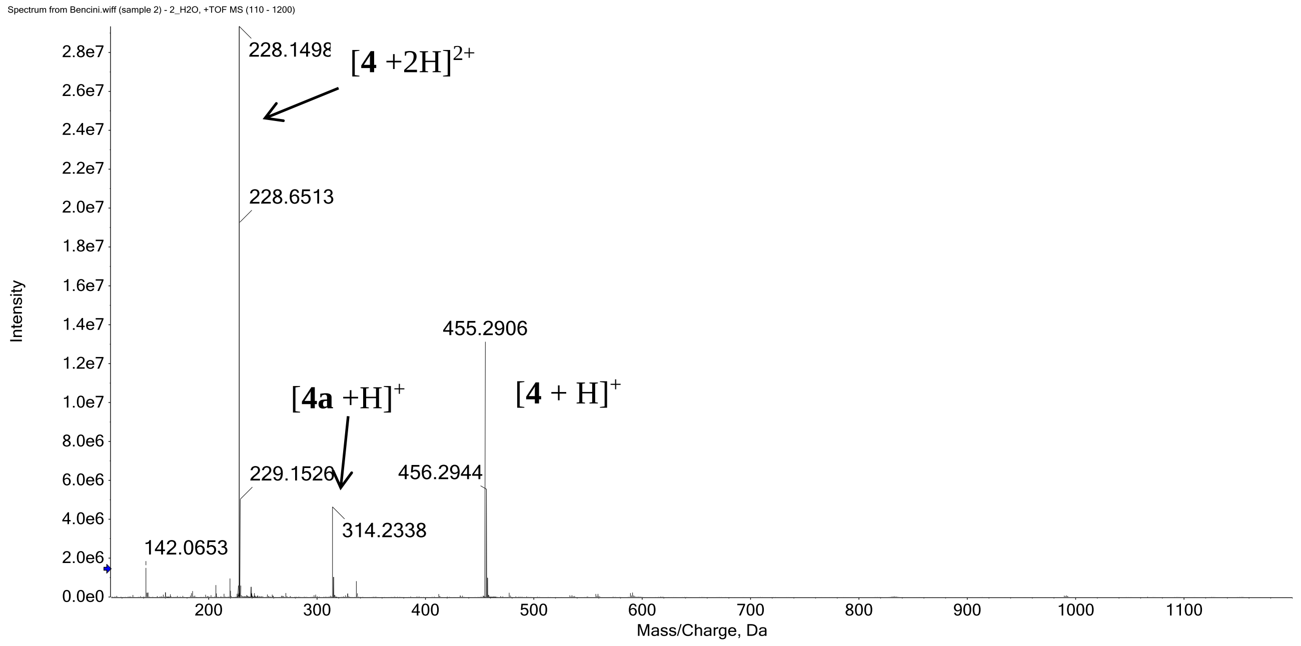

Figure S14. High resolution mass spectrum of compound **4** in H_2O. The fragment **4a** derives from the lost of one methylenquinoline unit of **4** (see Figure S17).

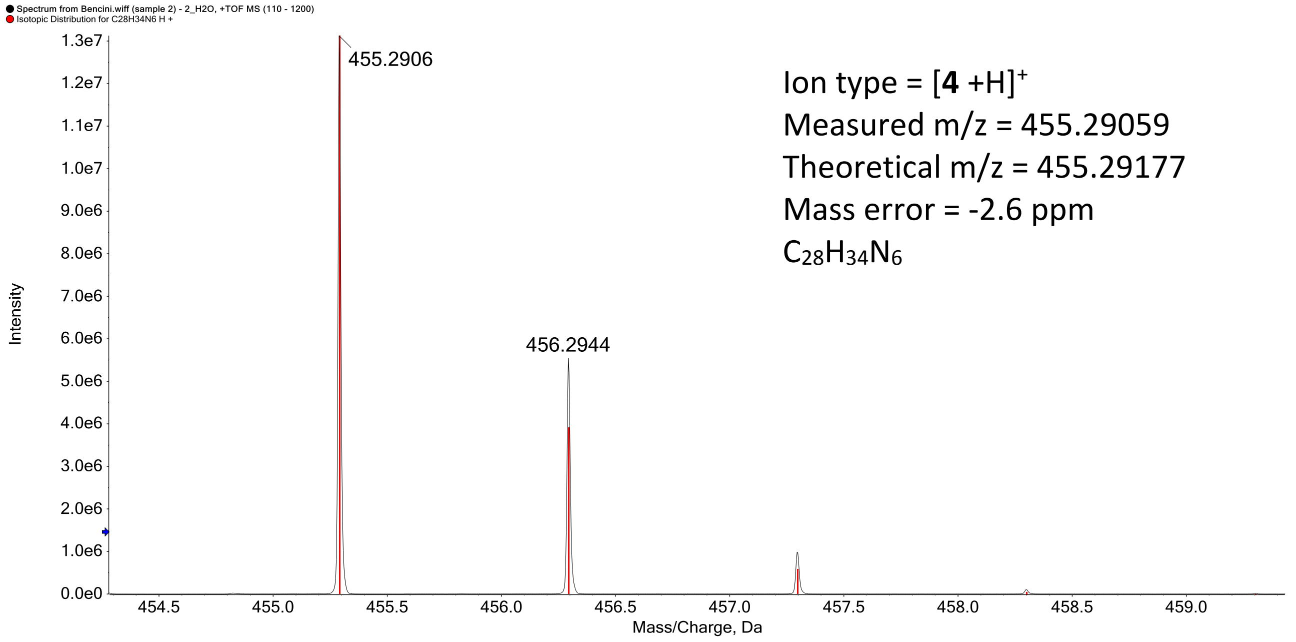

Figure S15. Isotopic pattern of the [**4** + H]$^+$ (Z =1) ion, with measured and theoretical m/z value of the most abundant isotopic peak.

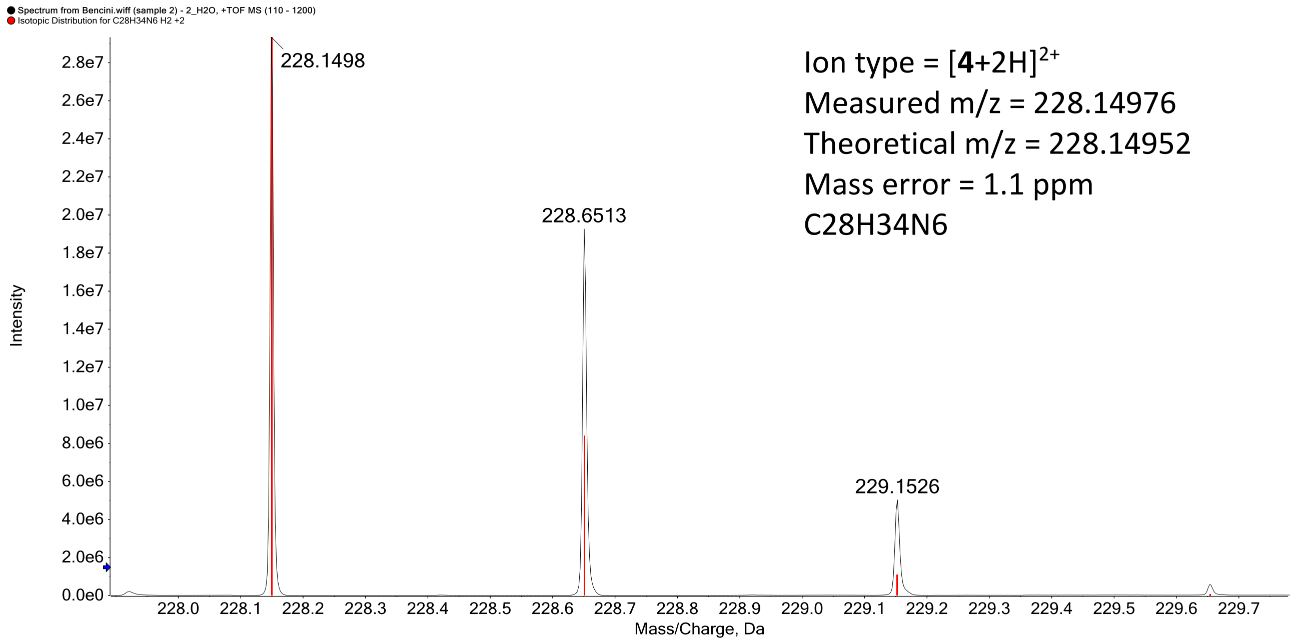

Figure S16. Isotopic pattern of the [**4** + 2H]$^{2+}$ (Z =2) ion, with measured and theoretical m/z value of the most abundant isotopic peak.

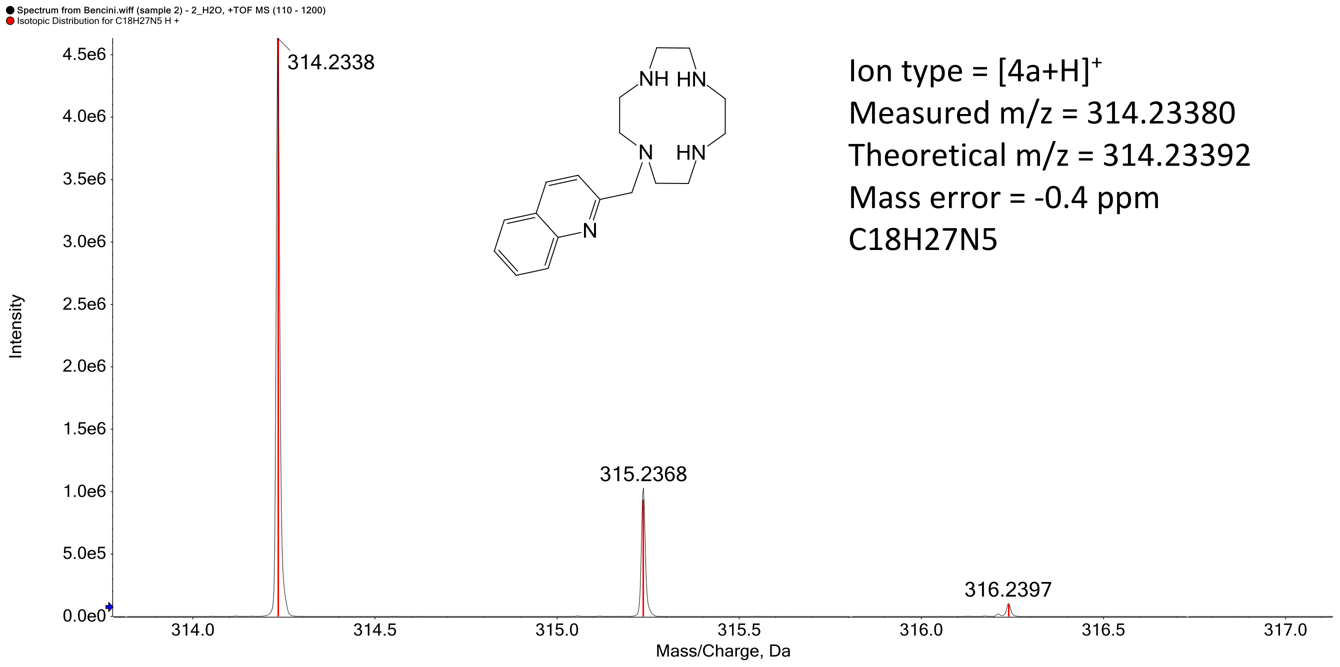

Figure S17. Isotopic pattern of the [**4a** + H]$^{+}$ (Z =1) ion, with measured and theoretical m/z value of the most abundant isotopic peak. The fragment **4a** derives from the lost of a methylenquinoline unit of **4**.

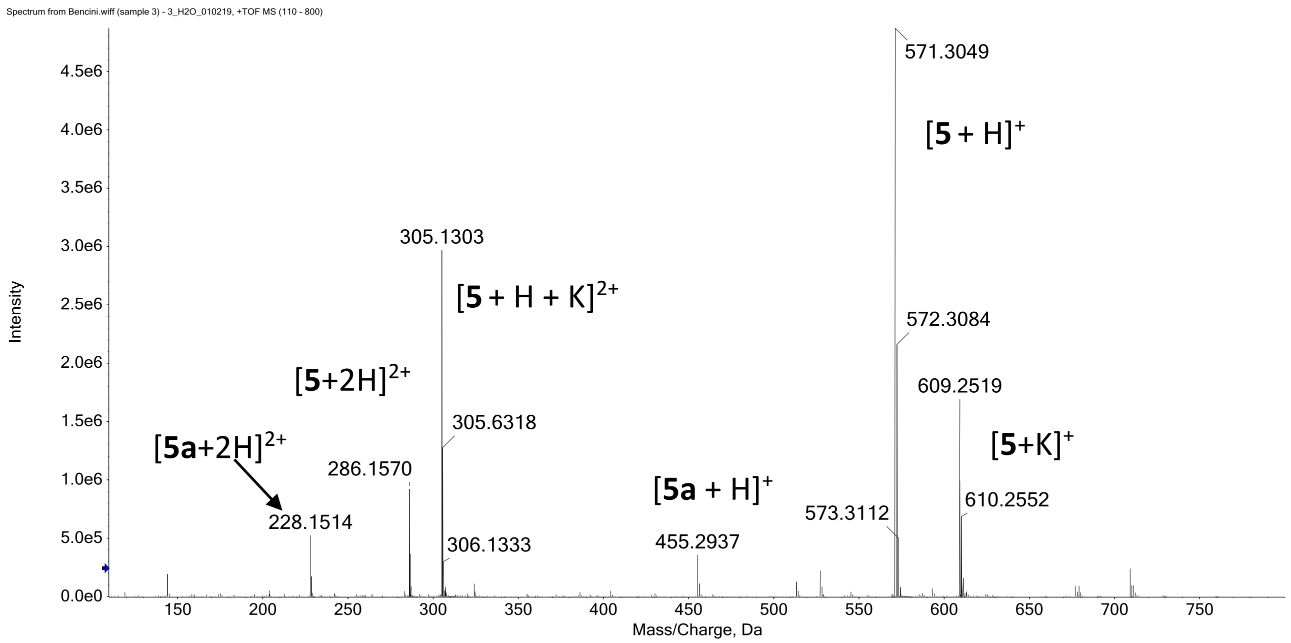

Figure S18. High resolution mass spectrum of compound **5** in H_2O. **5a** derives from the lost of the two methylencarboxylic acid groups of **5** (see Figure S23 and S24).

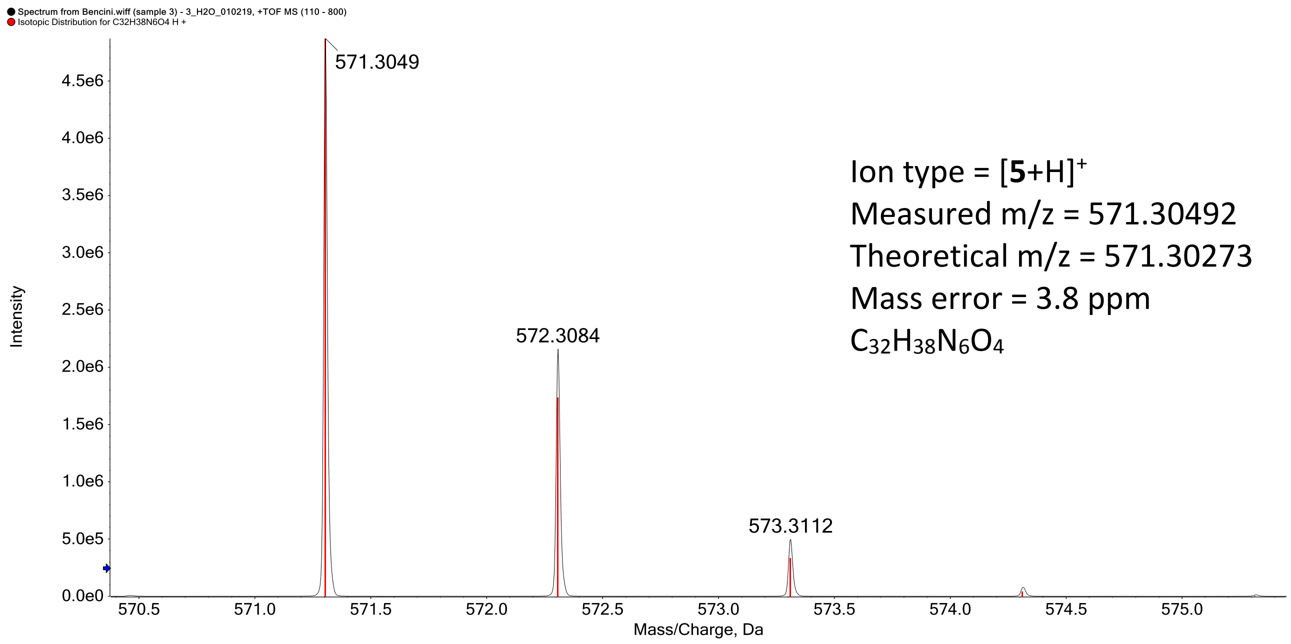

Figure S19. Isotopic pattern of the [**5** + H]$^+$ (Z =1) ion, with measured and theoretical m/z value of the most abundant isotopic peak.

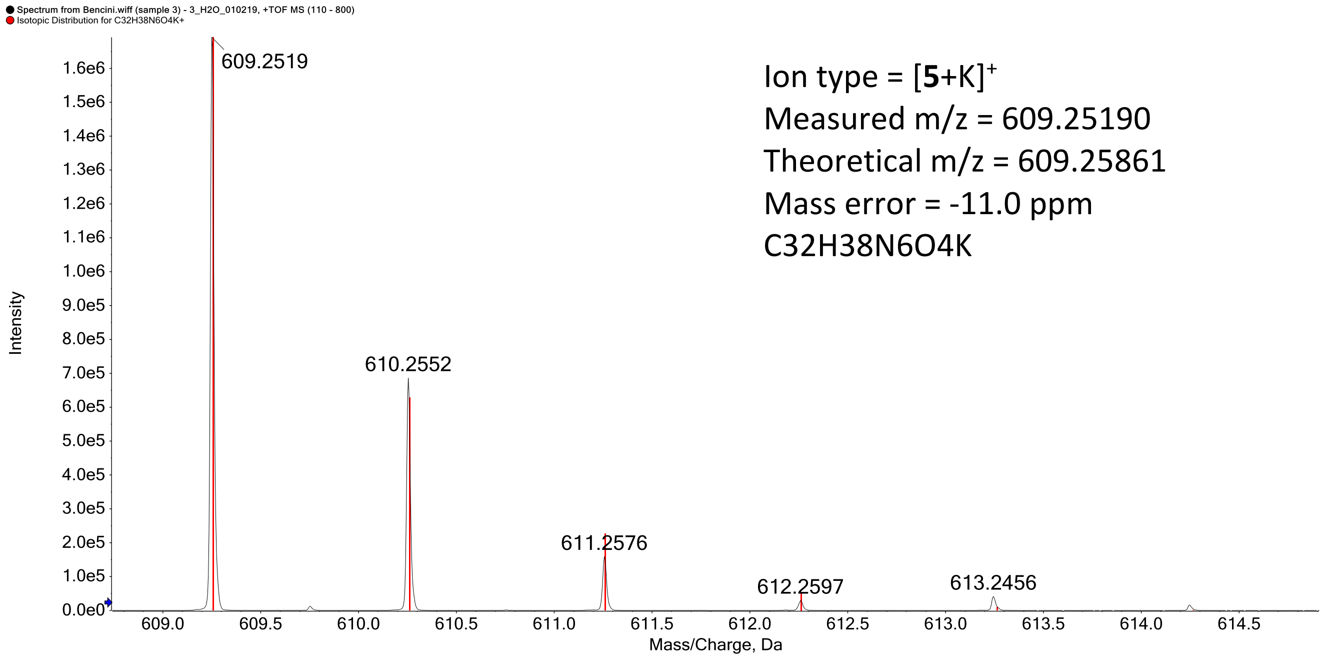

Figure S20. Isotopic pattern of the [**5** + K]$^{+}$ (Z =1) adduct, with measured and theoretical m/z value of the most abundant isotopic peak.

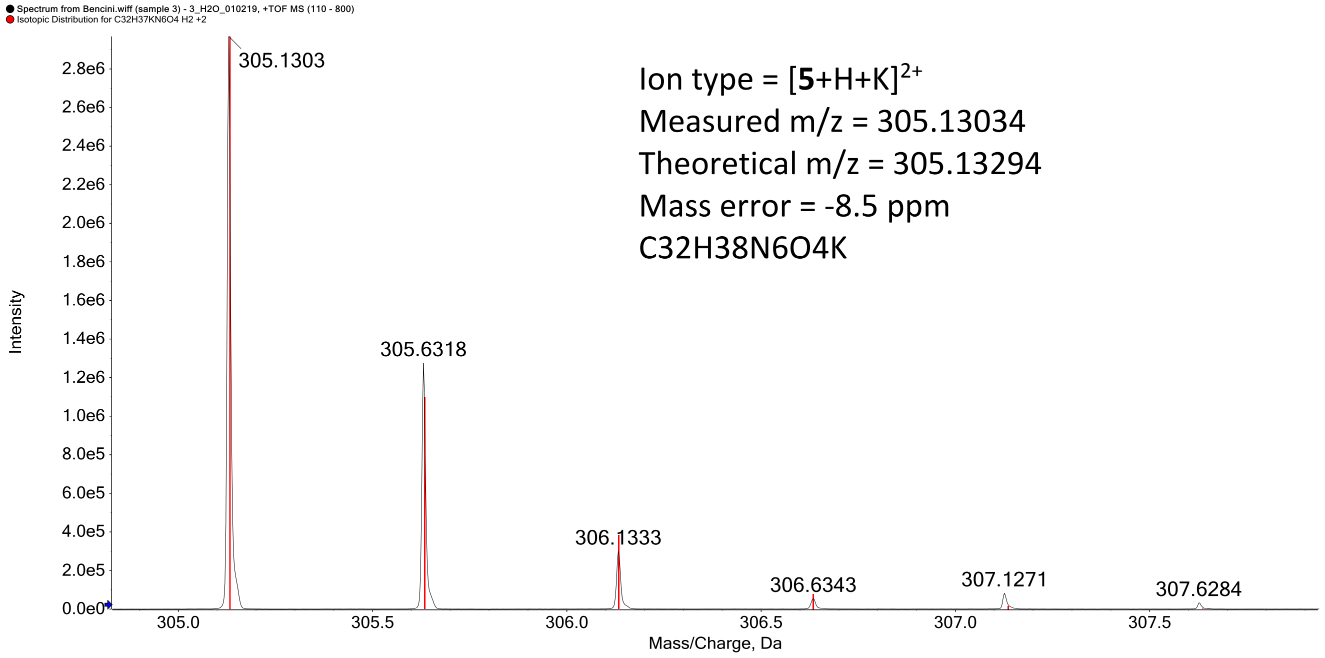

Figure S21. Isotopic pattern of the [**5** + H + K]$^{2+}$ (Z = 2) ion, with measured and theoretical m/z value of the most abundant isotopic peak.

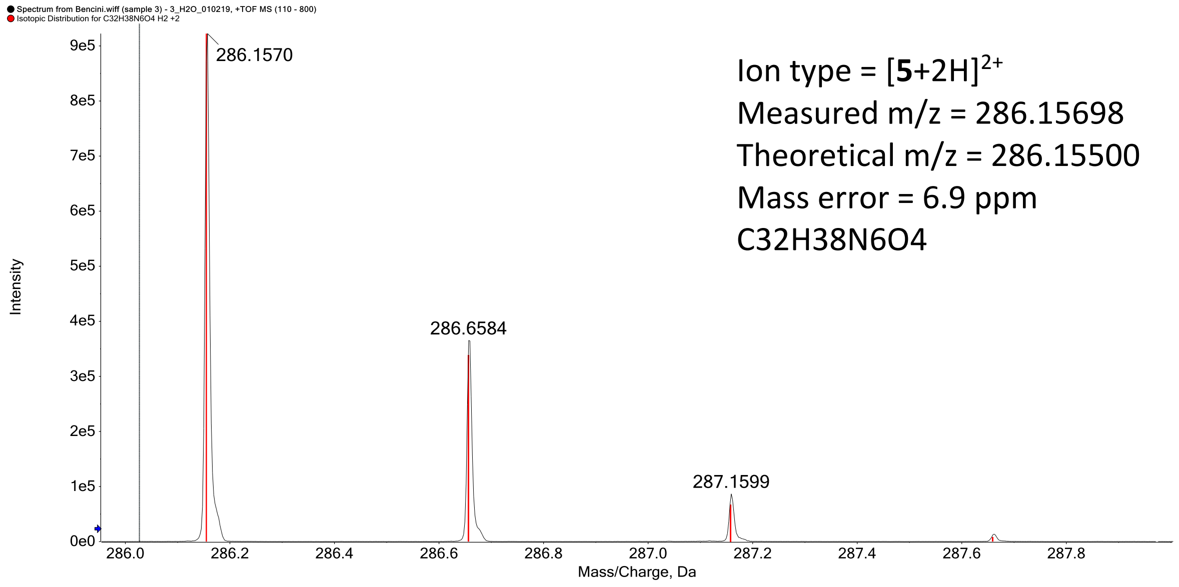

Figure S22. Isotopic pattern of the $[\mathbf{5} + 2H]^{2+}$ (Z = 2) ion, with measured and theoretical m/z value of the most abundant isotopic peak.

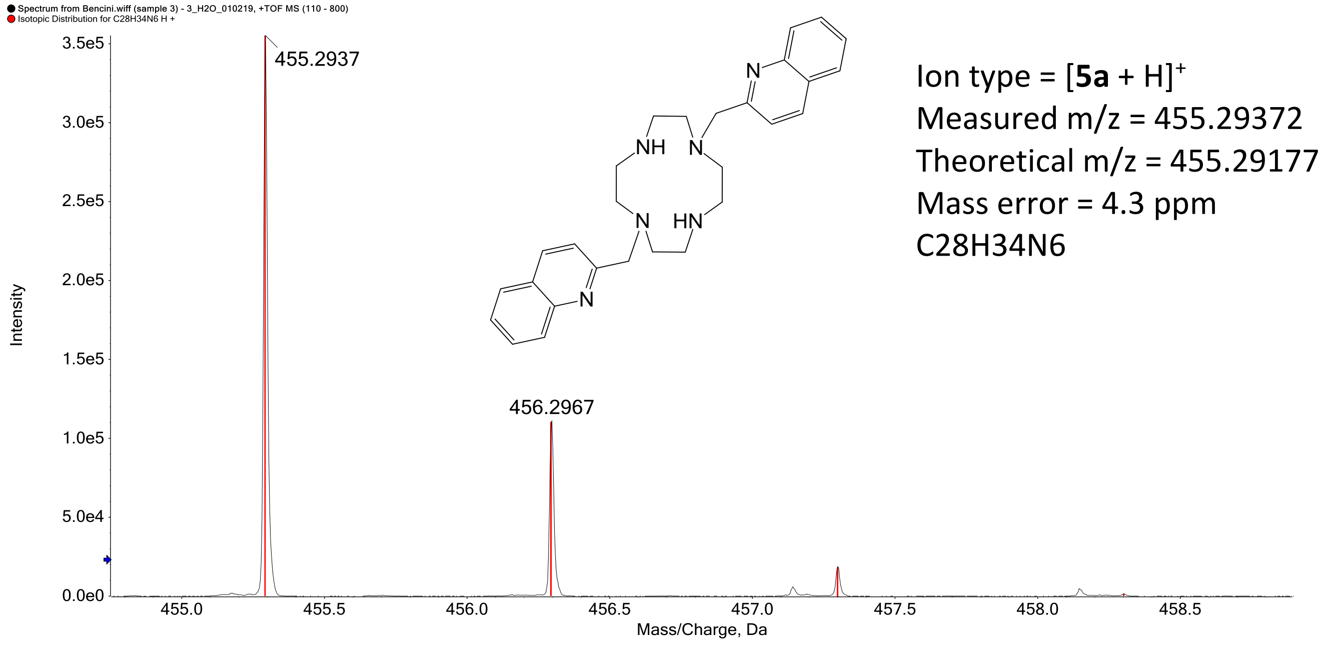

Figure S23. Isotopic pattern of the $[\mathbf{5a} + H]^{+}$ (Z = 1) peak, with measured and theoretical m/z value of the most abundant isotopic peak. **5a** derives from the lost of the two methylencarboxylic acid groups of **5**.

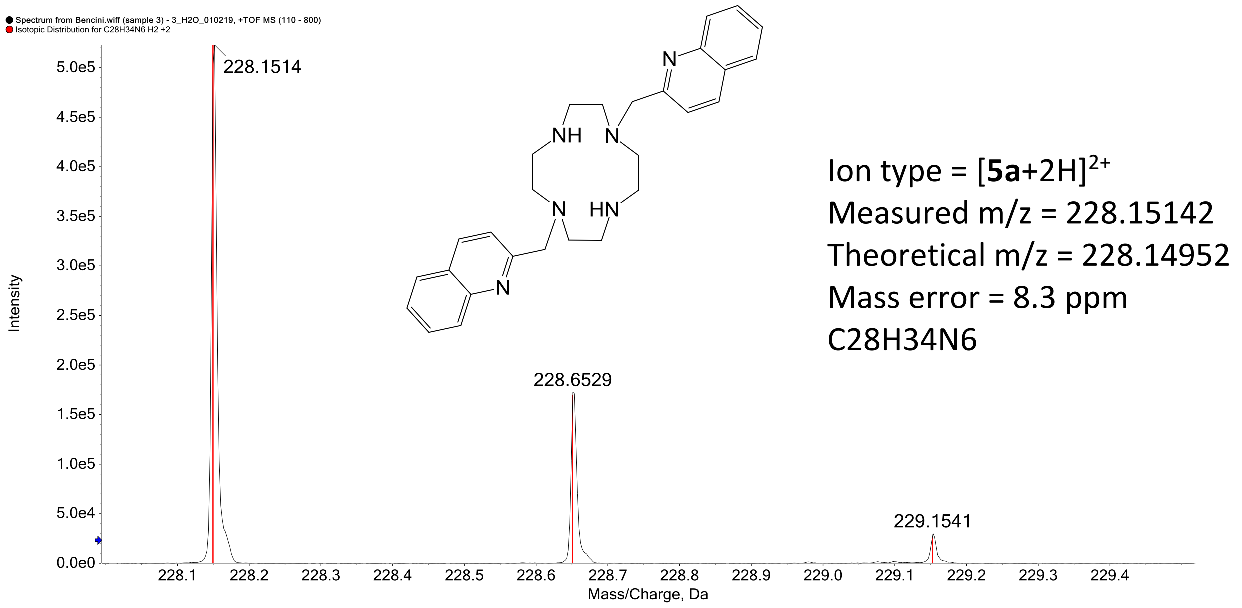

Figure S24. Isotopic pattern of the [**5a** + 2H]$^{2+}$ (Z = 2) peak, with measured and theoretical m/z value of the most abundant isotopic peak. Compound **5a** derives from the lost of the two methylencarboxylic acid groups of **5**.

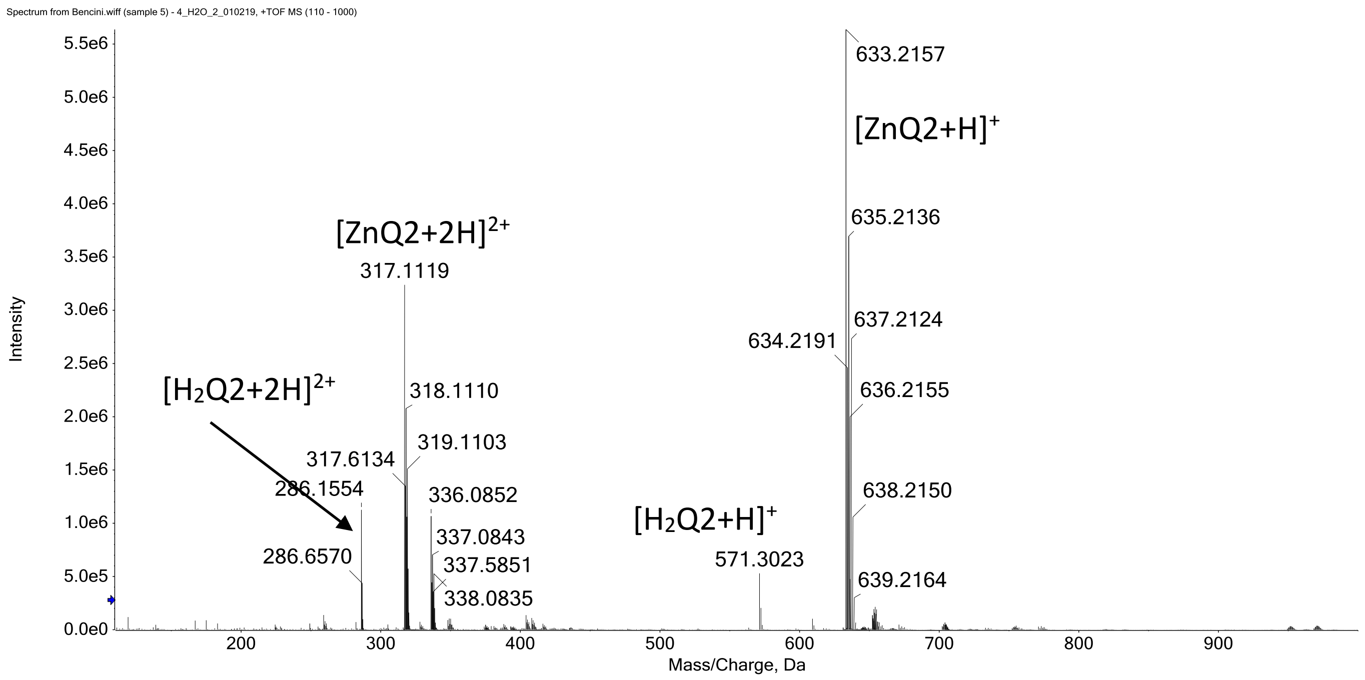

Figure S25. High resolution mass spectrum of complex ZnQ2 in H_2O.

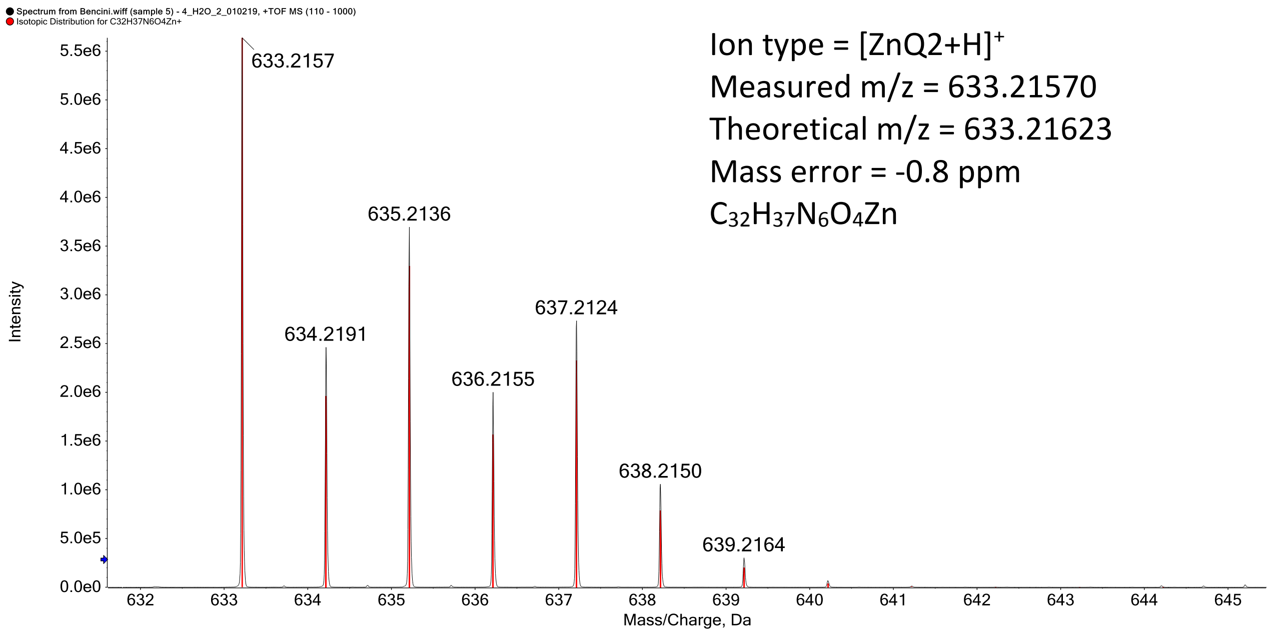

Figure S26. Isotopic pattern of the $[ZnQ2 + H]^+$ (Z =1) ion, with measured and theoretical m/z value for the most abundant peak.

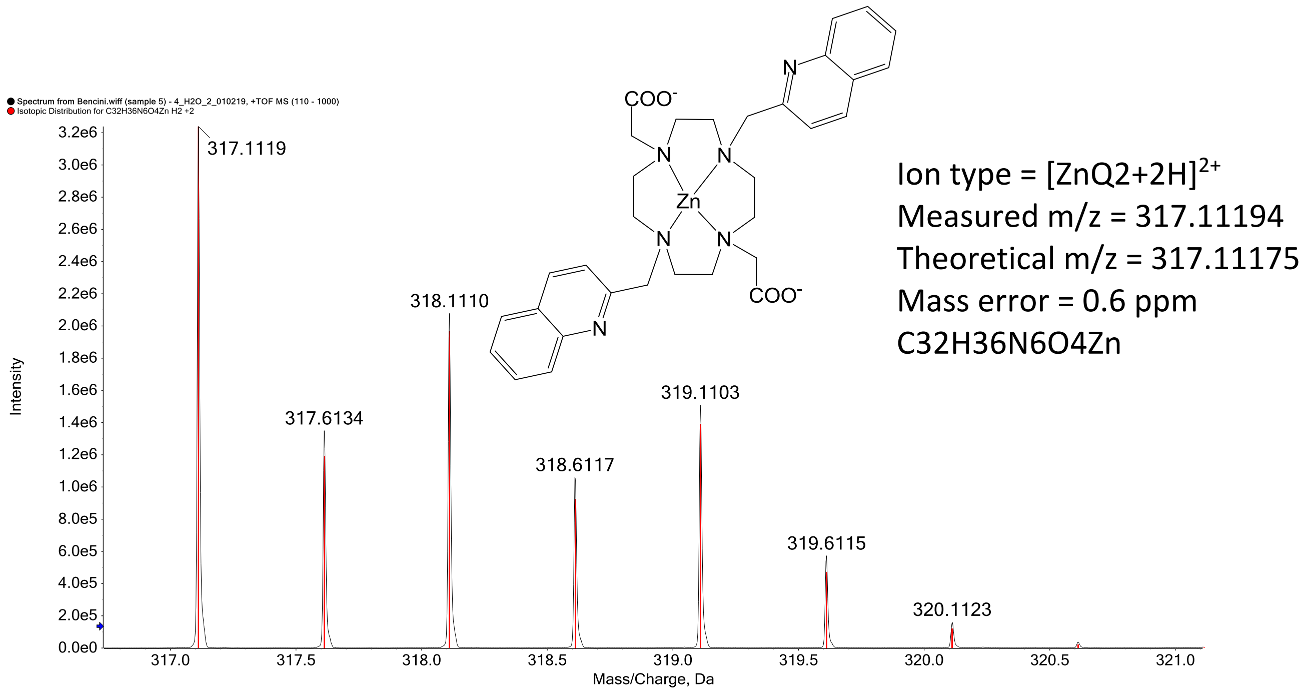

Figure S27. Isotopic pattern of the $[ZnQ2 + 2H]^{2+}$ (Z =2) ion, with measured and theoretical m/z value for the most abundant peak.

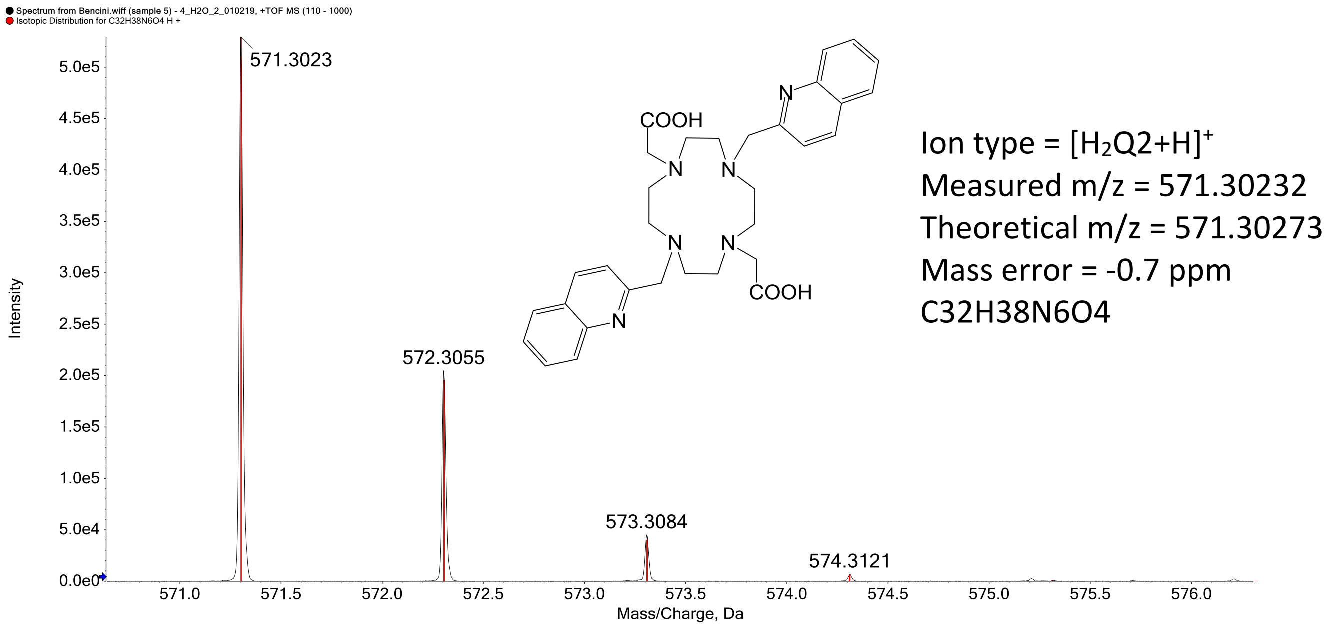

Figure S28. Isotopic pattern of the $[H_2Q2 + H]^+$ (Z = 1) ion, with measured and theoretical m/z value for the most abundant one. H_2Q2 derives from demetallation of the Zn^{2+} complex.

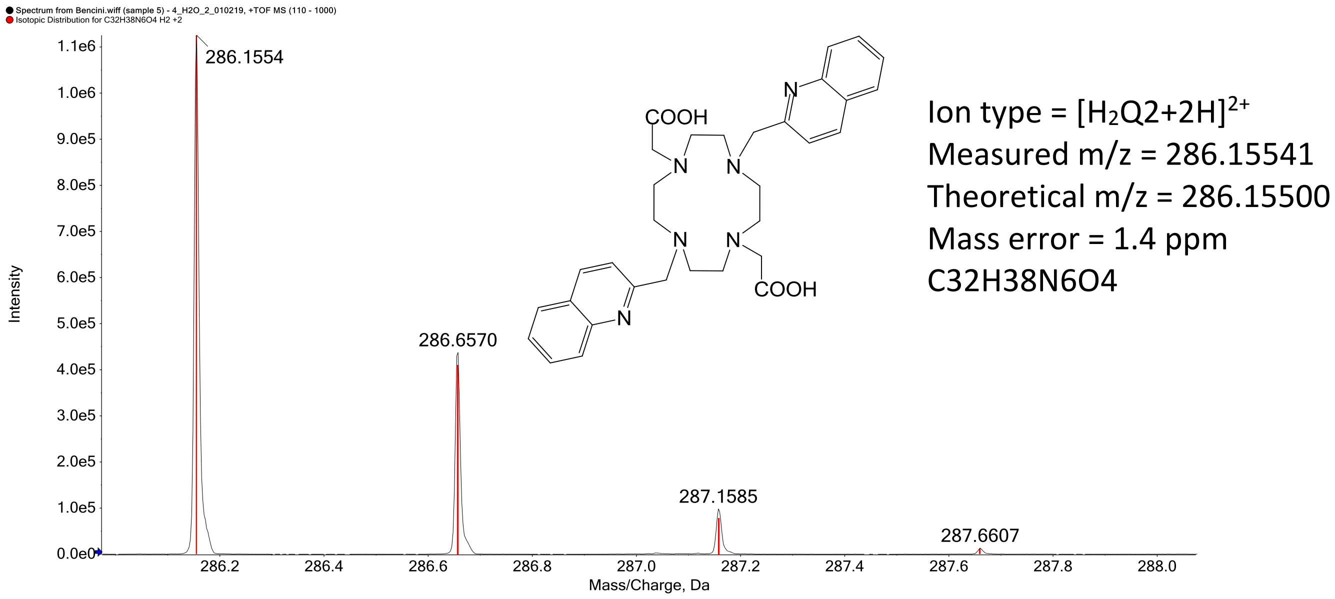

Figure S29. Isotopic pattern of the $[H_2Q2 + 2H]^{2+}$ (Z = 1) ion, with measured and theoretical m/z value for the most abundant one. H_2Q2 derives from demetallation of the Zn^{2+} complex.

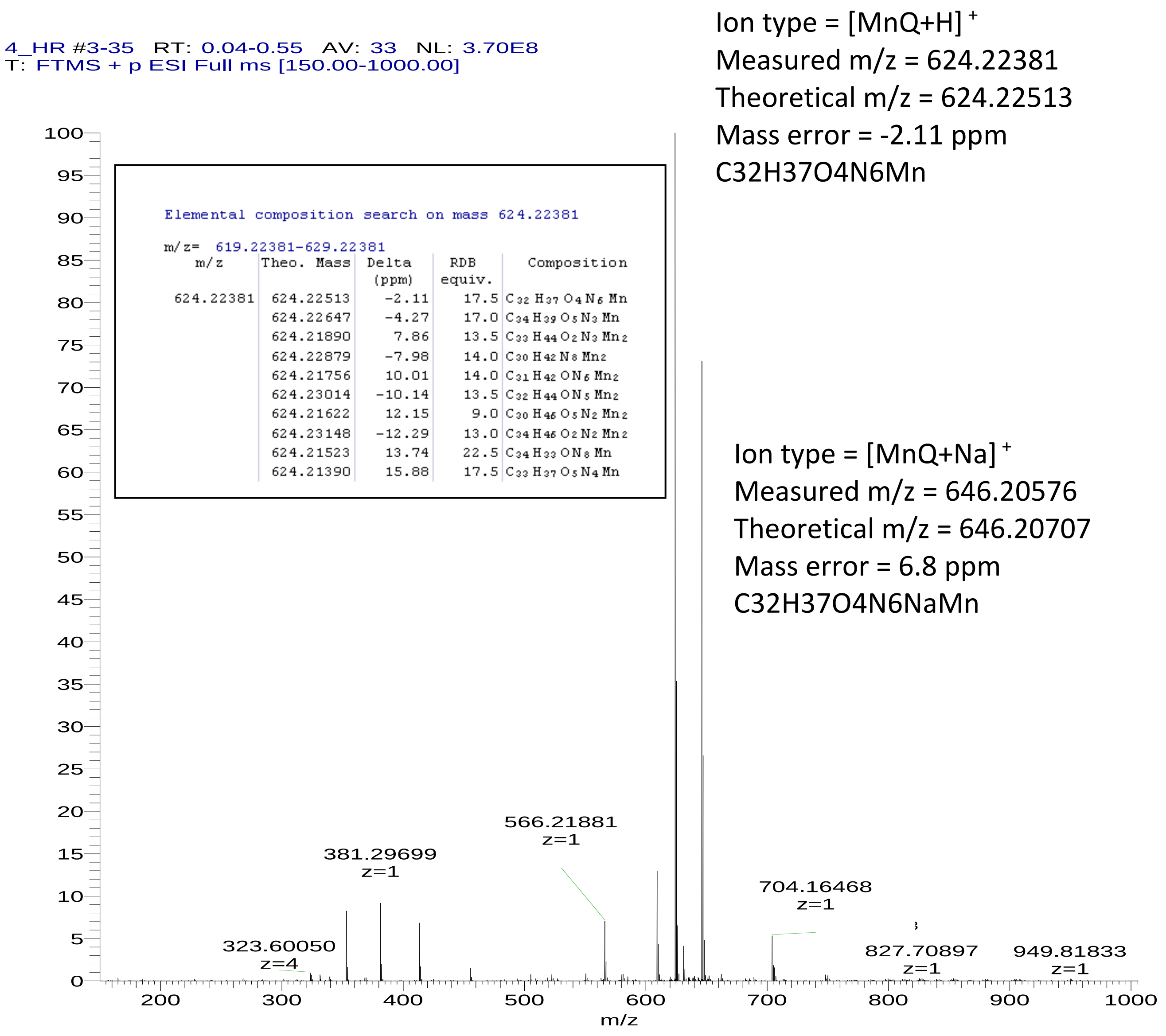

m/z	Theo. Mass	Delta (ppm)	RDB equiv.	Composition
624.22381	624.22513	-2.11	17.5	$C_{32}H_{37}O_4N_6Mn$
	624.22647	-4.27	17.0	$C_{34}H_{39}O_5N_3Mn$
	624.21890	7.86	13.5	$C_{33}H_{44}O_2N_3Mn_2$
	624.22879	-7.98	14.0	$C_{30}H_{42}N_8Mn_2$
	624.21756	10.01	14.0	$C_{31}H_{42}ON_6Mn_2$
	624.23014	-10.14	13.5	$C_{32}H_{44}ON_5Mn_2$
	624.21622	12.15	9.0	$C_{30}H_{46}O_5N_2Mn_2$
	624.23148	-12.29	13.0	$C_{34}H_{46}O_2N_2Mn_2$
	624.21523	13.74	22.5	$C_{34}H_{33}ON_8Mn$
	624.21390	15.88	17.5	$C_{33}H_{37}O_5N_4Mn$

Figure S30. High resolution mass spectrum of complex MnQ2 in H_2O, with measured and theoretical m/z values of the reliably attributable peaks.

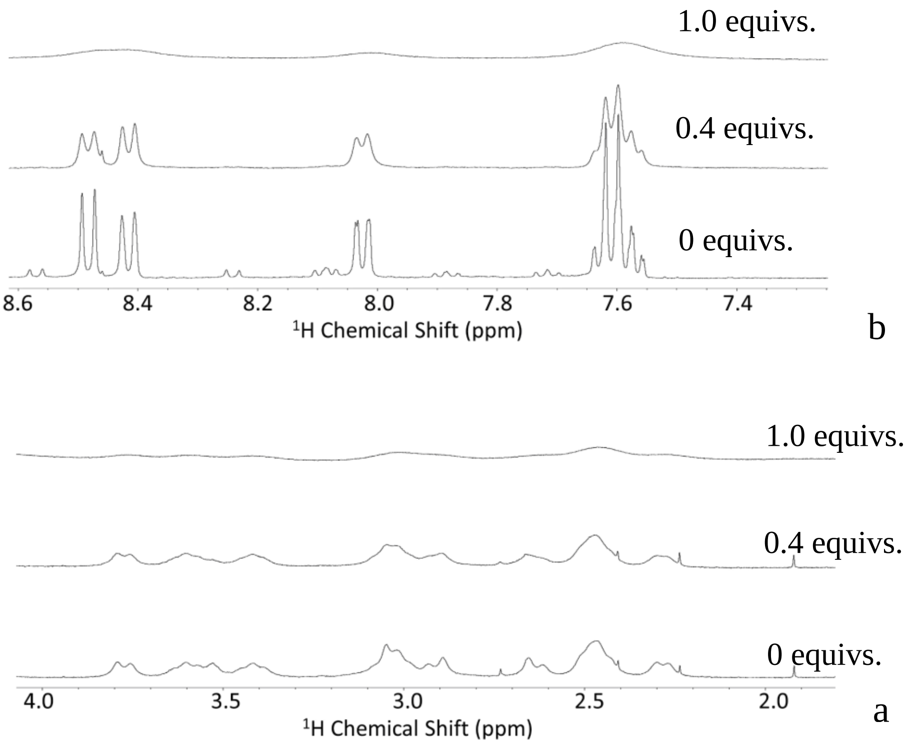

Figure S31. ^{1}H NMR spectra of H_2Q2 collected at 400 MHz (a: aliphatic section, b: aromatic section) at pH 7.4 in the absence and in the presence of 0.4 and 1.0 equivalents of Mn^{II}.

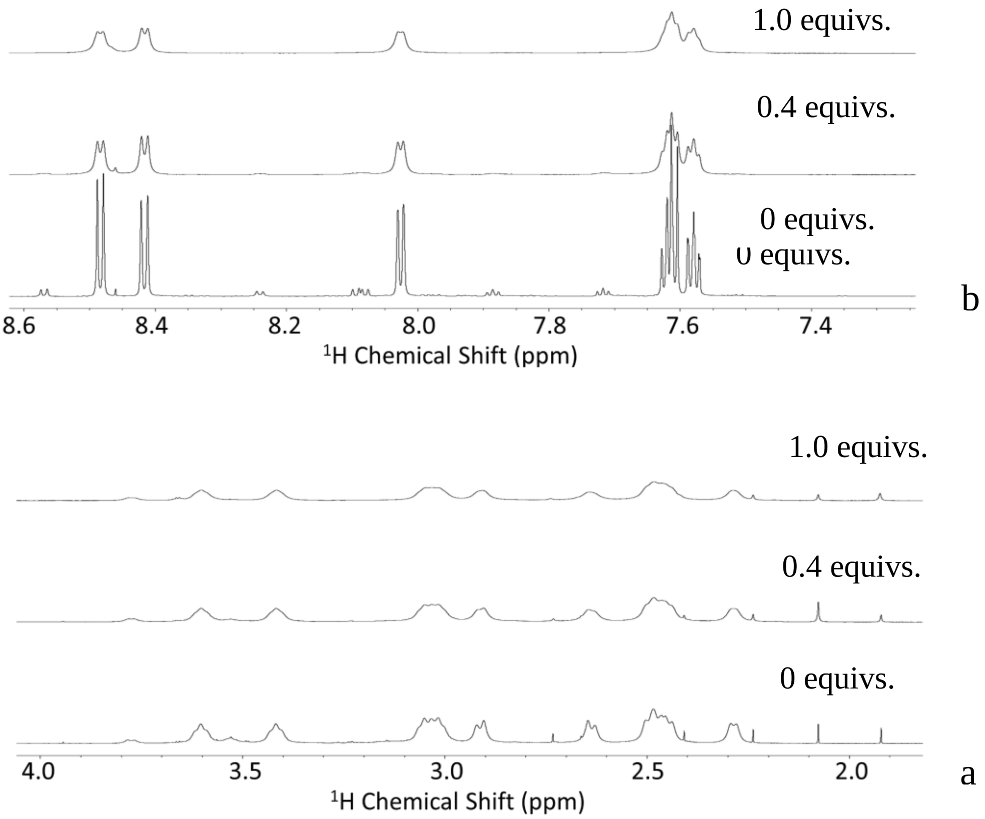

Figure S32. ^{1}H NMR spectra of H_2Q2 collected at 900 MHz (a: aliphatic section, b: aromatic section) at pH 7.4 and 298 K in the absence and in the presence of 0.4 and 1.0 equivalents of Mn^{II}.

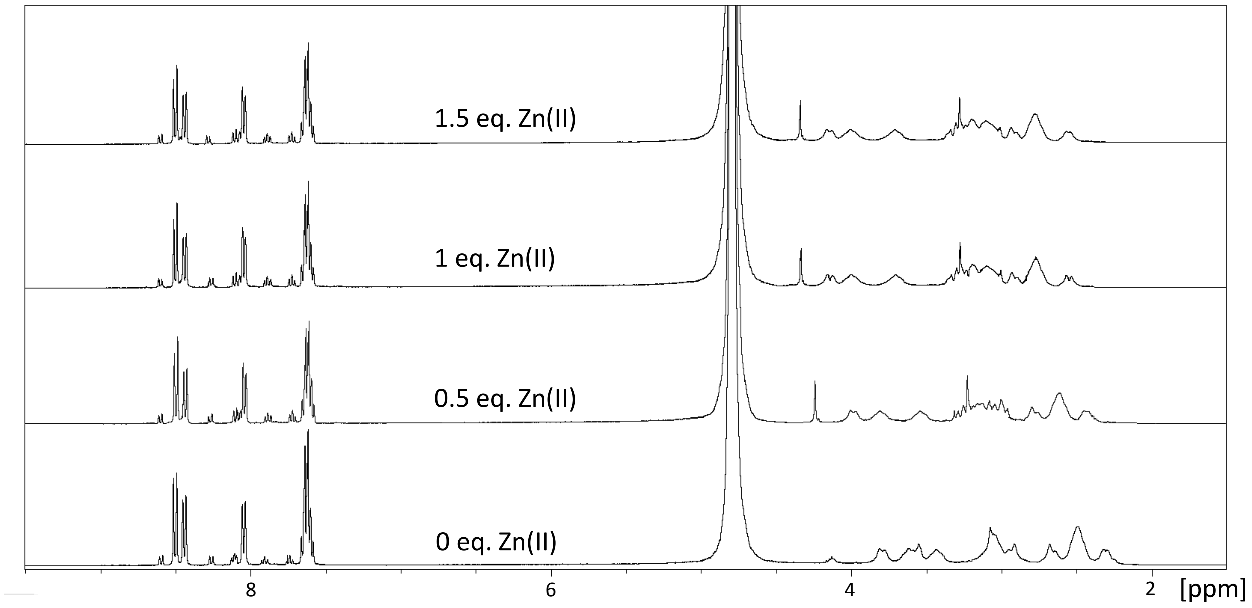

Figure S33. ^{1}H NMR spectra of H_2Q2 collected at 900 MHz (a: aliphatic section, b: aromatic section) at pD 7.4 and 298 K in the absence and in the presence of 0.5, 1.0 and 1.5 equivalents of Zn^{II}.

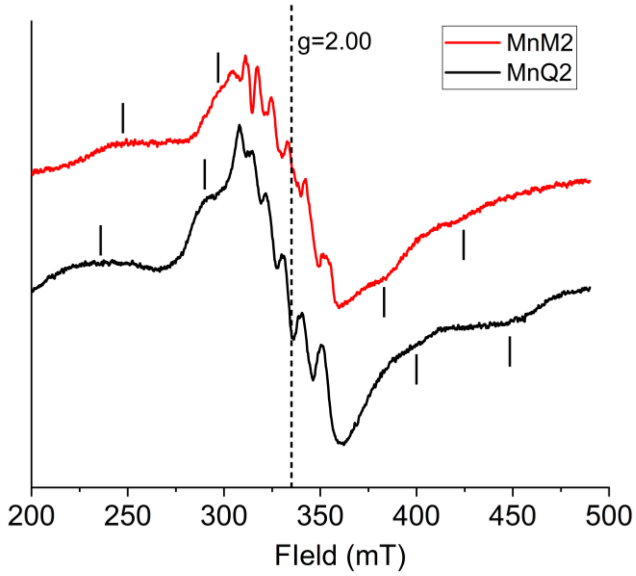

Figure S34. X-band (ν =9.40 GHz) EPR spectra of MnM2 and MnQ2 recorded in frozen solution at 100 K. The ticks evidence the resonance fields of the broader transition between levels with larger m_S values due to the ZFS of the two molecules (see text for details). The dashed line highlights the resonance filed corresponding to g=2.00

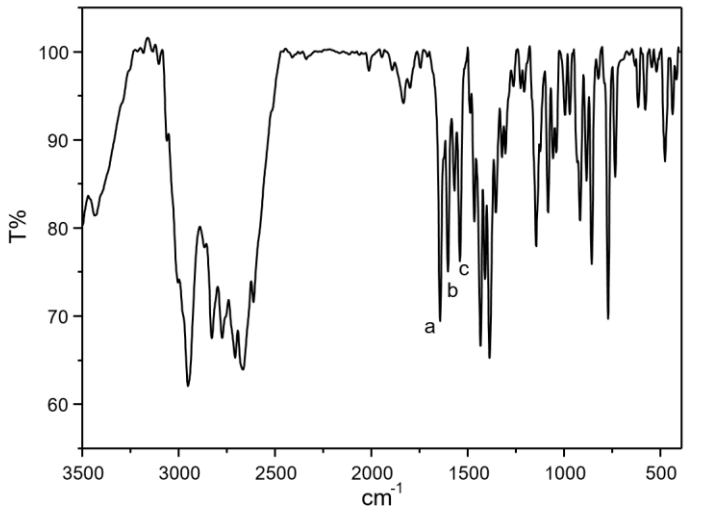

a

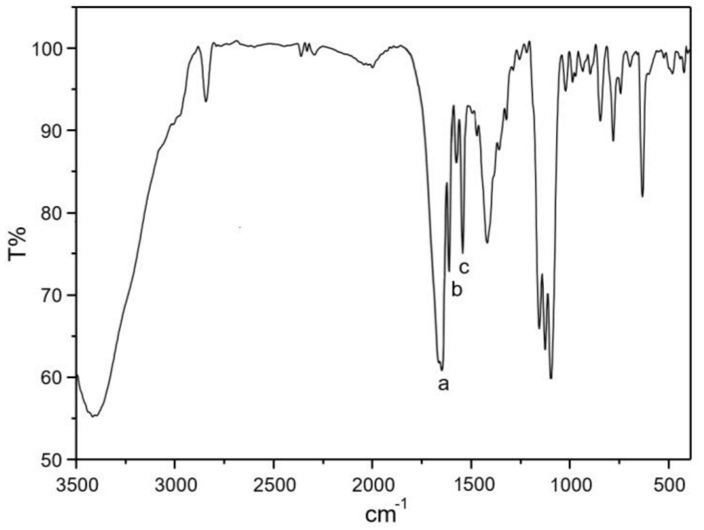

b

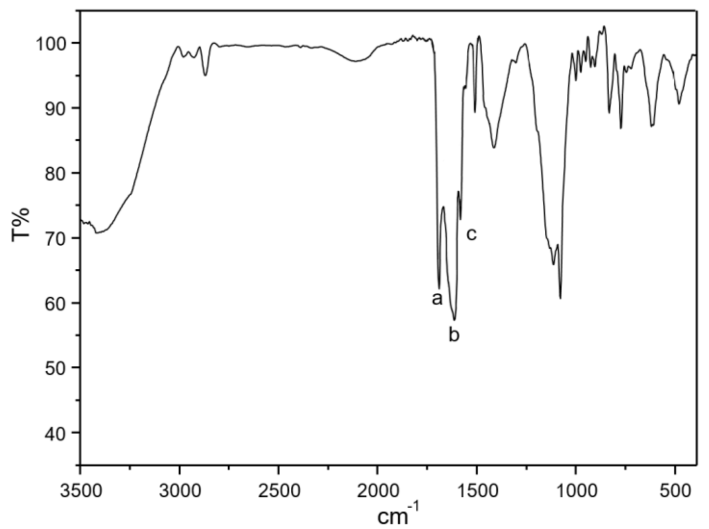

c

Figure S35. IR spectra of compond **4** (a), H_2Q2 (5)(b) and MnQ2 (c)
