# Supplementary material for: Different Antioxidant Efficacy of Two MnII-Containing Superoxide Anion Scavengers on Hypoxia/Reoxygenation-Exposed Cardiac Muscle Cells
Source: Sci Rep. 2019 Jul 16;9:10320. doi: 10.1038/s41598-019-46476-2 (PMC6635543; doi:10.1038/s41598-019-46476-2)
Supplement: Supplementary file 2 — Supplementary information 2 [file 41598_2019_46476_MOESM2_ESM.pdf]

DIFFERENT ANTIOXIDANT EFFICACY OF TWO Mn^{II}-CONTAINING SUPEROXIDE ANION SCAVENGERS ON HYPOXIA/REOXYGENATION-EXPOSED CARDIAC MUSCLE CELLS.

Matteo Becatti, Andrea Bencini, Silvia Nistri, Luca Conti, Maria Giulia Fabbrini, Laura Lucarini, Veronica Ghini, Mirko Severi, Claudia Fiorillo, Claudia Giorgi, Lorenzo Sorace, Barbara Valtancoli, Daniele Bani

Supplementary information 2 - Catalysis of $O_2^{\bullet -}$ dismutation by cytochrome *c* assay.

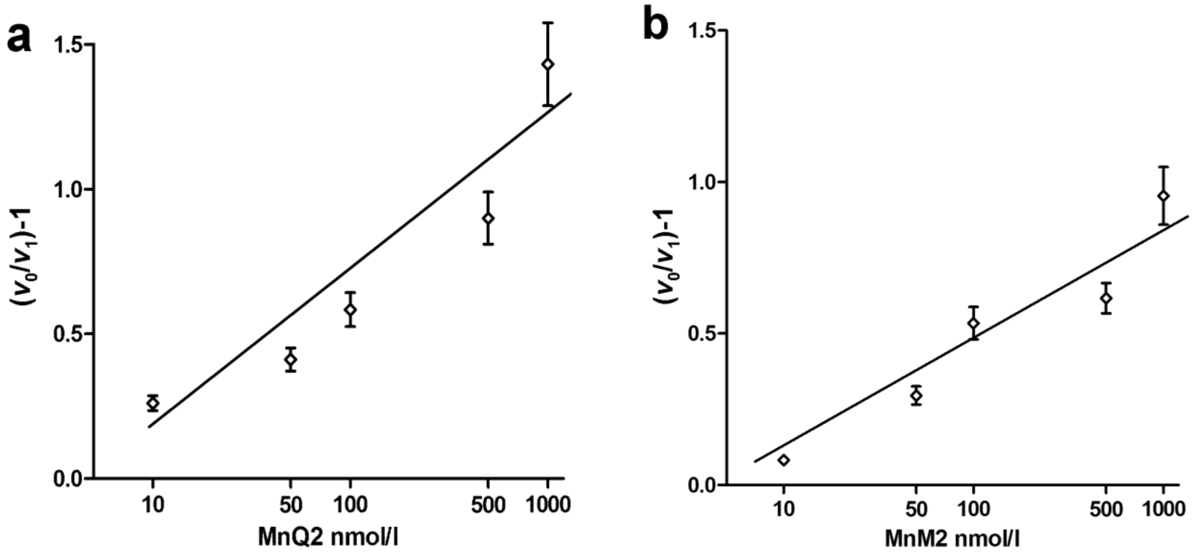

Plots of $\{(v_0/v_1) - 1\}$ vs. **a**) MnQ2, **b**) MnM2. v_0 is the rate of reduction of 10 μM cytochrome *c* by $O_2^{\bullet -}$ and v_1 the rate of reduction of cytochome *c* inhibited by the compound in the presence of 0.1 mM EDTA in 0.05 M Tris buffer at pH 7.8, 40 μM xanthine, ~2 nM xanthine oxidase at 25°C. The total volume of the assay solution is 1 mL.
