# Supplementary material for: Different Antioxidant Efficacy of Two MnII-Containing Superoxide Anion Scavengers on Hypoxia/Reoxygenation-Exposed Cardiac Muscle Cells
Source: Sci Rep. 2019 Jul 16;9:10320. doi: 10.1038/s41598-019-46476-2 (PMC6635543; doi:10.1038/s41598-019-46476-2)

DIFFERENT ANTIOXIDANT EFFICACY OF TWO Mn^{II}-CONTAINING SUPEROXIDE ANION SCAVENGERS ON HYPOXIA/REOXYGENATION-EXPOSED CARDIAC MUSCLE CELLS.

Matteo Becatti, Andrea Bencini, Silvia Nistri, Luca Conti, Maria Giulia Fabbrini, Laura Lucarini, Veronica Ghini, Mirko Severi, Claudia Fiorillo, Claudia Giorgi, Lorenzo Sorace, Barbara Valtancoli, Daniele Bani

Supplementary information 3 - Representative FACS diagrams of H9c2 cells at the noted experimental conditions, assayed after 1 h reoxygenation.

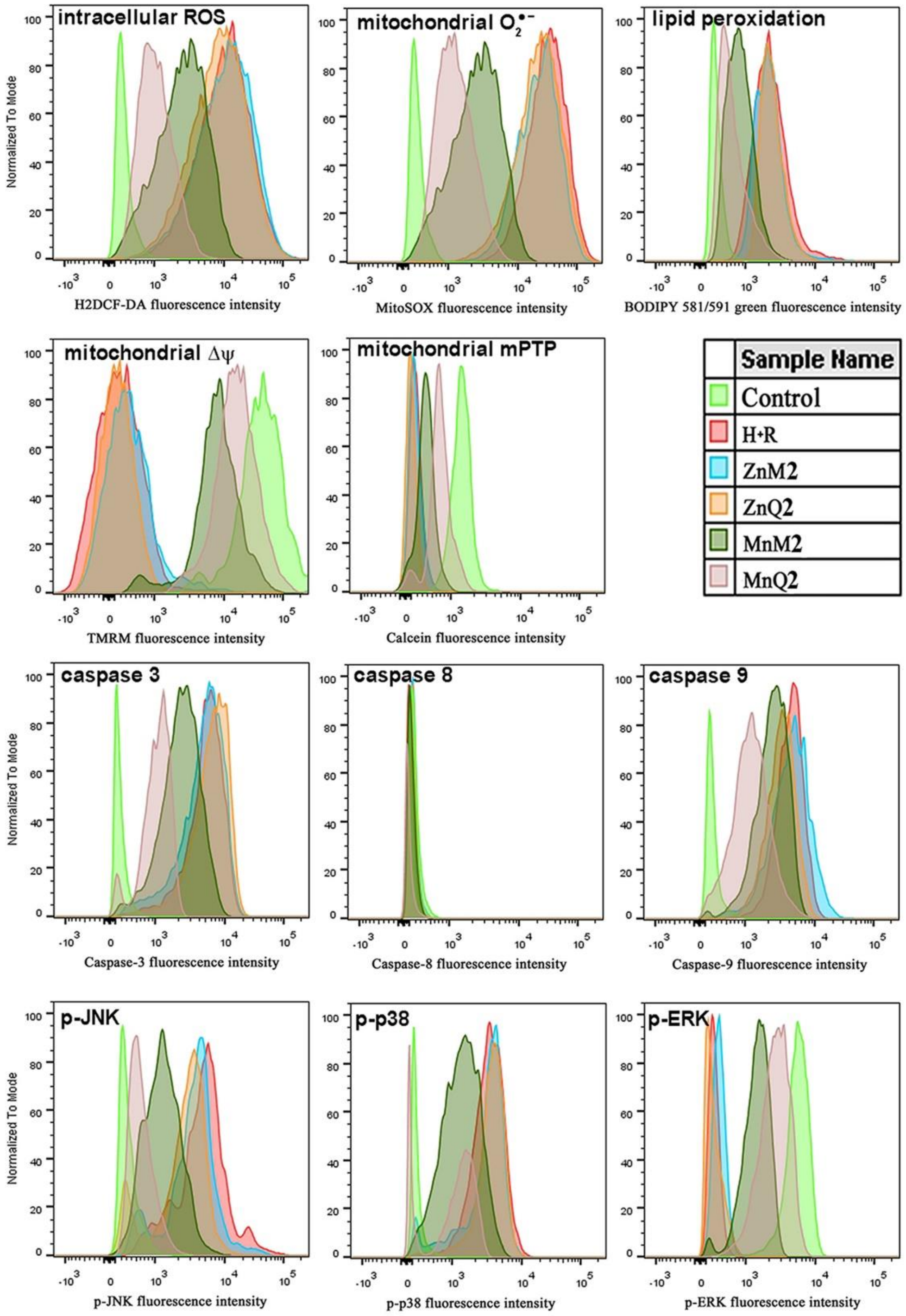

Supplement: Supplementary file 3 — Supplementary information 3 [file 41598_2019_46476_MOESM3_ESM.pdf]
